# Construction and analysis of gene-gene dynamics influence networks based on a Boolean model

**DOI:** 10.1186/s12918-017-0509-y

**Published:** 2017-12-21

**Authors:** Maulida Mazaya, Hung-Cuong Trinh, Yung-Keun Kwon

**Affiliations:** Department of Electrical/Electronic and Computer Engineering, University of Ulsan, 93 Daehak-ro, Nam-gu, Ulsan, 44610 Republic of Korea

**Keywords:** Boolean dynamics, Gene-gene dynamics influence (GDI), Gene-gene molecular interaction (GMI) networks, Knockout mutation, Structural characteristics

## Abstract

**Background:**

Identification of novel gene-gene relations is a crucial issue to understand system-level biological phenomena. To this end, many methods based on a correlation analysis of gene expressions or structural analysis of molecular interaction networks have been proposed. They have a limitation in identifying more complicated gene-gene dynamical relations, though.

**Results:**

To overcome this limitation, we proposed a measure to quantify a gene-gene dynamical influence (GDI) using a Boolean network model and constructed a GDI network to indicate existence of a dynamical influence for every ordered pair of genes. It represents how much a state trajectory of a target gene is changed by a knockout mutation subject to a source gene in a gene-gene molecular interaction (GMI) network. Through a topological comparison between GDI and GMI networks, we observed that the former network is denser than the latter network, which implies that there exist many gene pairs of dynamically influencing but molecularly non-interacting relations. In addition, a larger number of hub genes were generated in the GDI network. On the other hand, there was a correlation between these networks such that the degree value of a node was positively correlated to each other. We further investigated the relationships of the GDI value with structural properties and found that there are negative and positive correlations with the length of a shortest path and the number of paths, respectively. In addition, a GDI network could predict a set of genes whose steady-state expression is affected in *E. coli* gene-knockout experiments. More interestingly, we found that the drug-targets with side-effects have a larger number of outgoing links than the other genes in the GDI network, which implies that they are more likely to influence the dynamics of other genes. Finally, we found biological evidences showing that the gene pairs which are not molecularly interacting but dynamically influential can be considered for novel gene-gene relationships.

**Conclusion:**

Taken together, construction and analysis of the GDI network can be a useful approach to identify novel gene-gene relationships in terms of the dynamical influence.

**Electronic supplementary material:**

The online version of this article (10.1186/s12918-017-0509-y) contains supplementary material, which is available to authorized users.

## Background

Gene-gene relationships have been investigated for a long time in many previous studies [1–5]. In particular, most attention was focused on the functional properties of protein-protein interaction networks [6–8] or an epistasis which means that masking a particular allele prevents effects of another gene at a different locus [9, 10], and many methods based on statistical correlation analysis over gene expression datasets were developed to reveal a new epistasis [11–16]. However, they have a limitation in identifying more complicated gene-gene relationships because a state of a gene can be affected by many other genes along various signaling pathways [10]. In this regard, network-based approaches have been proposed [2, 11, 17, 18] and found more complicated forms of gene-gene relationships such as feedback and feed-forward loops. These approaches often inferred false gene-gene relationships, though, because they were based on only the analysis of a network structure without considering network dynamics [19]. Accordingly, an investigation of novel gene-gene relationships in terms of the network dynamics was necessarily needed.

To this end, we proposed a method to quantify gene-gene dynamics influence (GDI) in this study. We computed how much a state trajectory of a gene is changed by a knockout mutation subject to another gene in a gene-gene molecular interaction (GMI) network using a Boolean network model [20–24]. By examining the GDI values of every ordered pair of genes, we can construct a GDI network where each directed edge indicates a positive dynamical influence from the source gene to the target gene of the edge. This notion can be regarded as an extension of previous studies about effects of genetic mutations on network dynamics. For example, it was shown that a single gene mutation can change a communication pattern between genes [25], which can lead to human diseases in gene regulatory networks [26, 27] or dysfunctional mechanism in T-cell survival signaling network [28, 29]. In our study, we analyzed properties of the GDI networks induced from real GMI networks. Through a comparison of the topologies between the GMI and the GDI networks, we found that the latter network was denser than the former, which implies that there exist a lot of gene pairs with a dynamically influencing but molecularly non-interacting relation. We further analyzed the degree distributions of large-scale GMI and GDI networks. We found that they are considerably different from each other because a lot of hub genes were generated in the latter network. Despite this difference with respect to connectivity, it was interesting to observe that the degree of a node in the GDI network is positively correlated to that in the GMI network. To deepen our understating about the structure of the GDI networks, we examined the relations of well-known structural properties to the GDI value and found that the length of a shortest path and the number of paths of a gene pair have negative and positive correlations, respectively, to the GDI value whereas the number of feedback loop showed no significant relation. In addition, we observed that a GDI network can predict a high proportion of genes of which the steady-state expression was changed in *E. coli* gene-knockout experiments. More interestingly, we observed that the drug-targets with side-effects in the GDI network have a larger number of outgoing links and a smaller number incoming links than the rest of genes. This implies that the drug-targets with side-effects are more likely to influence the dynamics of other genes, but unlikely to be influenced by other genes. Finally, we found biological evidences supporting that the gene pairs which are not molecularly interacting but dynamically influential can be considered for novel gene-gene relationships which were not identified by traditional approaches yet. Taken together, construction of a GDI network can be a useful approach to explain various dynamical behavior induced by complex gene-gene relations in large-scale GMI networks.

## Methods

### Datasets

To investigate the GDI networks, we used three datasets about the GMI networks such as an *Arabidopsis* morphogenesis regulatory network (AMRN) with 10 nodes and 20 interactions [30], a *guard* cell abscisic acid signaling network (ABAN) with 44 nodes and 78 interactions [31], and a human signaling network (HSN) with 1609 nodes and 5063 interactions [32, 33] after removing self-loop interactions from the original datasets (see Additional file 1: Tables S1-S3). Moreover, we classified all genes in HSN into non-drug targets, and drug targets with and without side effects by using a drug target database of DrugBank [34] and a side-effect information database of SIDER [35, 36] (see Additional file 1: Table S4).

### A Boolean network model

In this work, we employed a Boolean network model to compute the GDI value. A Boolean network is one of the simplest computational models to describe network dynamics [37, 38], and has been generally used to investigate complicated behaviors of GMI networks [31, 39–41], which is represented by a directed graph *G* = (*V*, *A*) where *V* = {*v*_1_, *v*_2_, …, *v*_*N*_} is a set of nodes and *A* is a set of ordered pairs of the nodes called directed links (|*V*| and |*A*| denote the number of nodes and links, respectively). A directed link (*v*_*i*_, *v*_*j*_) ∈ *A* represents a positive (activating) or a negative (inhibiting) regulation from *v*_*i*_ to *v*_*j*_. Every *v*_*i*_ ∈ *V* has a state value with 1 (on) or 0 (off). The state of *v*_*i*_ at time *t* + 1 denoted by *v*_*i*_(*t* + 1) is established by the values of *k*_*i*_ other nodes ${v}_{i_1},{v}_{i_2},\dots, {v}_{i_{k_i}}$ with a link to *v*_*i*_ at time *t* by a Boolean function ${f}_i:{\left\{0,1\right\}}^{k_i}\to \left\{0,1\right\}$ and the states of all nodes are synchronously updated. Here, we implemented a nested canalyzing function (NCF) model [20, 42] to describe an update rule as follows:

$$ {f}_i\left({v_i}_1(t),{v_i}_2(t),\dots, {v_i}_{k_i}(t)\right)=\left\{\begin{array}{c}{O}_1\kern2.25em if\ {v}_{i_1}(t)={I}_1\kern22em \\ {}{O}_2\kern2.25em if\ {v}_{i_1}(t)\ne {I}_1\  and\ {v}_{i_2}(t)={I}_2\kern14.25em \\ {}{O}_3\kern2.25em if\ {v}_{i_1}(t)\ne {I}_1\  and\ {v}_{i_2}(t)\ne {I}_2\  and\ {v}_{i_3}(t)={I}_3\kern6.75em \\ {}\vdots \\ {}{O}_{k_i}\kern2em if\ {v}_{i_1}(t)\ne {I}_1\  and\cdots and\ {v}_{i_{k_i-1}}(t)\ne {I}_{k_i-1}\  and\ {v}_{i_{k_i}}(t)={I}_{k_i}\\ {}{O}_{def}\kern1.25em otherwise\kern23.5em \end{array}\right. $$

where all *I*_*m*_ and *O*_*m*_ (*m* = 1, 2, ⋯, *k*_*i*_) denote the canalyzing and canalyzed Boolean values, respectively, and *O*_*def*_ is set to $1-{O}_{k_i}$ in general. In this paper, each NCF is randomized by specifying every *I*_*m*_ and *O*_*m*_ between 0 and 1 uniformly at random. We note that many molecular interactions were successfully represented by NCFs [43–45].

A *network state* at time *t* can be denoted by an ordered list of state values of all nodes, **v**(*t*) = [*v*_1_(*t*), *v*_2_(*t*), …, *v*_*N*_(*t*)] ∈ {0, 1}^*N*^. Every network state transits to another network state through a set of Boolean update functions *F* = {*f*_1_, *f*_2_, …, *f*_*N*_}. Hence, a network state trajectory starting from an initial network state eventually converges to either a fixed-point or a limit-cycle attractor. We define the attractor more rigorously as follows.

#### Definition

Let **v**(0), **v**(1), ⋯,be a network state trajectory starting at **v**(0). The *attractor* is defined as an ordered list of network states 〈*G*, *F*, **v**(0)〉 = [**v**(*τ*), **v**(*τ* + 1), …, **v**(*τ* + *p* − 1)] where *τ* is the smallest time step such that **v**(*t*) = **v**(*t* + *p*) for ∀ *t* ≥ *τ* with **v**(*i*) ≠ **v**(*j*) for ∀ *i* ≠ *j* ∈ {*τ*, *τ* + 1, …, *τ* + *p* − 1} (herein, *p* is called a length of the attractor). In addition, the state sequences of *v*_*i*_ in 〈*G*, *F*, **v**(0)〉 is denoted by 〈*G*, *F*, **v**(0)〉_*i*_ = [*v*_*i*_(*τ*), *v*_*i*_(*τ* + 1), …, *v*_*i*_(*τ* + *p* − 1)].

Examination of attractors is required to compute the gene-gene dynamics influence in a network. To implement this, we specified a set of initial states (*S*) and computed a state trajectory starting at every **v**(0) ∈ *S* until an attractor is found. In the case of AMRN with a small number of nodes (|*N*| = 10), we could consider all 2^*N*^ possible states for *S*. Unfortunately, this exhaustive examination is not feasible to analyze a huge network. Therefore, we generated 2000 and 4000 random initial states to construct *S* in the case of ABAN (|*N*| = 44) and HSN (|*N*| = 1609), respectively.

### Construction of a GDI network

In this study, the dynamics influence of *v*_*i*_ on *v*_*j*_ for an ordered pair of genes (*v*_*i*_, *v*_*j*_) represents how much the states sequence of *v*_*j*_ is changed by a mutation subject to *v*_*i*_ in a Boolean network model. Specifically, we considered a knockout mutation [31, 46, 47] which describes a condition that the state of the mutated gene is frozen to 0 (off) state. This mutation can be implemented by changing *F* into *F* ^′^ which is defined as follows:

$$ {F}^{\prime }=\Big\{{\displaystyle \begin{array}{cc}\left\{{f}_1,\dots, {f}_{i-1},0,{f}_{i+1},\dots, {f}_N\right\}&, \forall t\le T\\ {}F&, \forall t>T\end{array}}\operatorname{} $$

where *T* is a parameter to denote the mutation duration time. In other words, the knockout mutation lasts for only ∀*t* ≤ *T*, and the update-rule of *v*_*i*_ is restored to *f*_*i*_ right after time step *T*. It was also shown that the mutation duration parameter can significantly affect the mutation process in complex GMI networks [48–50]. In the following, we explain how to compute the dynamics influence value from *v*_*i*_ to *v*_*j*_ denoted by *μ*(*v*_*i*_, *v*_*j*_) in detail.

1. Generate a set of random initial states *S*. For each initial state **v**(0) ∈ *S*, obtain two attractors 〈*G*, *F*, **v**(0)〉 and 〈*G*, *F* ^′^, **v**(0)〉 in the wild-type and the *v*_*i*_-mutant networks, respectively. For convenience, let 〈*G*, *F*, **v**(0)〉 = [**v**(*τ*), **v**(*τ* + 1), …, **v**(*τ* + *p* − 1)] and 〈*G*, *F* ^′^, **v**(0)〉 = [**v** ^′^(*τ* ^′^), **v** ^′^(*τ* ^′^ + 1), …, **v** ^′^(*τ* ^′^ + *p* ^′^ − 1)].
2. Compute a distance between 〈*G*, *F*, **v**(0)〉_*j*_ and 〈*G*, *F* ^′^, **v**(0)〉_*j*_ defined as follows

$$ d\left(\mathbf{v}(0),,,{v}_i,{v}_j\right)=\underset{m\in \left[0,d-1\right]}{\min}\frac{\sum \limits_{l=0}^{c-1}I\left({v}_j\left(\tau +l+m\right)\ne {v}_j^{\prime}\left({\tau}^{\prime }+l\right)\right)}{c} $$

where *c* and *d* are the least common multiple and the greatest common divisor, respectively, of *p* and *p* ^′^, and *I*(*condition*) is a function which outputs 1 if *condition* is true, and 0 otherwise. As a result, *d*(**v**(0), *v*_*i*_, *v*_*j*_) represents the minimum ratio of a bitwise difference between the states sequence of *v*_*j*_ in the wild-type and the *v*_*i*_-mutant attractors over the least common period (*c*) of the two attractors.

(3) Lastly, compute the dynamics influence of *v*_*i*_ on *v*_*j*_ denoted by *μ*(*v*_*i*_, *v*_*j*_) by averaging out *d*(**v**(0), *v*_*i*_, *v*_*j*_) over all initial states in *S* as follows:

$$ \mu \left({v}_i,{v}_j\right)=\frac{\sum \limits_{\mathbf{v}(0)\in S}d\left(\mathbf{v}(0),{v}_i,{v}_j\right)}{\mid S\mid } $$

**Fig. 1:**
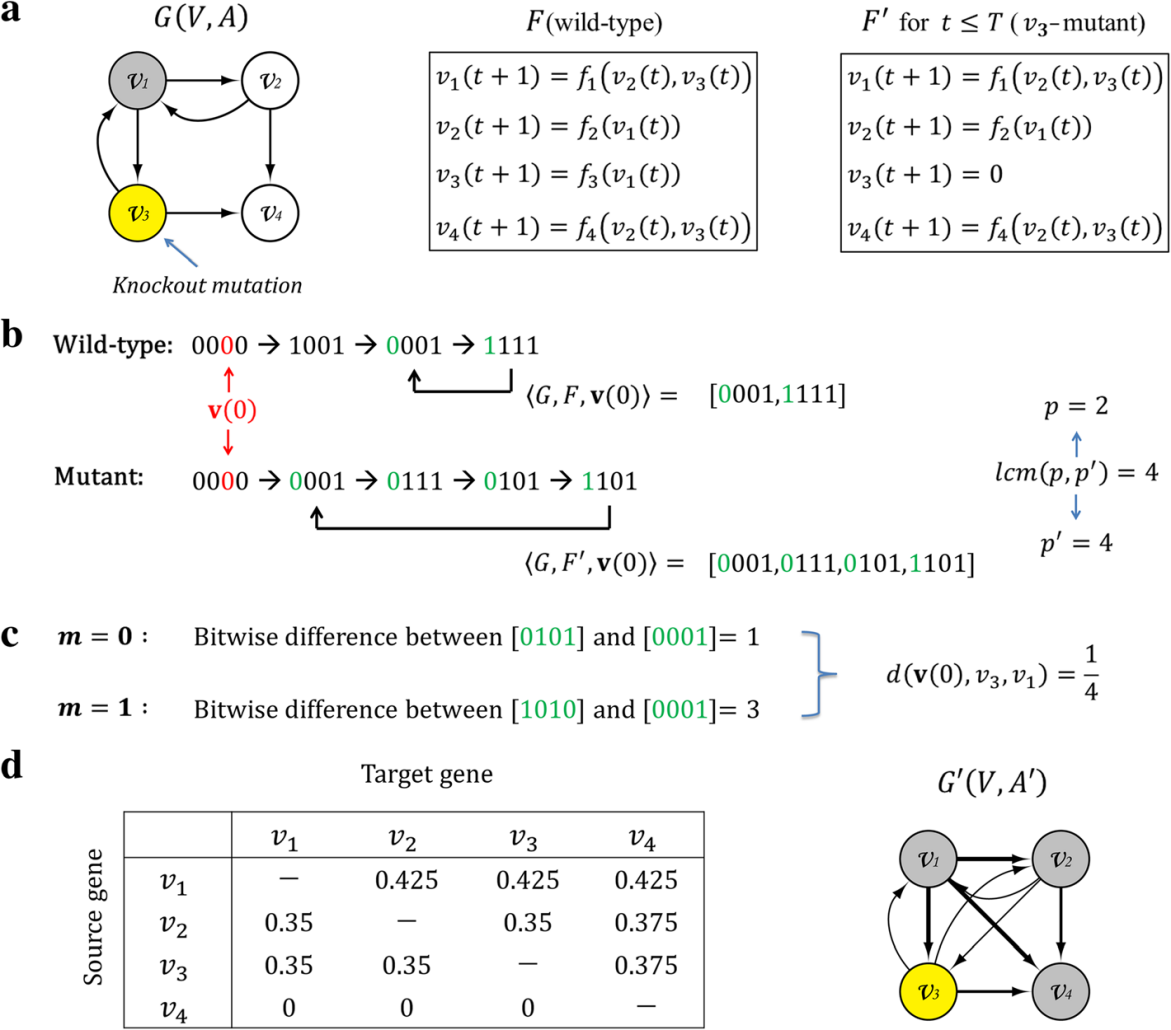
An illustrative example of computing the gene-gene dynamics influence value. **a** An example GMI network. Given a network *G*(*V*, *A*) with a set of update rules *F*, let *v*_3_ a node subjected to the knockout mutation for *t* ≤ *T*. The knockout mutation changes *F* into *F* ^′^ where the state value of *v*_3_ is frozen to 0 for *t* ≤ *T*. **b** Identification of wild-type and mutant attractors. Let [0000] ∈ *S* be an initial state considered in this example. By examining two state trajectories along with *F* and *F* ^′^, respectively, we obtain two corresponding attractors, 〈*G*, *F*, **v**(0)〉 and 〈*G*, *F* ^′^, **v**(0)〉 of which the *least common multiple* of the lengths is four. **c** Computation of a distance between wild-type and mutant attractors. Since the *greatest common divisor* of the lengths of two attractors is two, we examine two different alignments of the state sequences of *v*_1_ in those attractors. The number of different bits between [0101] and [0001] is 1 in case 1 (*m* = 0), whereas that between [1010] and [0001] is 3 in case 2 (*m* = 1). Accordingly, the minimum bitwise difference is 1, and hence $d\left(\mathbf{v}(0),{v}_3,{v}_1\right)=\frac{1}{4}$. We can compute *μ*(*v*_3_, *v*_1_) by averaging out *d*(**v**(0), *v*_3_, *v*_1_) over the set of initial states. **d** The resultant GDI network. The left matrix shows the dynamics influence value for every ordered pair of genes, and the right graph shows the resultant GDI network with nine positive dynamics influence relations

Figure 1 shows an illustrative example to compute the GDI value in a network where node *v*_3_ out of four nodes is subject to the knockout mutation. The set of update rules *F* is modified into *F* ^′^ where state value of *v*_3_ is frozen to 0 (Fig. 1a) during a mutation duration time *T*, and we can obtain the wild-type and *v*_3_-mutant attractors (Fig. 1b). To compute the dynamical influence on node *v*_1_, we compute the minimum bitwise difference between the state sequence of *v*_1_ in two attractors considering all possible alignments of the sequences (Fig. 1c), and eventually obtain the distance between 〈*G*, *F*, **v**(0)〉_1_ and 〈*G*, *F* ^′^, **v**(0)〉_1_. Then *μ*(*v*_3_, *v*_1_) is the average distance over the set of different initial states. Based on this measure, we can construct a GDI network *G* ^′^(*V*, *A* ^′^) from a GMI network *G*(*V*, *A*) by calculating *μ*(*v*_*i*_, *v*_*j*_) for every ordered gene pair (*v*_*i*_, *v*_*j*_). More specifically, a GDI network is a directed graph where (*v*_*i*_, *v*_*j*_) ∈ *A* ^′^ if and only if *μ*(*v*_*i*_, *v*_*j*_) > 0. In other words, a directed edge in a GDI network means that the state sequences of the target node are changed by the knockout mutation subject to the source node of the edge. Figure 1d shows a matrix of *μ*(*v*_*i*_, *v*_*j*_) values for every ordered gene pair (*v*_*i*_, *v*_*j*_) and a resultant GDI network with nine positive influence relations.

### Structural characteristics of networks

In real GMI networks, some structural characteristics of genes and interactions have been shown to be relevant to sustainability of network dynamics [51–53]. In this regard, we employed the following well-known structural properties to investigate their relationships with the GDI value.

- The length of a shortest path for an ordered gene pair (*v*_*i*_, *v*_*j*_), denoted by *l*(*v*_*i*_, *v*_*j*_), means the number of edges included in a shortest path from *v*_*i*_ to *v*_*j*_ [23, 40].
- The number of paths for an ordered gene pair (*v*_*i*_, *v*_*j*_), denoted by *n*(*v*_*i*_, *v*_*j*_), means the number of different paths from *v*_*i*_ to *v*_*j*_ [40, 54].
- The number of feedback loops for an ordered gene pair (*v*_*i*_, *v*_*j*_), denoted by *f*(*v*_*i*_, *v*_*j*_), means the number of feedback loops involving both *v*_*i*_ and *v*_*j*_ [22, 26]. A feedback loop is a circular chain of nodes where any node is not revisited except both end nodes. Specifically, *u*_1_ → *u*_2_ → … → *u*_*L*_ is a feedback loop of length *L*(>1) if there exists a link from *u*_*i*_ to *u*_*i* + 1_(*i* ∈ {1,  …, *L* − 1}) such that *u*_1_ = *u*_*L*_ and *u*_*j*_ ≠ *u*_*k*_ for ∀*j* ≠ *k* ∈ { 1, …, *L* − 1}.

### Construction of random networks

To verify that the results found in the real GMI networks hold in randomly structured networks, we generated random networks by using two models, the Barabási Albert (BA) model [55] and the shuffling model [41] (see Additional file 1: Figures S1 and S2, respectively, for the pseudo-codes), and analyzed their corresponding GDI networks. The BA model generates a random network using a preferential attachment scheme which is a network growth model. On the other hand, the shuffling model creates a random network by rewiring the links of a GMI network in a way that both in- and out-degrees of every node are preserved. Accordingly, the latter can generate a random network whose structure is more similar with the GMI network than the former.

## Results

We generated the GDI networks from each of three real GMI networks, AMRN, ABAN, and HSN (see Methods). For convenience, we denote a GMI network and a corresponding GDI network by *G*(*V*, *A*) and *G* ^′^(*V*, *A* ^′^), respectively.

### Topological comparison between GMI and GDI networks

**Fig. 2:**
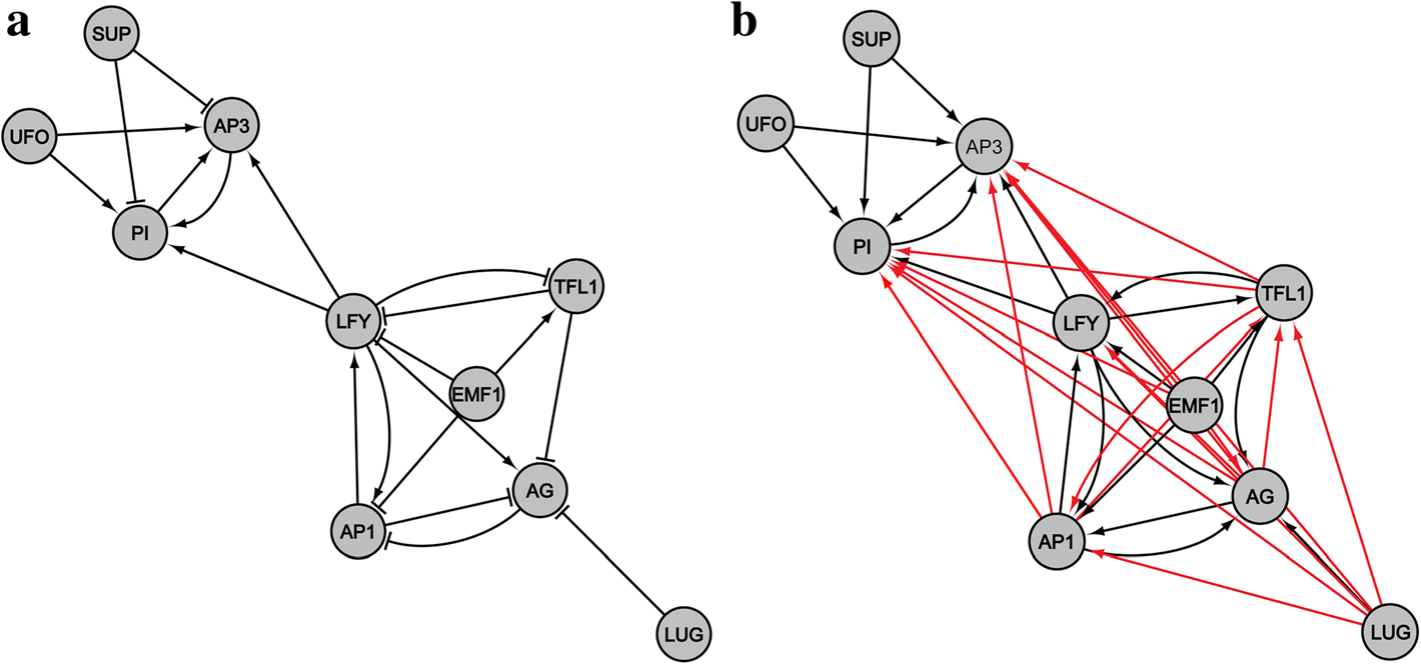
Visualization of the GMI and the corresponding GDI networks in the case of AMRN. **a** The GMI network with |*V*| = 10 and |*A*| = 20. Arrow-headed and bar-headed lines indicate activating (positive) and inhibitory (negative) interactions, respectively. **b** The corresponding GDI network with |*V*| = 10 and |*A* ^′^| = 38. The gene pairs belonging to MIDI and MNDI groups are represented by black and red colored links, respectively. There was no gene pair belonging to MIDN group

To investigate a topological difference between the GMI and the corresponding GDI networks, we first visualized them (Fig. 2 for the result of AMRN; see Additional file 1: Figures S3-S4 for the results of ABAN and HSN). For a further analysis, we classified every ordered pair of genes (*v*_*i*_, *v*_*j*_) into three groups as follows: a group of molecularly interacting and dynamically influential (MIDI) gene pairs (i.e., (*v*_*i*_, *v*_*j*_) ∈ *A* and (*v*_*i*_, *v*_*j*_) ∈ *A* ^′^), a group of molecularly non-interacting but dynamically influential (MNDI) gene pairs (i.e., (*v*_*i*_, *v*_*j*_) ∉ *A* and (*v*_*i*_, *v*_*j*_) ∈ *A* ^′^), and a group of molecularly interacting but dynamically non-influential (MIDN) gene pairs (i.e., (*v*_*i*_, *v*_*j*_) ∈ *A* and (*v*_*i*_, *v*_*j*_) ∉ *A* ^′^). Table 1 shows the numbers of gene pairs belonging to MIDI, MNDI, and MIDN groups. We observed that the number of links of the GDI network was larger than that of the GMI network, which is because MNDI gene pairs are more frequently found than MIDN gene pairs (for example, we found 18 MNDI but no MIDN gene pairs in the case of AMRN). It is also interesting to observe a considerably large number of MIDN gene pairs in ABAN and HSN, because this implies that a molecularly interacting gene pair does not always induce a dynamically influencing relation. We note that the number of MIDN gene pairs in HSN was even larger than twice that of MIDI gene pairs.

**Table 1:** The number of gene pairs in groups classified by comparing the GMI and the GDI networks

	AMRN	ABAN	HSN
The number of MIDI gene pairs (A)	20	63	1311
The number of MIDN gene pairs (B)	0	15	3752
The number of MNDI gene pairs (C)	18	603	19,910
The number of links in the molecular interaction network (A + B)	20	78	5063
The number of links in the GDI network (A + C)	38	666	21,221

**Fig. 3:**
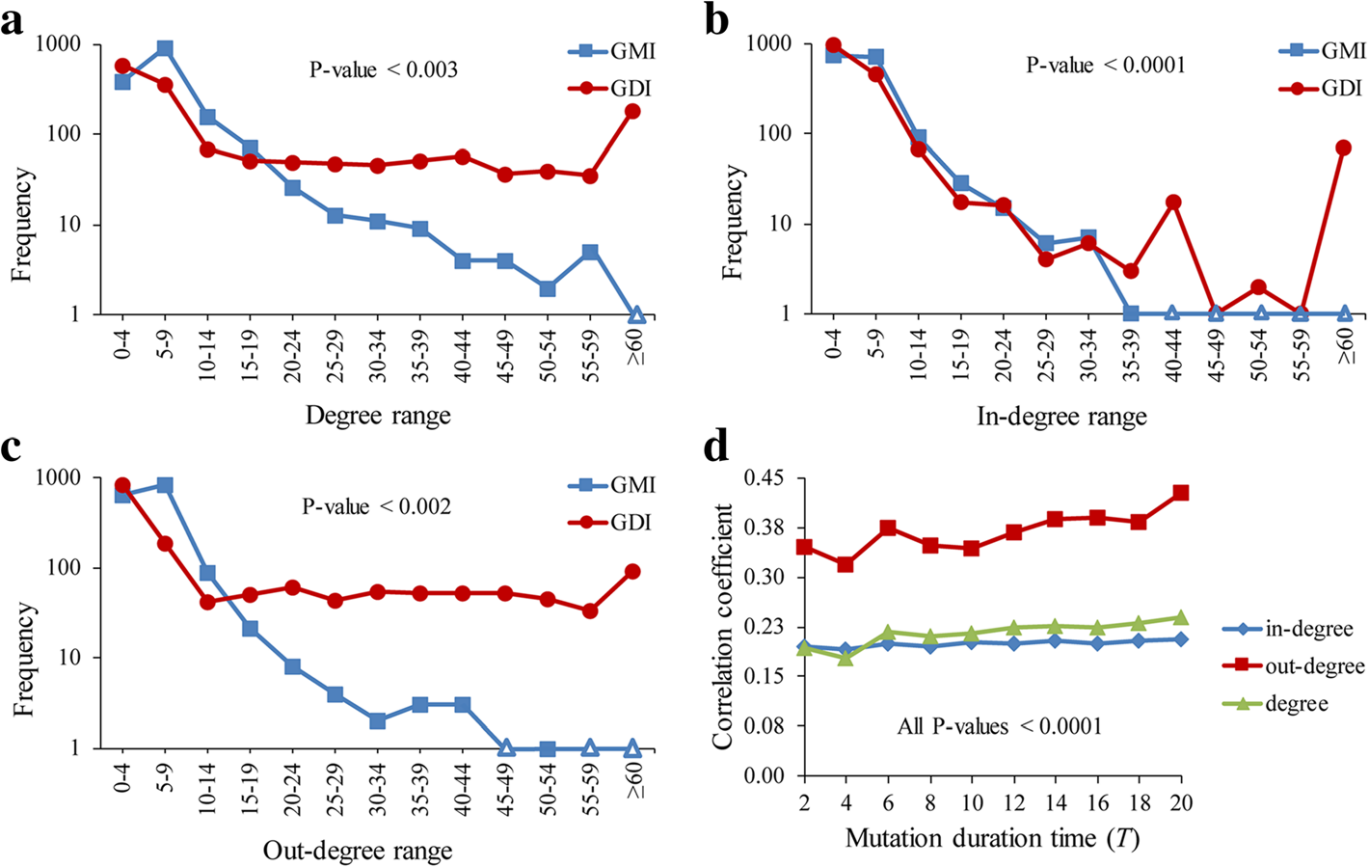
Degree distributions of the GMI and the corresponding GDI networks in HSN. **a**-**c** Results of degree, in-degree, and out-degree distributions, respectively. The mutation duration time (*T*) was set to 20 in generating the GDI network. Y-axis represents a log-scaled frequency and blank triangle points (Δ) mean zero value of a frequency. We observed that the proportions of a same bin in GMI and GDI networks are significantly different from each other for most ranges (All *P*-values were less than 0.003 in (**a**) except for degree range ‘0–4’, less than 0.0001 in (**b**) except for in-degree range ‘0–4’, and were less than 0.002 in (**c**) except for out-degree range ‘0–4’. **d** Correlation coefficients between degree/in-degree/out-degree values of a node in the GMI and the GDI networks. The mutation duration time was varied from 2 to 20. Each of degree, in-degree, and out-degree of a node in the GDI network showed a significant positive relationship with that in the GMI network (All *p*-values <0.0001)

We further compared the GMI and the GDI networks with respect to the degree distributions. Considering the network size, we investigated the case of HSN only (Fig. 3). We found that the degree of the GMI network considerably follows a power-law distribution whereas that of the GDI network does not (Fig. 3a). In particular, the hub nodes with a relatively high degree were more abundant in the GDI network than in the GMI network. Through additional comparisons with respect to the in-degree and the out-degree distributions (Fig. 3b and c, respectively), we found that the difference of the out-degree distribution was larger than that of in-degree distribution. All these results indicate that the overall topology of the GDI network is considerably different from that of the GMI network. We further wondered if a degree of a node in the GDI network is related or not to that in the GMI network. To answer this question, we compared the correlation coefficients between degree/in-degree/out-degree values of a node in the GMI and the GDI networks (Fig. 3d). As shown in the figure, each of them showed a significant positive correlation, irrespective of the mutation duration time. This means that the degree/in-degree/out-degree of a node in the GDI network is likely to be larger as that in the GMI network gets larger. Taken together, the topology of a GMI network can be partially helpful in predicting the topology of a GDI network although the latter is denser than the former.

### Relation of dynamics influence values with structural characteristics in GDI networks

**Fig. 4:**
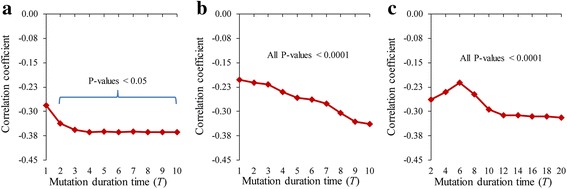
Relationship of the GDI value to the length of a shortest path in the GDI networks. **a**-**c** Results of AMRN, ABAN, and HSN, respectively. Y-axis values mean the correlation coefficients between *μ*(*v*_*i*_, *v*_*j*_) and *l*(*v*_*i*_, *v*_*j*_) for all ordered pairs of genes. The mutation duration time was varied from 1 to 10 in (**a**) and (**b**), and from 2 to 20 in (**c**)

**Fig. 5:**
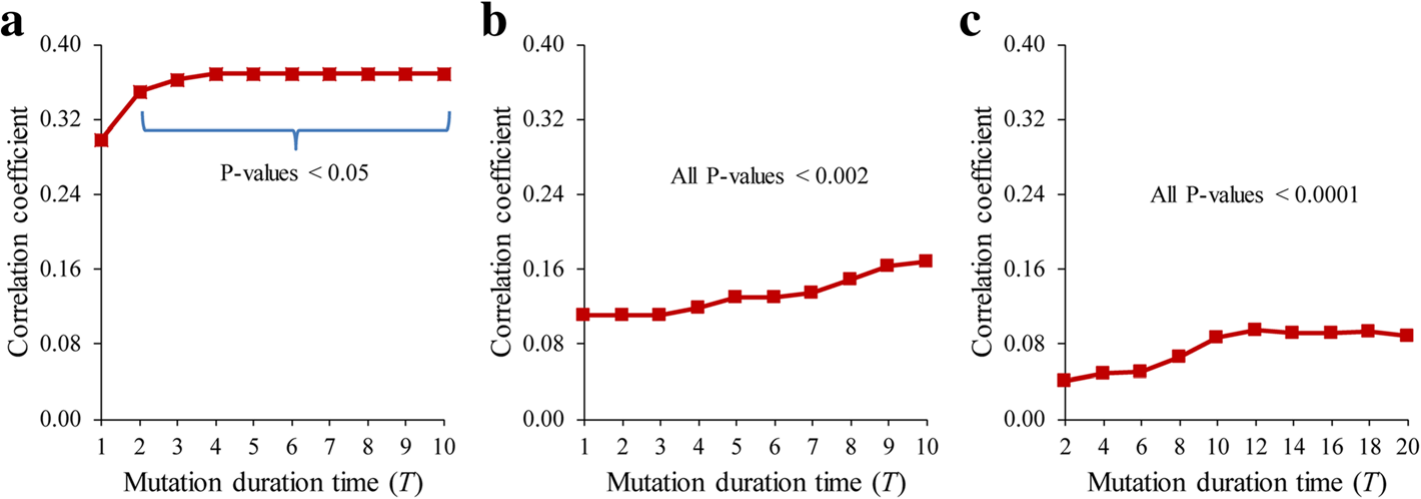
Relationship of the GDI value to the number of paths in the GDI networks. **a**-**c** Results of AMRN, ABAN, and HSN, respectively. Y-axis values mean the correlation coefficients between *μ*(*v*_*i*_, *v*_*j*_) and *n*(*v*_*i*_, *v*_*j*_) for all ordered pairs of genes. The mutation duration time was varied from 1 to 10 in (**a**) and (**b**), and from 2 to 20 in (**c**)

To discover a network-based principle about the GDI value, we investigated some structural properties in the GDI networks. Here, we considered the relationships of the GDI value of a directed edge (*μ*(*v*_*i*_, *v*_*j*_)) to three edge-based structural properties, the length of a shortest path from *v*_*i*_ to *v*_*j*_ (*l*(*v*_*i*_, *v*_*j*_)), the number of paths from *v*_*i*_ to *v*_*j*_ (*n*(*v*_*i*_, *v*_*j*_)), and the number of feedback-loops involving *v*_*i*_ and *v*_*j*_ (*f*(*v*_*i*_, *v*_*j*_)) in the GDI networks (see Methods for the definitions). We considered these three structural properties because they have been frequently used to show structural characteristics of functionally important genes or interactions in signaling networks [52, 53]. Figure 4 shows the correlation coefficients between *μ*(*v*_*i*_, *v*_*j*_) and *l*(*v*_*i*_, *v*_*j*_) in the GDI networks, and they showed significant negative relations, irrespective of the mutation duration time. In other words, the dynamics influence of *v*_*i*_ on *v*_*j*_ is likely to be higher as the length of a shortest path from *v*_*i*_ to *v*_*j*_ is shorter. We infer that the information flow from the source gene to the target gene in the pair is less interfered by other genes when they are connected by a path of a short length. We note that this result is relevant to a previous study having shown that diseases whose associated genes are connected by a relatively short path tend to be comorbid [40]. In addition, the negative relation was more obvious as the duration time increases. To clarify that this finding is a general principle, we examined the correlation coefficients between *μ*(*v*_*i*_, *v*_*j*_) and *l*(*v*_*i*_, *v*_*j*_) in two types of random networks generated by the shuffling and BA models (see Methods), and could observe the consistent results (see Additional file 1: Figure S5). To find another structural property, we examined the correlation coefficients between *μ*(*v*_*i*_, *v*_*j*_) and *n*(*v*_*i*_, *v*_*j*_) (Fig. 5), and found significant positive relations, irrespective of the mutation duration time. This means that the dynamics influence of *v*_*i*_ on *v*_*j*_ tends to be higher as a larger number of paths connect from *v*_*i*_ to *v*_*j*_. We infer that the information flow from the source gene to the target gene in the pair is more reinforced when they are connected by a larger number of paths. In addition, we examined the correlation coefficients between *μ*(*v*_*i*_, *v*_*j*_) and *n*(*v*_*i*_, *v*_*j*_) in both the shuffled and the BA random networks, and could observe the consistent results (see Additional file 1: Figure S6). This implies that the positive relation between the number of paths and the dynamics influence can be a general property in various structural networks. Finally, we examined the relationship between *μ*(*v*_*i*_, *v*_*j*_) and the number of feedback loops involving the gene pair *f*(*v*_*i*_, *v*_*j*_) (see Additional file 1: Figure S7) and found no consistently significant relationships in both real GMI networks and the random networks. Considering that the feedback loop structure was successfully used to predict functionally important genes or interactions [22, 26], our finding implies that the structural characteristics in the GDI networks can be different from those in the GMI networks.

### Comparison of GDI network with knockout experiments

To validate our approach, we investigated how much a GDI network is consistent to real knockout experiments. To this end, we used an *E. coli* microarray dataset (E_coli_v4_Build_6 version) from the Many Microbe Microarrays database (M3D) [56] which contains the expression levels of 4297 genes from 446 samples. In addition, we also employed the RegulonDB database [57] which contains the information about the transcriptional regulations of *E. coli*. We integrated these two databases to identify the set of common genes, and then constructed a GMI network of *E. coli* with 1424 genes and 3114 edges. From the GMI network, we also generated the corresponding GDI network of *E. coli*, and denoted a set of out-going genes from a gene *g* by *GDI*_*O*_(*g*). To compare the GDI network result with the real knockout experiments, we identified 7 genes of which real knockout experimental results were included in both the GDI network and M3D database (There were 21 knockouts and 9 relevant wild-type experiments in M3D database). We converted real-valued expression to Boolean-valued one by using a discretization method based on K-means clustering algorithm [58]. It assigns 1 (the ‘on’ state) and 0 (the ‘off’ state) if the expression value is larger and lower, respectively, than the average expression of a gene. For each mutant gene *g*, we denote by *EXP*_*O*_(*g*) a set of genes of which Boolean expression values are differently observed between the knockout and the wild-type experiments. In other words, *GDI*_*O*_(*g*) and *EXP*_*O*_(*g*) represent a set of genes of which dynamics are affected by a knockout mutation at gene *g* through the GDI network analysis and the real experiments, respectively. To assess how much proportion of *EXP*_*O*_(*g*) is predicted by *GDI*_*O*_(*g*), we examined a precision ratio defined as follows:

$$ ratio(g)=\frac{\mid {EXP}_O(g)\cap {GDI}_O(g)\mid }{\mid {EXP}_O(g)\mid } $$

As shown in Table 2, we found that the ratio ranges from 0.35 to 0.63 except for two genes, cspA and appY. This implies that the GDI network analysis can predict a relatively high portion of genes of which the expression was changed by the knockout experiment, although it did not explain all the experiments. This partially supports the validation of the GDI network-based analysis.

**Table 2:** Comparison between two sets of knockout-affected genes identified through a GDI network and *E-coli* knockout experiment, respectively

Knocked-out gene (*g*)	The number of genes in *EXP*_*O*_(*g*)	The number of genes in *GDI*_*O*_(*g*)	The number of genes in *EXP*_*O*_(*g*) ∩ *GDI*_*O*_(*g*)	*Ratio*(*g*)
cspA	151	2	0	0.00
soxS	199	524	70	0.35
oxyR	235	539	97	0.41
appY	250	9	5	0.02
arcA	311	453	111	0.35
crp	342	891	217	0.63
fnr	502	616	263	0.52

### Analysis of drug-target genes based on GDI networks

**Fig. 6:**
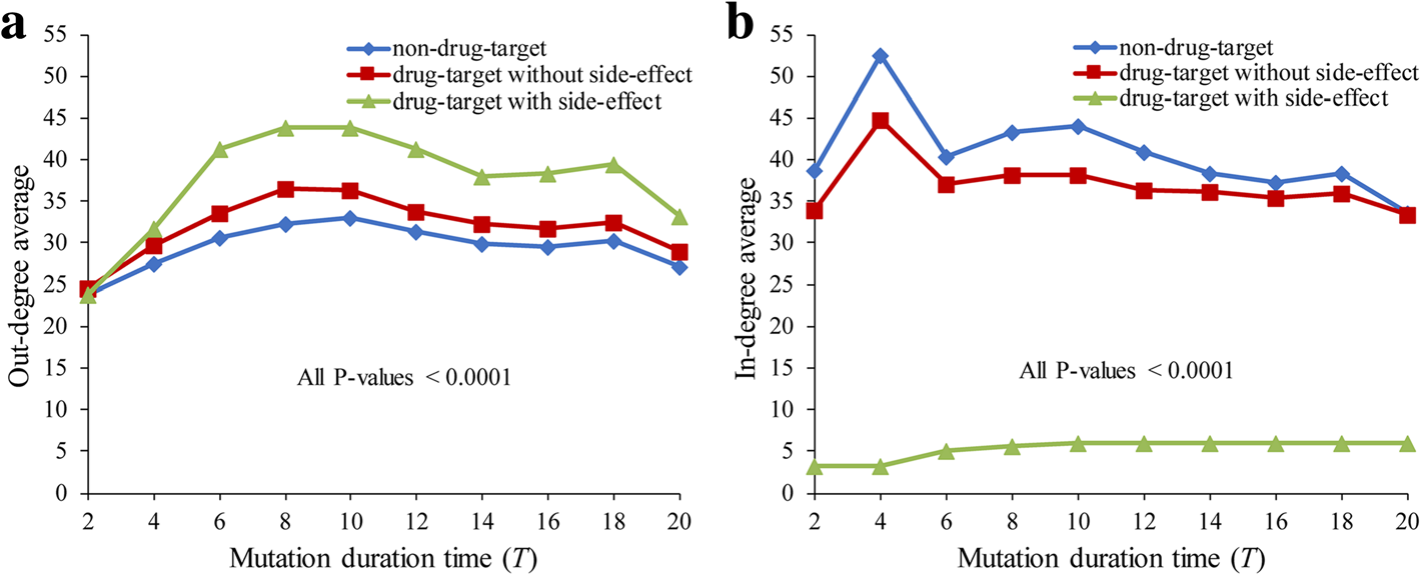
Out-degree and in-degree comparisons between non-drug targets and drug-targets in GDI network derived from HSN. **a** Result of out-degree. The average out-degree of the drug-targets with side-effects was larger than those of non-drug targets and drug-targets without side-effects in every mutation duration time except for *T* = 2 (All *P*-values <0.0001). **b** Result of in-degree. Non-drug target, drug-target without side-effect, and drug-target with side-effect are represented by blue, red, and green color, respectively. The average in-degree of the drug-targets with side-effects was smaller than those of non-drug targets and drug-targets without side-effects in every mutation duration time (All *P*-values <0.0001). The mutation duration time was varied from 2 to 20

Some previous approaches investigated drug-target genes through network structure analysis [59, 60]. For example, it was found that drug-target genes are more centrally located as well as more evolutionary than non-drug target genes [61]. It was also shown that the connectivity of drug-targets was significantly different from that of non-drug targets [62, 63]. Inspired by these results, we examined the structural characteristics of drug-targets in the GDI network. More specifically, we classified all genes in HSN into three groups of non-drug targets, drug-targets without side-effects, and drug-targets with side-effects (see Methods). We examined the average in- and out-degrees of three groups in the GDI network derived from the GMI network of HSN (Fig. 6). As shown in the figure, the average out-degree (in-degree) of the drug-targets with side-effects was larger (resp., smaller) than those of non-drug targets and drug-targets without side-effects, almost irrespective of the mutation duration time. In other words, the drug-targets with side-effects are more likely to influence other genes, whereas they are less likely to be influenced by other genes. This result supports some experimental studies having shown that drug-targets with side-effects have a relatively larger impact on other genes than non-drug targets [60, 62, 63]. This case study supports the usefulness of GDI network analysis.

### Biological evidence of novel gene-gene relations

To reveal novel gene-gene relations by means of the GDI network analysis, we profiled the gene pairs which are included in the GDI network but not included in the GMI network (i.e., gene pairs of MNDI group found in Table 1, Fig. 2, and see Additional file 1: Figures S3-S4), and some of them were listed in Table 3. Interestingly, we could find some biological evidences relevant to the gene pairs in the table. For example, the relation from EMF1 to AG found in AMRN can explain that EMF1 played an important role in maintaining AG development in *A. thaliana* [16, 64–66], and the relation from TFL1 to AP1 can explain that the addition of the TFL1 mutation induces the AP1 mutant which changes the *phyllotaxy* of lateral flowers [67]. In addition, it was reported in ABAN that ABA gene cooperates with S1P on slow anion channels [24, 68] or induces NO productions abolished in either NOS or NIA12 [31]. We also note previous studies having shown the CSK mutant can affect a regulatory polymorphism in B-cell signaling [69, 70] or the complement relationship of GHR and IGF1R [71]. These evidences imply that the GDI network-based analysis can reveal novel gene-gene relations which are not well-known yet.

**Table 3:** Example of gene pairs which are molecularly non-interacting but dynamically influential (MNDI)

Network	Gene pairs	Sub-graph	*μ*(*v*_*i*_, *v*_*j*_)	*l*(*v*_*i*_, *v*_*j*_)
AMRN	EMF1, AG	EMF1→ TFL1⊣ AG	0.146	2
	TFL1, AP1	TFL1⊣ LFY→ AP1	0.0125	2
ABAN	ABA, S1P	ABA→ SphK→ S1P	0.498	2
	ABA, NIA12	ABA→ RCN1→ NIA12	0.498	2
HSN	CSK, BCR	CSK⊣ LYN→ BCR	0.503	2
	GHR, IGF1R	GHR→ IGF1→ IGF1R	0.382	2

Source and target genes are represented by orange- and green-colored nodes, respectively. Sub-graph field means the shortest path with respect to the gene pair in the GMI network. Arrow-headed (→) and bar-headed (⊣) lines indicate activating (positive) and inhibitory (negative) interactions, respectively

## Results and discussion

Gene-gene relationships have been investigated in many studies, most of which focused on epistasis and statistical correlation analysis. However, they have a limitation in identifying more complicated relationships and hence some network-based approaches have been proposed to overcome it. It is a still open problem because they did not incorporate analysis about the dynamical relationships. In this regard, we first proposed a measure to quantify the gene-gene dynamics influence using a Boolean network model and eventually constructed a GDI network. To find characteristics of the GDI network, we compared the topologies of the GMI and the GDI networks and observed that the latter is denser than the former. This was because a lot of hub nodes were generated in the GDI network. In addition, the degree distributions were also different between them. Despite these topological differences, we found an interesting similarity such that the degree value of a node was positively correlated between the GMI and the GDI networks. For further investigations about the structure of the GDI networks, we examined the relations of three structural properties to the GDI value, and found that the length of a shortest path and the number of paths have negative and positive correlations, respectively, whereas the number of feedback loop showed no relation. In addition, we observed that a GDI network could predict a set of genes whose steady-state expression is affected in *E. coli* gene-knockout experiments. It was more intriguing to observe that the drug-targets with side-effects are more likely to influence the dynamics of other genes, but less likely to be influenced by other genes through the GDI network-based analysis. We note that it is possible to reveal novel gene-gene relationships by considering gene pairs which are not molecularly interacting but dynamically influential. Taken together, the GDI network can be a useful method to explain various dynamical behavior caused by complex gene-gene relations in GMI networks. A future study will include a more generalized analysis considering various mutation types, an examination of novel structural characteristics in the GDI network and an investigation on the dynamical influence among three or more genes based on multiple mutations.

## Additional file

Additional file 1: Figure S1. Pseudo-code for the Barabási-Albert model. It describes the algorithm to construct random network in Barabási-Albert model which is a type of network growth model. **Figure S2.** Pseudo-code for the shuffling model. It describes how to construct random network using shuffling model which rewires the edges of GMI network in a way that in-degree and out-degree of all nodes are preserved. **Figure S3.** Visualization of the GMI and the corresponding GDI networks in the case of ABAN. (**a**) The GMI network with ∣*V* ∣  = 44, ∣ *A* ∣  = 78. (**b**) The corresponding GDI network with |*V*| = 44 and |*A* ^′^| = 666. **Figure S4.** Visualization of the GMI and the corresponding GDI networks in the case of HSN. (**a**) The GMI network with ∣*V* ∣  = 1609, ∣ *A* ∣  = 5063. (**b**) The corresponding GDI network with |*V*| = 1609 and |*A* ^′^| = 21221. **Figure S5.** Relationship of the GDI value to the length of a shortest path in random networks. (**a**-**c**) Results of the random networks shuffled from AMRN, ABAN, and HSN, respectively. (**d**) Results of 250 BA random networks with ∣*V* ∣  = 50, ∣ *A* ∣  = 80. (**e**) Results of 250 BA random networks with ∣*V* ∣  = 50, ∣ *A* ∣  = 100. **Figure S6.** Relationship of the GDI value to the number of paths in random networks. (**a**-**c**) Results of the random networks shuffled from AMRN, ABAN, and HSN, respectively. (**d**) Results of 250 BA random networks with ∣*V* ∣  = 50, ∣ *A* ∣  = 80. (**e**) Results of 250 BA random networks with ∣*V* ∣  = 50, ∣ *A* ∣  = 100. **Figure S7.** Relationship of the GDI value to the number of feedback loops involving the gene pair. Relationship of the GDI value to the number of feedback loops involving the gene pair. (**a**-**c**) Results of AMRN, ABAN, and HSN, respectively. (**d**-**f**) Results of the random networks shuffled from AMRN, ABAN, and HSN, respectively. **Table S1.** AMRN dataset consisting of 10 nodes and 20 interactions after removing self-loops from the original dataset. It includes the information about gene name as source and target, and interaction type. **Table S2.** ABAN dataset consisting of 44 nodes and 78 interactions after removing self-loops from the original dataset. It consists the information about gene name as source and target, and interaction type. **Table S3.** HSN dataset consisting of 1609 nodes and 5063 interactions after removing self-loops from the original dataset. It consists the information about gene name as source and target, and interaction type**. Table S4.** Information of drug-targets and side-effects for genes in HSN. It includes information about drug-targets and side-effects genes in HSN. (PDF 4490 kb)

## Abbreviations

GDI: Gene-gene dynamics influence
GMI: Gene-gene molecular interaction
MIDI: Molecularly interacting and dynamically interacting
MIDN: Molecularly interacting but dynamically non-interacting
MNDI: Molecularly non-interacting but dynamically interacting
NCF: Nested canalyzing function
