# Supplementary material for: Construction and analysis of gene-gene dynamics influence networks based on a Boolean model
Source: BMC Syst Biol. 2017 Dec 21;11(Suppl 7):133. doi: 10.1186/s12918-017-0509-y (PMC5763298; doi:10.1186/s12918-017-0509-y)
Supplement: Additional file 1: Figure S1. — Pseudo-code for the Barabási-Albert model. It describes the algorithm to construct random network in Barabási-Albert model which is a type of network growth model. Figure S2. Pseudo-code for the shuffling model. It describes how to construct random network using shuffling model which rewires the edges of GMI network in a way that in-degree and out-degree of all nodes are preserved. Figure S3. Visualization of the GMI and the corresponding GDI networks in the case of ABAN. (a) The GMI network with ∣V ∣ = 44, ∣ A ∣ = 78. (b) The corresponding GDI network with |V| = 44 and |A ′| = 666. Figure S4. Visualization of the GMI and the corresponding GDI networks in the case of HSN. (a) The GMI network with ∣V ∣ = 1609, ∣ A ∣ = 5063. (b) The corresponding GDI network with |V| = 1609 and |A ′| = 21221. Figure S5. Relationship of the GDI value to the length of a shortest path in random networks. (a-c) Results of the random networks shuffled from AMRN, ABAN, and HSN, respectively. (d) Results of 250 BA random networks with ∣V ∣ = 50, ∣ A ∣ = 80. (e) Results of 250 BA random networks with ∣V ∣ = 50, ∣ A ∣ = 100. Figure S6. Relationship of the GDI value to the number of paths in random networks. (a-c) Results of the random networks shuffled from AMRN, ABAN, and HSN, respectively. (d) Results of 250 BA random networks with ∣V ∣ = 50, ∣ A ∣ = 80. (e) Results of 250 BA random networks with ∣V ∣ = 50, ∣ A ∣ = 100. Figure S7. Relationship of the GDI value to the number of feedback loops involving the gene pair. Relationship of the GDI value to the number of feedback loops involving the gene pair. (a-c) Results of AMRN, ABAN, and HSN, respectively. (d-f) Results of the random networks shuffled from AMRN, ABAN, and HSN, respectively. Table S1. AMRN dataset consisting of 10 nodes and 20 interactions after removing self-loops from the original dataset. It includes the information about gene name as source and target, and interaction type. Table S2. ABAN dataset consisting of 44 nodes and 78 [file 12918_2017_509_MOESM1_ESM.pdf]

Construction and analysis of gene-gene dynamics influence networks based on a Boolean model

Maulida Mazaya, Hung-Cuong Trinh, and Yung-Keun Kwon*

Department of Electrical/Electronic and Computer Engineering, University of Ulsan, 93 Daehak-ro, Nam-gu, Ulsan 44610, Republic of Korea.

* Corresponding author
E-mail: kwonyk@ulsan.ac.kr (YKK)

Supplementary Materials

```
function [V, A] = createRBN_BarabasiAlbert(N, e, d)
    //N:          The number of desirable nodes
    //e:          The number of initial nodes
    //d:          The number of interactions added at each step
    //V, A:       A set of nodes V and a set of links A of the resulting network generated by the Barabási-Albert model

    V←{0, 1, …, e-1};
    A←∅;
    for i:=0 to e-2
        for j:=i+1 to e-1
            if (randNumber(0,1) < 0.5)  // randNumber(0,1) returns a real number chosen from 0 to 1 uniformly at random
                A←A∪{(i, j)};
            else
                A←A∪{(j, i)};
            endif
        endfor
    endfor

    for i:= e to N-1
        for j:=0 to d-1
            do
                v = selection(V); //v is chosen with a probability proportional to its degree.
                if (randNumber(0,1) < 0.5)
                    v_Src← i, v_Dst← v;
                else
                    v_Src← v, v_Dst← i;
                endif
            until ((v_Src, v_Dst)∉ A);
            A←A∪{(v_Src, v_Dst)};
        endfor
        V←V∪{i};
    endfor
    return [V, A];
end
```

Figure S1. Pseudo-code for the Barabási-Albert model. It uses a preferential attachment scheme which is a type of network growth model as follows. The desirable number of nodes N, the number of nodes of a seed network e, and the number of interactions that should be added at each iteration d are given as parameters. A small seed network $G(V, A)$ is then created, where $V = \{v_1, v_2, \ldots, v_e\}$ and $A = \{(v_i, v_j) \mid i, j = 1,2, \ldots, e, i \neq j\}$, i.e., a complete network. At each iteration, a new node v is initially added to V. Then, d different interactions that individually connect v and $v' \in V \setminus \{v\}$ are newly added to A, where v' is determined with a probability proportional to the connectivity of v' (the connectivity of a node is defined as the number of interactions incident to the node and the direction of the new interaction is determined uniformly at random). This iteration process is repeated until $|V| = N$.

```
function [V, A] = createRBN_Shuffle (V_O, A_O, r)
    //V_O, A_O:        A set of nodes V_O and a set of links A_O of the reference network
    //r:               The intensity of shuffling
    //V, A:            A set of nodes V and a set of links A of the resulting network generated by the Shuffling model

    V ← V_O;
    A ← A_O;
    noIters ← r × | A_O |

    cnt ← 0

    while cnt < noIters
        (v_a, v_b) ← randSelectLink(A)
        (v_c, v_d) ← randSelectLink(A)
            // randSelectLink(A)  returns a link chosen from the set of links A uniformly at random

        if (v_a, v_d) ∉ A & (v_c, v_b) ∉ A
            A ← A \ { (v_a, v_b), (v_c, v_d) }  // remove two original links
            A ← A ∪ { (v_a, v_d), (v_c, v_b) }// insert two newly rewired links

            cnt ← cnt + 1
        endif

    endwhile

    return [V, A];
end
```

Figure S2. Pseudo-code for the shuffling model. It rewires the edges of a GMI network in a way that in- and out-degrees of all nodes are conserved. A reference network $G(V_0, A_0)$ and the shuffling intensity parameter are given. At every iteration, two randomly selected links, (v_a, v_b) and (v_c, v_d), are replaced with two new links, (v_a, v_d) and (v_c, v_b). This replacement conserves in-/out-degrees. This iteration process is repeated $noIters$ times.

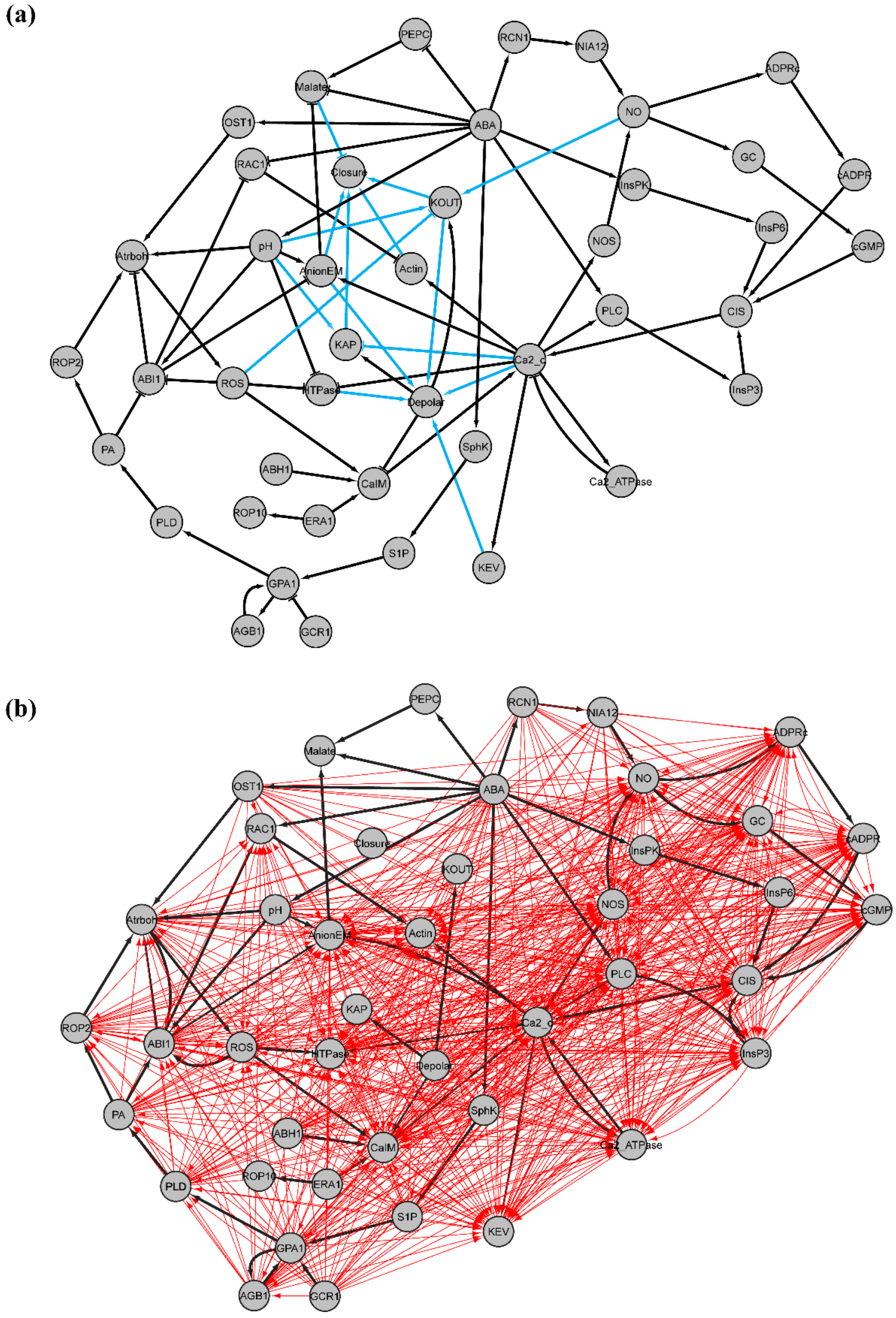

Figure S3. Visualization of the GMI and the corresponding GDI networks in the case of ABAN. **(a)** The GMI network with $|V| = 44, |A| = 78$. Arrow-headed and bar-headed lines indicate activating (positive) and inhibitory (negative) interactions, respectively. **(b)** The corresponding GDI network with $|V| = 44$ and $|A'| = 666$. The gene pairs belonging to MIDI, MNDI, and MIDN groups are represented by black, red, and blue colored links, respectively.

(a)

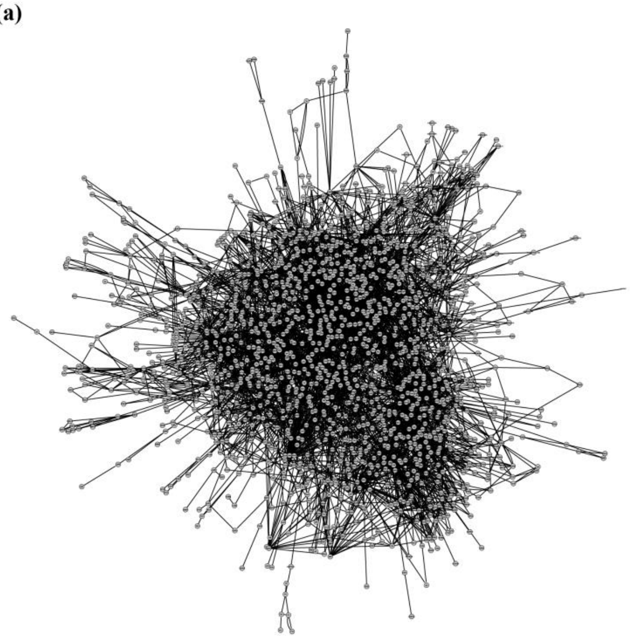

(b)

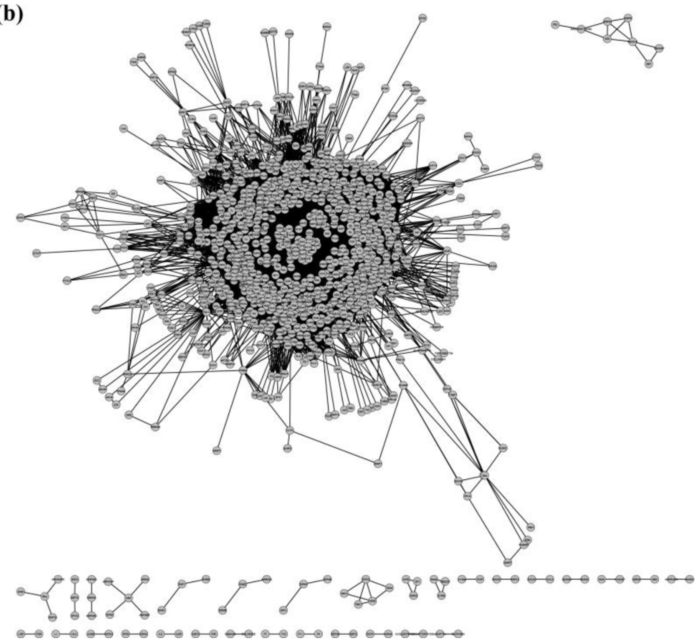

Figure S4. Visualization of the GMI and the corresponding GDI networks in the case of HSN. **(a)** The GMI network with $|V| = 1609, |A| = 5063$. Arrow-headed and bar-headed lines indicate activating (positive) and inhibitory (negative) interactions, respectively. **(b)** The corresponding GDI network with $|V| = 1609$ and $|A'| = 21221$. Considering the network size, we did not denote MIDI, MNDI, and MIDN edge groups by color.

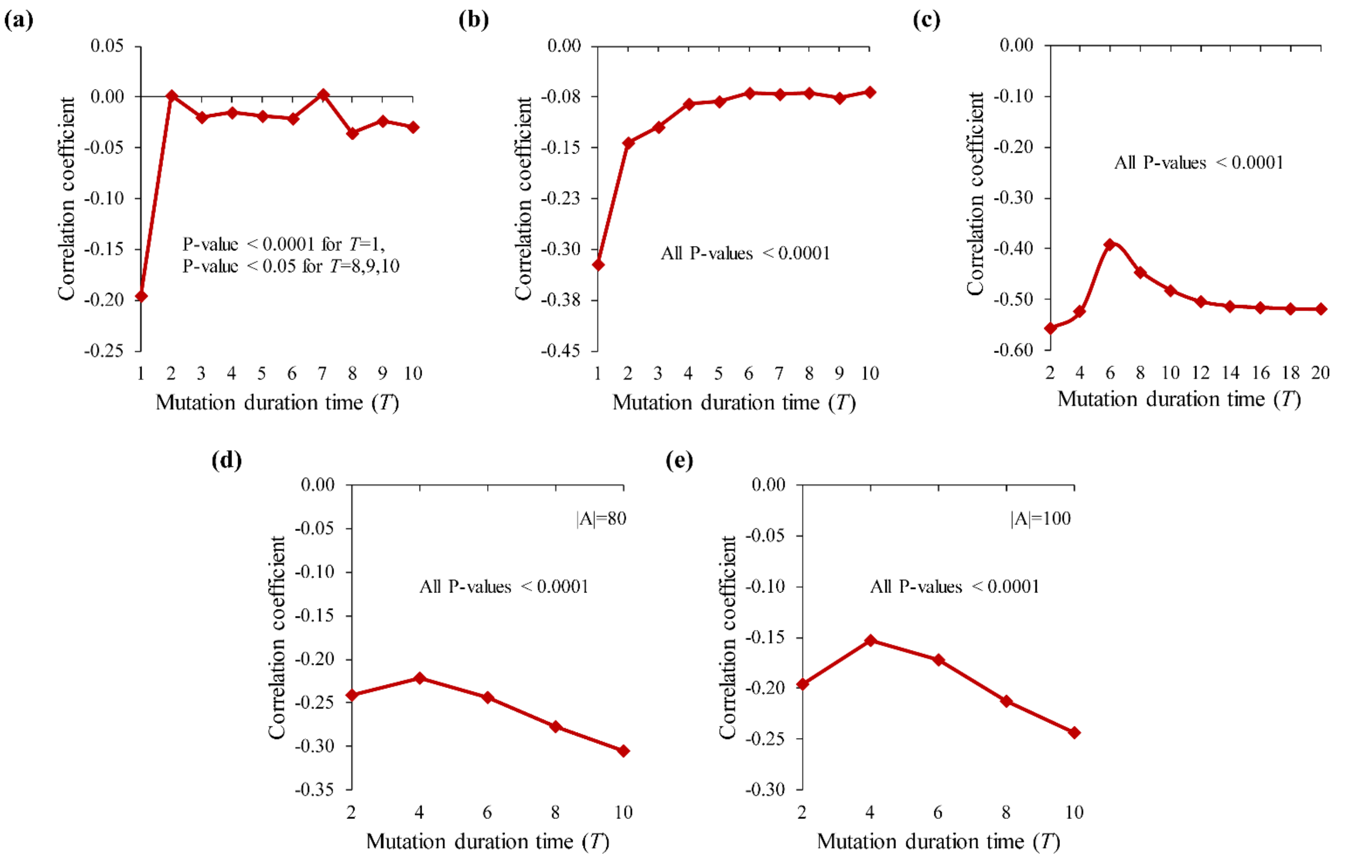

Figure S5. Relationship of the GDI value to the length of a shortest path in random networks. **(a)-(c)** Results of the random networks shuffled from AMRN, ABAN, and HSN, respectively. **(d)** Results of 250 BA random networks with $|V| = 50, |A| = 80$. **(e)** Results of 250 BA random networks with $|V| = 50, |A| = 100$. Y-axis values mean the correlation coefficients between $\mu(v_i, v_j)$ and $l(v_j, v_i)$ for all ordered pairs of genes (v_i, v_j). Significantly negative correlation coefficients were observed in most cases.

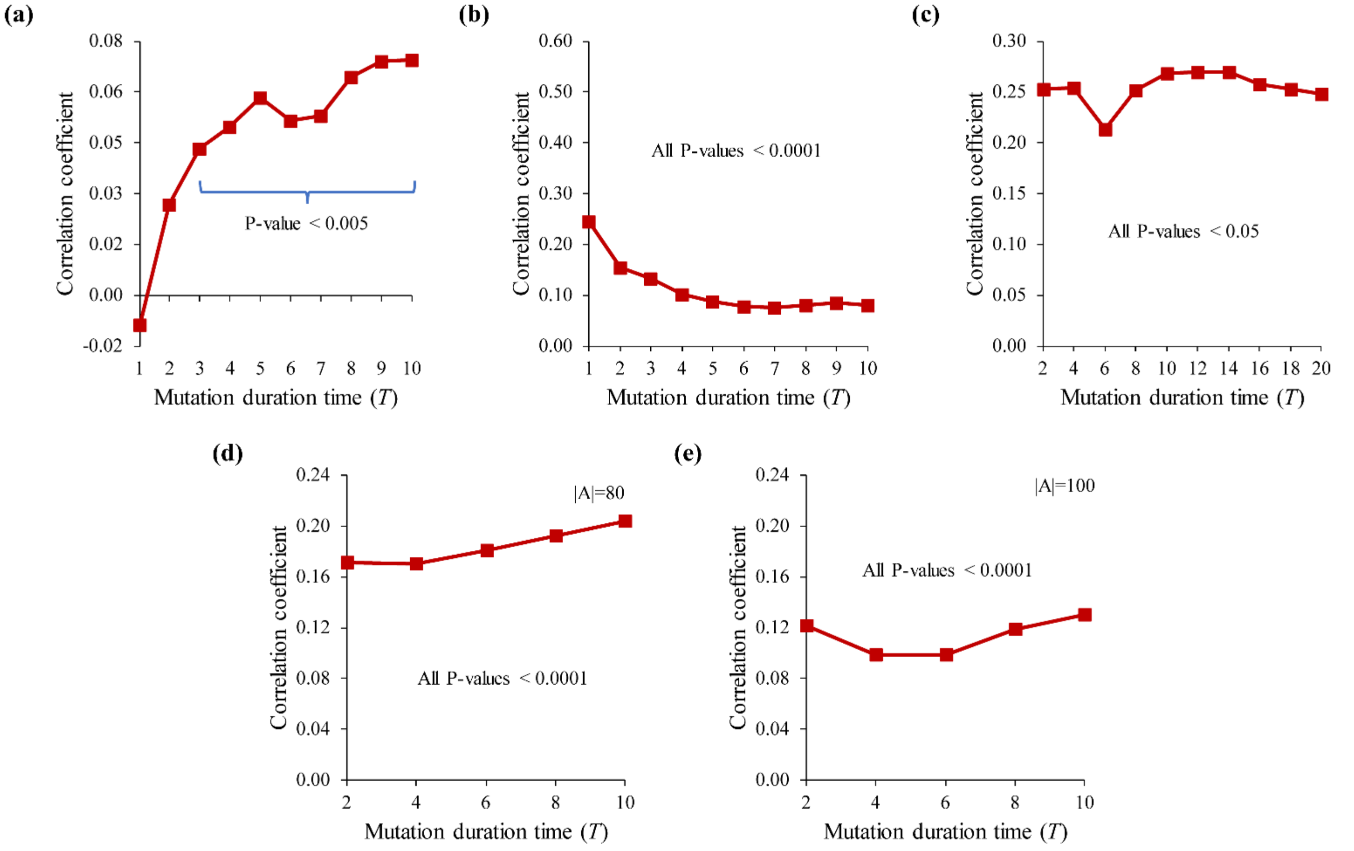

Figure S6. Relationship of the GDI value to the number of paths in random networks. **(a)-(c)** Results of the random networks shuffled from AMRN, ABAN, and HSN, respectively. **(d)** Results of 250 BA random networks with $|V| = 50, |A| = 80$. **(e)** Results of 250 BA random networks with $|V| = 50, |A| = 100$. Y-axis values mean the correlation coefficients between $\mu(v_i, v_j)$ and $n(v_i, v_j)$ for all ordered pairs of genes (v_i, v_j). Significantly positive correlation coefficients were observed in most cases.

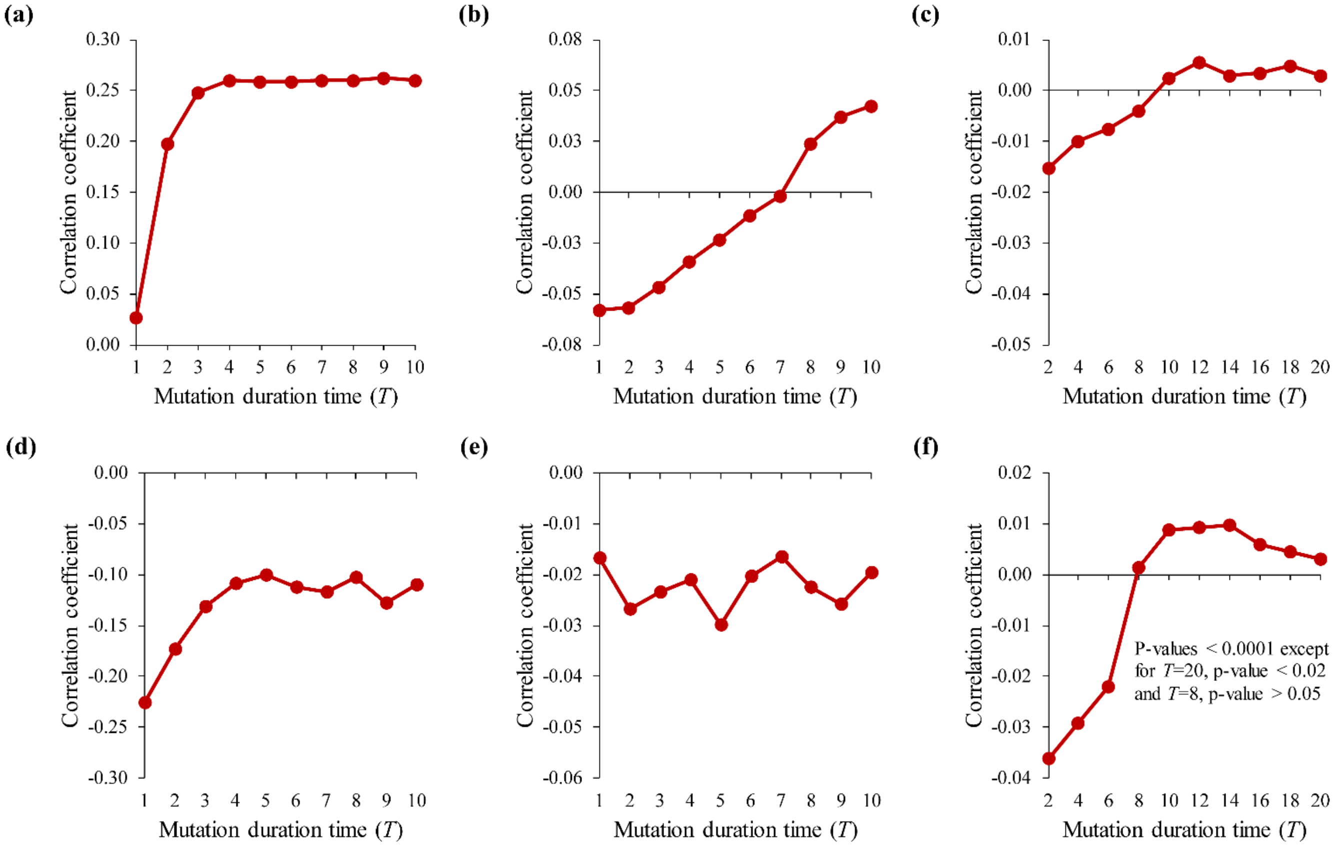

Figure S7. Relationship of the GDI value to the number of feedback loops involving the gene pair. **(a)-(c)** Results of AMRN, ABAN, and HSN, respectively. **(d)-(f)** Results of the random networks shuffled from AMRN, ABAN, and HSN, respectively. Y-axis values mean the correlation coefficients between $\mu(v_i, v_j)$ and $f(v_i, v_j)$ for all ordered pairs of genes (v_i, v_j). No consistent correlation coefficients were observed.

Table S1. AMRN dataset consisting of 10 nodes and 20 interactions after removing self-loops from the original dataset.

**Gene name (source)**	**Interaction**	**Gene name (target)**
EMF1	1	TFL1
EMF1	-1	AP1
EMF1	-1	LFY
AP1	-1	AG
AP1	1	LFY
TFL1	-1	AG
TFL1	-1	LFY
AG	-1	AP1
LFY	1	AP1
LFY	-1	TFL1
LFY	1	AG
LFY	1	PI
LFY	1	AP3
LUG	-1	AG
UFO	1	AP3
UFO	1	PI
SUP	-1	PI
SUP	-1	AP3
PI	1	AP3
AP3	1	PI

Table S2. ABAN dataset consisting of 44 nodes and 78 interactions after removing self-loops from the original dataset.

**Gene name (source)**	**Interaction**	**Gene name (target)**
pH	1	ABI1
PA	-1	ABI1
ROS	-1	ABI1
Ca2_c	1	Actin
RAC1	-1	Actin
NO	1	ADPRc
GPA1	1	AGB1
Ca2_c	1	AnionEM
pH	1	AnionEM
ABI1	-1	AnionEM
OST1	1	Atrboh
pH	1	Atrboh
ROP2	1	Atrboh
ABI1	-1	Atrboh
Ca2_c	1	Ca2_ATPase
CaIM	1	Ca2_c
CIS	1	Ca2_c
Ca2_ATPase	-1	Ca2_c
ADPRc	1	cADPR
ABH1	1	CaIM
ERA1	1	CaIM
ROS	1	CaIM
Depolar	-1	CaIM
GC	1	cGMP
cGMP	1	CIS
cADPR	1	CIS
InsP3	1	CIS
InsP6	1	CIS
KAP	1	Closure
Actin	1	Closure
AnionEM	1	Closure
KOUT	1	Closure
Malate	-1	Closure
AnionEM	1	Depolar
Ca2_c	1	Depolar
HTPase	1	Depolar
KEV	1	Depolar
KOUT	1	Depolar

NO	1	GC
AGB1	1	GPA1
S1P	1	GPA1
GCR1	-1	GPA1
Ca2_c	-1	HTPase
pH	-1	HTPase
ROS	-1	HTPase
PLC	1	InsP3
InsPK	1	InsP6
ABA	1	InsPK
Depolar	1	KAP
Ca2_c	-1	KAP
pH	1	KAP
Ca2_c	1	KEV
Depolar	1	KOUT
pH	1	KOUT
ROS	-1	KOUT
NO	1	KOUT
PEPC	1	Malate
AnionEM	-1	Malate
ABA	-1	Malate
RCN1	1	NIA12
NOS	1	NO
NIA12	1	NO
Ca2_c	1	NOS
ABA	1	OST1
PLD	1	PA
ABA	-1	PEPC
ABA	1	pH
ABA	1	PLC
Ca2_c	1	PLC
GPA1	1	PLD
ABA	-1	RAC1
ABI1	-1	RAC1
ABA	1	RCN1
ERA1	1	ROP10
PA	1	ROP2
Atrboh	1	ROS
SphK	1	S1P
ABA	1	SphK

Table S3. HSN dataset consisting of 1609 nodes and 5063 interactions after removing self-loops from the original dataset.

**Gene name (source)**	**Interaction**	**Gene name (target)**
plectin	0	ITGB4
plectin	0	GAB2
plectin	0	BPAG2
pGC	1	CGMP
p90RSK	1	MYT1
p90RSK	1	EEF2K
p90RSK	1	CREB
p90RSK	1	BAD
p90RSK	1	ATR
p90RSK	1	ATM
p90RSK	-1	BAD
p85beta	1	p110Ia
p85beta	0	Miz1
p85beta	0	KRT18
p85beta	0	KIT
p85beta	0	ITGB4
p85beta	0	ELK3
p85beta	0	CITED1
p85	1	p110Ia
p70s6K	1	S6
p70s6K	1	RPS6
p70s6K	1	PSMC4
p70s6K	1	GSK3B
p70s6K	1	CREM
p70s6K	-1	EEF2K
p70s6K	-1	BAD
p68	1	SRA1
p68	1	Era
p67phox	1	RAC1
p6	0	TLR2
p55gamma	1	p110Ia
p55gamma	0	KRT18
p55gamma	0	ITGB4
p53	1	Ub
p53	1	Puma
p53	1	P21
p53	1	Noxa
p53	1	MDM2
p53	1	GADD45

p53	1	FAS
p53	1	BNIP3L
p53	1	BIK
p53	1	BID
p53	1	BAX
p53	1	BAK
p53	1	ATM
p53	0	Ub
p53	0	Bok
p53	-1	POLR1B
p53	-1	p14ARF
p53	-1	CDK2
p53	-1	BCL2L1
p53	-1	BCL2
p38	1	STAT1
p38	1	SP1
p38	1	SMRT
p38	1	RB
p38	1	PRAK
p38	1	PLA2P
p38	1	MSK2
p38	1	MSK1
p38	1	MNK2
p38	1	MNK1
p38	1	MEK2
p38	1	MEK1
p38	1	MEF2B
p38	1	MEF2
p38	1	MAPKAP2
p38	1	MAPKAP-K3
p38	1	MAPK9
p38	1	ELK1
p38	1	CHOP10
p38	1	CEBPA
p38	1	CARD14
p38	1	c-JUN
p38	1	ATF2
p38	-1	P50
p38	-1	NFAT
p38	-1	Ca++
p32	1	Hrk
p300	1	p53
p300	1	NUR77

p300	1	MITF
p27	0	SKP2
p27	-1	CDK2
p190RhoGAP	1	RhoGDP
p190RhoGAP	-1	Rho
p16	-1	CDK4
p14ARF	1	RB
p14ARF	-1	PI3K
p14ARF	-1	MDM2
p14ARF	-1	E2F1
p120	1	STAT3
p120	0	SRC
p120	0	PTK
p120	0	PI3K
p110Ia	1	PI3P
p110Ia	0	KRT18
p110Ia	0	ITGB4
p110Ia	-1	p85
p107	1	DP1
p107	1	CCNE1
p107	0	P130
nPKC	1	RAC1
nPKC	1	IKBA
nPKC	-1	MEK1
mRNA	0	SAM68
mDIA	1	SRC
mDIA	1	IRSP53
mDIA	1	APAF1
hsp70	0	TID1
hsp70	0	JAK2
hsp70	0	IFN-yR
hsp70	0	Hsp90
hsp70	-1	APAF1
hsp70	-1	AIF
glucocorticoid	0	GR
dsRNA	1	PKR
dATP	1	APAF1
cPLA2	1	NOTCH4
cPLA2	1	NOTCH3
cPLA2	1	NOTCH2
cPLA2	1	NICD
cPLA2	1	JAG2
cPLA2	1	DLL1

cPLA2	1	AR
cPLA2	1	APP
cPLA2	0	PEN2
cPLA2	0	NOTCH3
cMAF	0	NFAT
cIAP2	1	BCL10
cIAP2	-1	cIAP
cIAP2	-1	CASP8
cIAP2	-1	CASP7
cIAP2	-1	CASP3
cIAP1	0	XIAP
cIAP1	-1	CASP7
cIAP1	-1	CASP3
cIAP	1	BIRC5
cIAP	0	XIAP
cIAP	0	TRAF2
cIAP	0	TRADD
cIAP	0	RIP
cIAP	0	cIAP1
cIAP	-1	XIAP
cIAP	-1	Livin
cIAP	-1	IAP
cIAP	-1	cIAP2
cAR	1	STAT
cAR	1	MEK1
cAR	1	Ca++
cAR	1	bARK
cAMPGEFII	1	RIM
cAMPGEFII	0	RIM
cADPR	1	RYR
c-Myc	1	SP1
c-Myc	1	p14ARF
c-Myc	0	PPARB
c-JUN	1	Hrk
c-JUN	1	CREB
c-JUN	1	BIM
c-JUN	0	TREH
c-JUN	0	JUND
c-JUN	0	FOS
c-JUN	0	ETS2
c-JUN	0	ETS1
c-JUN	0	ELK1
c-JUN	0	CREB

bRAF	1	MEK2
bRAF	1	MEK1
bRAF	1	MAP4K1
bARK	1	7TMR
bARK	-1	MOR
bARK	-1	M2R
bARK	-1	GAQ
bARK	-1	D2R
bARK	-1	B2AR
bARK	-1	AR
bARK	-1	AGTR1
a2-Antiplasmin	-1	PLASMIN
Zyxin	0	VASP
ZNF259	1	SENP1
ZEB1	1	CDH1
ZEB1	0	STXBP1
ZEB1	0	GAB2
ZEB1	0	CAVEOLIN
ZAP70	1	VAV2
ZAP70	1	TUBULIN
ZAP70	1	SLP-76
ZAP70	1	ECM
ZAP70	0	CD3
ZAK	1	MKK7
ZAK	1	JNKK1
YES	0	KIT
YES	0	ITGB4
XIAP	1	TAK1
XIAP	1	TAB1
XIAP	1	JNK
XIAP	1	CASP9
XIAP	1	BIRC5
XIAP	0	CHK1
XIAP	-1	MURR1
XIAP	-1	cIAP
XIAP	-1	CASP9
XIAP	-1	CASP7
XIAP	-1	CASP3
XDH	1	O2
XAP2	1	Hsp90
XAP2	1	AHR
XAP2	-1	APAF1
XAF1	-1	XIAP

WNT6	1	LRP
WNT6	1	FRIZZLED
WNT2	1	POFUT1
WNT	1	ITGB4
WNT	1	FRIZZLED
WNT	0	Ryk
WNT	0	PSMD4
WNT	0	PLSCR1
WNT	0	LRP5
WNT	0	ID2
WNT	0	FZD2
WNT	0	FRP
WNT	0	AR
WNT	-1	FRIZZLED
WIF1	-1	WNT
WAVE3	1	APAF1
WAVE2	1	APAF1
WAVE1	1	ARP23
WAVE1	1	APAF1
VRK1	1	ATF2
VPAC2R	1	CAMP
VPAC2R	0	VIP
VITRONECTIN	1	ITGB1
VIL2	0	ACTIN
VEGFR	1	SHC
VEGFR	1	PLCy
VEGFR	1	PI3K
VEGFR	1	FAK
VEGFR	1	AKT
VEGFR	-1	AKT
VEGF	1	HIF1A
VEGF	0	VEGFR
VDAC2	1	CYTOCHROMEC
VDAC2	1	BAX
VDAC2	1	BAK
VDAC2	-1	BAK
VCL	0	ACTIN
VAV2	1	RAC1
VAV2	1	CDC42
VAV2	1	ARHGAP1
VAV2	1	AKT
VAV2	0	SOCS1
VAV2	0	GAB2

VAV	1	RHOA
VAV	1	Rho
VAV	1	RAC1
VAV	1	AKT
VAV	0	SOCS1
VAV	0	SLP-76
VAV	0	GAB2
VASP	1	ACTIN
UbiL	0	BCLG
Ub	1	p27
Ub	1	E2
Ub	1	DELTA
Ub	0	ALPHACATENIN
Ub	-1	LRP6
Ub	-1	BAX
UXT	1	HBO1
UXT	1	HAT1
UBE3A	1	HBO1
UBE3A	1	HAT1
UBE2D3	1	SMAD2
UBE2D2	1	SMAD2
UBE2D1	1	SnoN
UBE1L	1	TRAF6
UBC9	1	UBE3A
UBC9	1	AR
UBC9	0	UBE3A
Ty	1	Tb
Ty	1	PDE6A
Tpl-2	1	MEK2
Tpl-2	1	MEK1
Tb	1	GDP
Tb	0	Ty
Ta	0	Ty
Ta	0	Tb
TYK2	1	STAT4
TYK2	1	STAT2
TYK2	1	STAT1
TWIST	-1	p14ARF
TUBULIN	1	DYNAMIN
TUBBY	1	GAQ
TSC2	-1	TSC1
TSC2	-1	RAP1
TSC1	-1	TOR

TSAP6	1	BNIP3L
TSA	-1	HDAC3
TRYPSIN	1	PAR2
TRKB	1	SHC
TRKB	1	IRS2
TRKB	1	IRS1
TRKB	1	FYN
TRIO	1	RAC1
TRAF6	1	TAK1
TRAF6	1	TAB2
TRAF6	1	TAB1
TRAF6	1	MEKK
TRAF6	1	MEK3
TRAF6	1	IKKA
TRAF6	1	ECSIT
TRAF3	0	TRAF6
TRAF2	1	TAK1
TRAF2	1	SENP1
TRAF2	1	p53
TRAF2	1	NFkB
TRAF2	1	MIG1
TRAF2	1	MEK1
TRAF2	1	LPL
TRAF2	1	IKBA
TRAF2	1	cIAP2
TRAF2	1	CEM15
TRAF2	1	ASK1
TRAF2	0	ZNF259
TRAF2	0	UBE2D1
TRAF2	0	TRAF3
TRAF2	0	DTX1
TRAF2	0	cIAP2
TRAF2	0	BPAG1
TRAF2	-1	MIG1
TRAF2	-1	ASK1
TRAF1	0	ZNF259
TRAF1	0	UBE2D1
TRAF1	0	TRAF2
TRADD	1	TRAF2
TRADD	1	RIP
TRADD	1	FADD
TRADD	0	TRAF6
TRADD	0	TRAF2

TRADD	0	DD
TOR	1	p70s6K
TOR	-1	EIF-4EBP
TNKS	1	PTEN
TNFR2	1	TRAF2
TNFR2	0	TRAF2
TNFR1	1	TRADD
TNFR1	1	JNKK1
TNFR1	0	TRAF2
TNFR1	0	TRADD
TNFB	0	TNFR2
TNFB	0	TNFR1
TNF	1	TNFR2
TNF	1	TNFR1
TNF	0	TNFR1
TLR9	0	TOLLIP
TLR7	0	TOLLIP
TLR4	0	TRAF6
TLR4	0	TOLLIP
TLR3	0	TOLLIP
TLR2	0	TLR6
TLR2	0	TLR1
TIRAP	0	TOLLIP
TIRAP	0	TLR6
TIRAP	0	TLR4
TIRAP	0	TLR2
TIRAP	0	TLR1
TIP1	0	VELI1
TIF2	1	AR
TIF2	0	TGIF
TIF2	0	SHARP
TIF2	0	MAGEA1
TIF2	0	CYTOCHROMEC
TIF2	0	CSL
TID1	1	JAK2
TID1	-1	IKK2
TIAM1	1	AKT
THRAP2	1	Era
THC	1	CB1R
THC	1	ARP2
TGFBR3	1	TGFB
TGFBR3	0	TGFB3
TGFBR3	0	TGFB2

TGFBR3	0	HSPG
TGFBR3	0	GIPC
TGFBR3	0	ARRB2
TGFBR2	1	TGFBR3
TGFBR2	1	TGFBR1
TGFBR2	1	TAB1
TGFBR2	1	ENG
TGFBR2	1	EIF3S2
TGFBR2	1	DAAM1
TGFBR2	0	ZFYVE9
TGFBR2	0	TGFBRAP1
TGFBR2	0	TGFB3
TGFBR2	0	TGFB2
TGFBR2	0	STRAP
TGFBR2	0	SNX6
TGFBR2	0	p85beta
TGFBR2	0	ID3
TGFBR2	0	EPS8
TGFBR2	0	EIF3S2
TGFBR2	0	DAB2
TGFBR2	0	CCNB2
TGFBR2	0	AP2B1
TGFBR2	0	ACVRL1
TGFBR1	1	TGFBR2
TGFBR1	1	TAK1
TGFBR1	1	SMAD3
TGFBR1	1	SMAD2
TGFBR1	1	PPP2R2A
TGFBR1	1	FNTA
TGFBR1	1	ENG
TGFBR1	1	CD44
TGFBR1	0	ZFYVE9
TGFBR1	0	TGFBRAP1
TGFBR1	0	TGFBR2
TGFBR1	0	TGFB2
TGFBR1	0	STRAP
TGFBR1	0	SNX6
TGFBR1	0	p85beta
TGFBR1	0	FKBP12
TGFBR1	0	EIF3S2
TGFBR1	0	DAB2
TGFBR1	0	CAVEOLIN
TGFBR1	0	AP2B1

TGFB	1	TGFBR1
TGFB	0	TGFBR3
TGFB	0	TGFBR2
TGFB	0	TGFBR1
TGFB	0	MIP1A
TGFB	0	HSPG
TGFB	0	ACVRL1
TFF2	1	ITGB1
TFF2	1	GH
TFF2	-1	APAF1
TFF1	1	ITGB1
TFF1	1	GH
TFF1	-1	APAF1
TERT	1	PTEN
TERT	0	TEP1
TERT	-1	PTEN
TEC	1	VAV
TEC	1	DOK
TEC	0	KIT
TCF7	1	SRF
TCF7	1	LEF1
TCF4	0	VAV3
TBX2	-1	p14ARF
TBK1	1	IRF3
TAX	0	VELI1
TAX	0	TRX
TAX	0	TIP1
TAU	0	TUBULIN
TANK	0	TRAF3
TANK	0	TRAF2
TANK	0	TRAF1
TANK	0	TNFR2
TAMALIN	1	SYK
TALIN	0	VCL
TALIN	0	ACTIN
TAK1	1	TAB1
TAK1	1	SMO
TAK1	1	NLK
TAK1	1	NIK
TAK1	1	MKK7
TAK1	1	MEK6
TAK1	1	MEK3
TAK1	1	MEK1

TAK1	1	JNKK1
TAK1	1	JNK
TAK1	1	HIPK2
TAK1	1	Akt
TAK1	0	CAMK
TAB2	1	TAK1
TAB2	0	TAK1
TAB1	1	TAK1
TAB1	1	p38
TAB1	1	NLK
TAB1	1	NIK
TAB1	1	MEK3
TAB1	0	TAK1
TAB1	0	TAB2
SnoN	0	FZR1
SnoN	0	CDC27
SnoN	0	CDC16
Sin3	1	Hypoxia
Sin3	0	UBE2D1
Sin3	0	TIF2
Sin3	0	TIEG2
Sin3	0	TGIF
Sin3	0	HERP1
Sara	1	TGFBR1
Sara	1	SMAD2
SYNTAXIN	1	KV11
SYNTAXIN	-1	NTYPECA
SYNTAPHILIN	-1	SYNTAXIN
SYNGAP	-1	RAS
SYNDECAN	0	SYNTENIN
SYNAPTOTAGMIN	1	SYNTAXIN
SYNAPTOTAGMIN	1	RTYPECA
SYNAPTOTAGMIN	1	LTYPECA
SYNAPTOBREVIN	0	SYNTAXIN
SYNAPTOBREVIN	0	SYNAPTOPHYSIN
SYNAPSIN	1	ACTIN
SYNAPSIN	-1	GALPHAZ
SYK	1	VAV
SYK	1	TUBULIN
SYK	1	SLP-76
SYK	1	SHC
SYK	1	PLCy
SYK	1	PLCg-2

SYK	1	LAT
SYK	1	BLNK
SYK	0	FCER1A
SV2A	1	SYNAPTOTAGMIN
SUMO1	1	CTBP1
SUMO1	0	TCF4
SUMO1	0	LEF
SUFU	1	ZEB1
SUFU	1	RKIP
SUFU	1	KIT
SUFU	1	ITGB4
SUFU	1	DVL2
SUFU	0	UBE3A
SUFU	0	UBE2D1
SUFU	0	STK36
SUFU	0	SAP18
SUFU	0	RALB
SUFU	0	PLD
SUFU	0	GAB2
SUFU	-1	SMO
SUFU	-1	CTNNB1
STK36	0	UBE3A
STK36	0	UBE2D1
STEP	1	HBO1
STEP	1	HAT1
STEP	0	KRT18
STEP	0	KIT
STEP	0	ITGB4
STEP	0	HBO1
STEP	-1	ERK2
STAT5B	1	MEK5
STAT5	0	STAT5B
STAT5	0	RIP140
STAT5	0	KIT
STAT5	0	GAB2
STAT3	1	STAT1
STAT3	1	ISRE
STAT3	1	GAS
STAT3	0	STAT5B
STAT3	0	PLSCR1
STAT3	0	plectin
STAT3	0	PIAS3
STAT3	0	N-WASP

STAT3	0	HES5
STAT3	0	HES1
STAT3	0	GRIM19
STAT3	0	AR
STAT3	0	ACK
STAT2	1	ISGF3G
STAT1	1	ISGF3G
STAT1	0	STAT5B
STAT1	0	STAT5
STAT1	0	STAT3
STAT1	0	PLSCR1
STAT1	0	plectin
STAT1	0	KIT
STAT1	0	GAB2
STAT	1	STAT1
STAT	1	P21
STAMBPL1	1	HAT1
SSTR2	1	SH3KBP1
SSTR2	1	GBETAGAMMA
SSTR2	1	GALPHAO
SSTR2	1	GALPHAI
SSTR1	1	GBETAGAMMA
SSTR1	1	GALPHAO
SSTR1	1	GALPHAI
SS	1	SSTR2
SS	1	SSTR1
SRPR	1	SEC61P
SRP54	1	SRPR
SRP54	1	NASCENTCHAIN
SRF	1	GATA4
SRF	0	TCF
SRF	0	SRE
SRE	0	TCF7
SRC	1	VAV2
SRC	1	TCF4
SRC	1	SYK
SRC	1	STAT3
SRC	1	SPECTRIN
SRC	1	Sin3
SRC	1	SHC
SRC	1	SH2D3C
SRC	1	SAM68
SRC	1	RHOA

SRC	1	RAC1
SRC	1	PXN
SRC	1	PTK
SRC	1	PLSCR1
SRC	1	PLCy
SRC	1	p190RhoGAP
SRC	1	P130Cas
SRC	1	NMDAR
SRC	1	MUSK
SRC	1	MEK1
SRC	1	LTYPECA
SRC	1	KIT
SRC	1	JAK1
SRC	1	HIPK2
SRC	1	GLYR
SRC	1	GIT1
SRC	1	GABAAR
SRC	1	GAB2
SRC	1	FAK
SRC	1	EPS8
SRC	1	EGFR
SRC	1	DYNAMIN
SRC	1	CBL
SRC	1	CAV2
SRC	1	C3G
SRC	1	bARK
SRC	1	ASAP1
SRC	1	Akt
SRC	0	VIL2
SRC	0	SYNAPSIN
SRC	0	SYK
SRC	0	STAT5
SRC	0	STAT3
SRC	0	STAT1
SRC	0	STAMBPL1
SRC	0	RACK
SRC	0	PSD95
SRC	0	GRIP1
SRC	0	DYNAMIN
SRC	0	DOK
SRC	0	AR
SRC	0	ACK
SRC	-1	CORTACTIN

SRC	-1	bARK
SRA1	1	p68
SRA1	1	Era
SPTAN1	0	VIL2
SPRY	1	PTPB1
SPRY	1	GAP
SPRY	-1	GRB2
SPRY	-1	EGF
SPRY	-1	CBL
SPINOPHILIN	1	PPP1CC
SPHK2	0	BCL2L1
SPHK1	1	S1P
SPAL	-1	RAP1
SP3	1	SP1
SP1	0	TIF2
SP1	0	SP3
SP1	0	DISHEVELED
SP1	0	AR
SOS2	1	P50
SOS2	-1	P50
SOS1	1	RAS
SOS1	1	RAC1
SOS1	1	GTP
SOS1	0	TCF4
SOS1	0	NCK2
SOS1	0	NCK
SOS1	0	Miz1
SOS1	0	GRB10
SOS1	0	GAB2
SOS1	0	EPS8
SOS1	0	E3B1
SORCIN	-1	RYR
SODIUM	1	GIRK
SODIUM	1	GAT1
SODD	1	TCF4
SODD	1	SAM68
SODD	1	KIT
SODD	1	ITGB4
SODD	1	DOK
SODD	1	DD
SODD	0	TRADD
SODD	0	TNFR1
SODD	0	STAT5

SODD	0	STAMBPL1
SODD	-1	TRADD
SODD	-1	TNFR1
SOD	1	O2
SOCS3	1	DVL3
SOCS	0	KIT
SOCS	-1	JAK2
SOCS	-1	JAK1
SOCS	-1	INSR
SNX6	1	UBE3A
SNX6	1	MBP
SNX4	0	SNX6
SNX2	0	SNX6
SNX1	0	SNX6
SNCA	0	Ub
SNCA	0	HBO1
SNAPIN	1	AC5
SNAPIN	-1	SNAP25
SNAP25	0	SYNTAXIN
SNAP25	0	SYNAPTOTAGMIN
SNAP25	0	SYNAPTOBREVIN
SNAP25	-1	NTYPECA
SNAI1	-1	CYP19A1
SNAI1	-1	CDH1
SMURF1	1	TGFBR1
SMURF1	1	SMAD7
SMRT	0	WNT2
SMRT	0	T3R
SMRT	0	SNW1
SMRT	0	PARD3
SMRT	0	NCOR
SMRT	0	CSL
SMRT	0	AR
SMRT	-1	PR-A
SMRT	-1	ERb
SMRT	-1	Era
SMPD2	1	SMPD1
SMPD1	1	MEKK
SMPD1	1	CYTOCHROMEC
SMPD1	1	BAX
SMO	1	TIF2
SMO	1	SP1
SMO	1	SMAD3

SMO	1	RALB
SMO	1	GLI3
SMO	1	GLI2
SMO	1	GLI1
SMO	1	DISHEVELED
SMO	1	BPAG2
SMO	0	UBE3A
SMO	0	PTCH2
SMO	0	PTCH
SMO	0	HBO1
SMO	0	HAT1
SMARCA4	1	Era
SMARCA4	1	CTNNB1
SMAD7	0	YAP1
SMAD7	0	TGFBR2
SMAD7	0	TGFBR1
SMAD7	0	TAK1
SMAD7	0	STRAP
SMAD7	0	STAMBPL1
SMAD7	0	SMURF2
SMAD7	0	SMURF1
SMAD7	0	SMO
SMAD7	0	RAP30
SMAD7	0	RANBP9
SMAD7	0	PARD3
SMAD7	0	ERBIN
SMAD7	0	DISHEVELED
SMAD7	-1	SMAD3
SMAD7	-1	SMAD2
SMAD6	0	TGFBR1
SMAD6	0	STRAP
SMAD6	0	AR
SMAD6	-1	SMAD1
SMAD5	1	ATF2
SMAD4	1	TOB
SMAD4	1	POLR2A
SMAD4	1	LEF
SMAD4	1	H3
SMAD4	1	EPS8
SMAD4	1	AR
SMAD4	0	ZFHX1B
SMAD4	0	TOB
SMAD4	0	TGFBRAP1

SMAD4	0	TCF4
SMAD4	0	TCF
SMAD4	0	SUMO1
SMAD4	0	STK11IP
SMAD4	0	STAMBPL1
SMAD4	0	SRC
SMAD4	0	SP1
SMAD4	0	SODD
SMAD4	0	SnoN
SMAD4	0	SNIP1
SMAD4	0	SMAD7
SMAD4	0	SKI
SMAD4	0	PRKAR1B
SMAD4	0	PPP2CA
SMAD4	0	PAX2
SMAD4	0	PARD3
SMAD4	0	NUP214
SMAD4	0	NOTCH3
SMAD4	0	NICD
SMAD4	0	MAML1
SMAD4	0	LEF
SMAD4	0	HOXA9
SMAD4	0	GRIP1
SMAD4	0	GAB2
SMAD4	0	FORKHEAD
SMAD4	0	ERBIN
SMAD4	0	DTX1
SMAD4	0	DISHEVELED
SMAD4	0	DCP1A
SMAD4	0	CTCF
SMAD4	0	CRI2
SMAD4	0	COPS5
SMAD4	0	CITED1
SMAD4	0	CD44
SMAD4	0	BPAG2
SMAD4	0	ASCL1
SMAD4	0	ARNIP
SMAD4	-1	CDC25A
SMAD3	1	SMAD4
SMAD3	1	MITR
SMAD3	0	ZFYVE9
SMAD3	0	ZFHX1B
SMAD3	0	ZEB1

SMAD3	0	VDR
SMAD3	0	TIF2
SMAD3	0	TGFBR1
SMAD3	0	TCF
SMAD3	0	STRAP
SMAD3	0	SP1
SMAD3	0	SODD
SMAD3	0	SNW1
SMAD3	0	SnoN
SMAD3	0	SMURF1
SMAD3	0	SMAD4
SMAD3	0	SKI
SMAD3	0	SHIP2
SMAD3	0	SAP97
SMAD3	0	PRKAR1B
SMAD3	0	PPP2CA
SMAD3	0	PCAF
SMAD3	0	PAX2
SMAD3	0	PARD3
SMAD3	0	NUP214
SMAD3	0	NUP153
SMAD3	0	NICD
SMAD3	0	LEF
SMAD3	0	KPNB1
SMAD3	0	JAG1
SMAD3	0	HGS
SMAD3	0	GRIP1
SMAD3	0	FZD2
SMAD3	0	FOXH1
SMAD3	0	FORKHEAD
SMAD3	0	ERBIN
SMAD3	0	EPS8
SMAD3	0	EF1A
SMAD3	0	E2F5
SMAD3	0	DISHEVELED
SMAD3	0	DAB2
SMAD3	0	CTCF
SMAD3	0	CRI2
SMAD3	0	CDC27
SMAD3	0	CDC16
SMAD3	0	CD44
SMAD3	0	CAMK2A
SMAD3	0	BPAG2

SMAD3	0	AXIN2
SMAD3	0	ATF3
SMAD3	0	ARNIP
SMAD3	0	AR
SMAD3	0	AML3
SMAD2	0	ZFYVE9
SMAD2	0	ZFHX1B
SMAD2	0	ZEB1
SMAD2	0	UBE2D1
SMAD2	0	TIF2
SMAD2	0	TGIF
SMAD2	0	TGFBR1
SMAD2	0	STRAP
SMAD2	0	STAMBPL1
SMAD2	0	SP1
SMAD2	0	SNW1
SMAD2	0	SnoN
SMAD2	0	SNIP1
SMAD2	0	SMURF1
SMAD2	0	SMAD4
SMAD2	0	SMAD3
SMAD2	0	SKI
SMAD2	0	SAP97
SMAD2	0	RALB
SMAD2	0	PCAF
SMAD2	0	PAX2
SMAD2	0	PARD3
SMAD2	0	NUP214
SMAD2	0	NUP153
SMAD2	0	LEF
SMAD2	0	HGS
SMAD2	0	GRIP1
SMAD2	0	FOXH1
SMAD2	0	ERBIN
SMAD2	0	EPS8
SMAD2	0	EF1A
SMAD2	0	DISHEVELED
SMAD2	0	DAB2
SMAD2	0	CRI2
SMAD2	0	CDC27
SMAD2	0	CDC16
SMAD2	0	CAMK2A
SMAD2	0	AML3

SMAD1	0	PCAF
SMAD1	0	NOTCH3
SMAD1	0	NICD
SMAD1	0	DAB2
SKP2	0	Ub
SKP2	0	TCF3
SKP1	1	TCF3
SKP1	1	SMAD3
SKP1	1	NOTCH4
SKP1	1	NICD
SKP1	1	EPS8
SKP1	0	SKP2
SKP1	0	Idax
SKP1	0	CD44
SKIP	-1	PKC
SKI	0	UBE3A
SIVA1	-1	BCL2L1
SIAH	-1	VAV2
SHP2	1	PXN
SHP2	1	PI3K
SHP2	1	MEK1
SHP2	1	GRB2
SHP2	1	GAB2
SHP2	0	TIF2
SHP2	0	Miz1
SHP2	0	KRT18
SHP2	0	KIT
SHP2	0	FZD2
SHP2	0	E2F5
SHP2	-1	SPRY
SHP2	-1	PXN
SHP2	-1	GAB1
SHP1	1	STAT5
SHP1	1	SHC
SHP1	1	KIT
SHP1	1	CTNND1
SHP1	1	AR
SHP1	0	VAV3
SHP1	0	TGFBR2
SHP1	0	TGFBR1
SHP1	0	SHP2
SHP1	0	RNTRE
SHP1	0	NICD

SHP1	0	Miz1
SHP1	0	KRT18
SHP1	0	ITGB4
SHP1	0	GRB10
SHP1	0	GAB2
SHP1	0	ELK3
SHP1	0	DOK
SHP1	0	CITED1
SHP1	0	CBLB
SHP1	-1	VAV
SHP1	-1	STAT6
SHP1	-1	SLP-76
SHP1	-1	JAK2
SHP1	-1	JAK1
SHP	-1	Era
SHOC2	0	UBC9
SHIP	0	KIT
SHH	0	PTCH2
SHH	0	PTCH
SHH	0	HHIP
SHH	0	GAS1
SHC	1	SOS1
SHC	1	SHIP
SHC	1	RAC1
SHC	1	PI3K
SHC	1	GRB2
SHC	1	ERK1
SHC	0	TEC
SHC	0	SRC
SHC	0	SOS2
SHC	0	SOS1
SHC	0	SHP1
SHC	0	SHIP2
SHC	0	RET
SHC	0	RAP74
SHC	0	PLSCR1
SHC	0	p85beta
SHC	0	Miz1
SHC	0	ITGB4
SHC	0	GRIT
SHC	0	GAB2
SHC	0	EPS8
SHC	0	DOK

SHC	0	CITED1
SHC	0	CEACAM1
SHC	0	AP2A1
SHARP	1	NCOR
SHARP	1	GRIP1
SHARP	-1	SRA1
SHANK	1	P50
SH3KBP1	1	JAK2
SH3KBP1	0	KIT
SH3KBP1	0	CITED1
SH3KBP1	0	ASAP1
SH3KBP1	-1	SPRY
SH3KBP1	-1	RET
SH3KBP1	-1	ALPHAACTININ
SF1	1	TCF4
SF1	1	MYB
SF1	1	LEF
SERPINC1	-1	F3
SERPINC1	-1	F2
SEROTONIN	1	FIVEHT4R
SEROTONIN	1	FIVEHT2AR
SEROTONIN	1	FIVEHT1CR
SEROTONIN	1	FIVEHT1AR
SENP1	1	TIF2
SENP1	0	ZNF259
SEC63P	1	SEC61P
SEC63P	1	BIP
SDF1	1	Gy
SDF1	1	GAQ
SDF1	1	GA
SCF	0	KIT
SAPAP	0	SSCAM
SAP97	0	SAP30
SAP97	0	SAP18
SAP97	0	PLSCR1
SAP97	0	FZD7
SAP97	0	FZD4
SAP97	-1	KAR
SAP18	0	UBE2D1
SAP18	0	SAP30
SAM68	0	TCF4
SAM68	0	NICD
SAM68	-1	CBP

SAG	1	Rho
SAC	1	ATP
S60	1	SRP54
S60	0	SEC61P
S6	1	PSMC4
Ryk	1	FRIZZLED
Ryk	1	DSH
RhoGDP	1	RhoGTP
Rho	1	ROCK1
Rho	1	mDIA
Rho	1	ACTIN
RasGRP	1	RAC1
RYR	1	Ca++
RXR	0	SMRT
RUVBL1	0	TBP
RTYPECA	1	Ca++
RTKN	0	WNT7A
RSK2	1	HIST3H3
RSK2	1	FOS
RSK2	1	CREB
RSK2	0	HBO1
RSK2	0	HAT1
RSK2	-1	BAD
RSK	1	S6
RSK	1	ROC1
RSK	1	CREB
RSK	0	HBO1
RSK	0	HAT1
RSK	-1	EEF2K
RSK	-1	BAD
RPTK	1	SHC
RPTK	1	PLCy
RPTK	1	p85
RPTK	1	GRB2
RPTK	1	GAB
ROS	-1	FKHRL1
ROR2	0	WNT5A
ROCK2	1	PROFILIN
ROCK2	1	MRLC
ROCK2	1	LIMK1
ROCK2	1	CRMP2
ROCK2	1	CPI17
ROCK2	-1	MLCP

ROCK1	1	myosin
ROCK1	1	MRLC
ROCK1	1	MLC
ROCK1	1	LIMK1
ROCK1	1	GATA1
ROCK1	1	CTBP2
ROCK1	-1	MLCP
ROCK1	-1	MLCK
ROC1	1	SMAD3
ROC1	0	SMAD4
ROC1	0	AR
RNTRE	1	NOTCH2
RNTRE	1	MAP1B
RNTRE	1	EPS8
RNTRE	1	EIF3S2
RNTRE	1	CAMK2A
RNTRE	1	ATF1
RNTRE	1	ARNIP
RKIP	0	UBE3A
RKIP	0	UBC9
RIP140	-1	Era
RIP	1	Ser-prot
RIP	1	NIK
RIP	0	TRAF6
RIP	0	TRAF2
RIP	0	TRAF1
RIP	0	TRADD
RIP	0	TNFR2
RIP	0	TNFR1
RIP	0	MEKK3
RIP	-1	PARP
RIN1	-1	RAS
RIM	1	SYNAPTOTAGMIN
RIM	1	SNAP25
RIM	1	RABPHILIN
RIM	1	RAB3
RIM	1	P1433
RIM	1	MUNC13
RIIa	0	RIIb
RHOGEFs	1	RHOA
RHOGEFs	1	PKN
RHOGEFs	-1	PKN
RHOGEFs	-1	GNA13

RHOA	1	ROCK2
RHOA	1	PXN
RHOA	1	PLD
RHOA	1	PKC
RHOA	1	mDIA
RHOA	1	JNK
RHOA	1	FAK
RHOA	1	ACTIN
RHOA	-1	IKBA
RHOA	-1	GDP
RHEB	1	RAF1
RGS4	-1	GAQ
RGS2	-1	Gia
RGS2	-1	GAQ
RGS2	-1	GALPHAI
RGS	-1	Gas
RGS	-1	GAQ
RGS	-1	Gai
RET	1	SHC
RET	1	GRB2
REPS2	0	UBE2D3
REPS1	0	UBE2D3
REPS1	0	Miz1
RELN	1	VLDLR
RELN	1	APOER2
RELN	-1	ITGB1
RELA	1	IL8
REA	-1	Era
RB	1	NFYA
RB	1	DP1
RB	1	CCNE1
RB	1	CCNB1
RB	1	CCNA2
RB	-1	POLR1B
RB	-1	E2F1
RASGRP3	1	RAS
RASGRP3	1	RAP1
RASGRP1	1	RAS
RASGRF	1	RAS
RASGRF	1	RAC1
RASGRF	1	AKT
RASGRF	0	NMDAR
RASGAP	1	AKT

RASGAP	0	SOCS3
RASGAP	0	SAM68
RASGAP	0	KIT
RASGAP	0	HD
RASGAP	0	GAB2
RASGAP	0	CAV2
RASGAP	-1	RAS
RASGAP	-1	RAC1
RAS	1	RALGDS
RAS	1	RAF1
RAS	1	PI3K
RAS	1	MLK3
RAS	1	bRAF
RAS	0	UBE3A
RAS	0	UBC9
RAS	0	SHOC2
RAS	0	RAP74
RAR	0	RXR
RAR	-1	CTNNB1
RAP74	1	WNT2
RAP74	1	RANBP9
RAP74	1	POLR2A
RAP74	0	WNT2
RAP74	0	UBE3A
RAP30	1	SMO
RAP30	1	PTP-SL
RAP2	1	RAS
RAP2	1	RALGDS
RAP1GAP	-1	RAP1A
RAP1GAP	-1	RAP1
RAP1B	1	Rho
RAP1B	1	RAC1
RAP1B	1	ERK1
RAP1A	1	bRAF
RAP1	1	RALGDS
RAP1	1	bRAF
RAP1	-1	RAF1
RANBP9	1	SMO
RANBP2	1	RAN
RANBP2	0	RanGAP
RANBP1	0	RanGAP
RANBP1	0	RANBP2
RALGDS	1	RAL

RALBP1	1	RAC1
RALBP1	1	CDC42
RALBP1	0	REPS1
RALBP1	0	ARIP2
RALBP1	-1	CDC42
RALBP1	-1	AKT
RALB	0	UBE2D3
RALB	0	STK36
RALB	0	SKI
RAL	1	RALBP1
RAIDD	1	CASP2
RAIDD	0	TRAF2
RAIDD	0	TRAF1
RAIDD	0	RIP
RAF1	1	MEK2
RAF1	1	MEK1
RAF1	1	MAP4K1
RAF1	-1	BAD
RAD9	-1	BCL2L1
RAD9	-1	BCL2
RAD51	1	BRCA2
RAD17	0	RAD9
RAD1	0	RAD9
RAD1	0	RAD17
RACK	-1	FYN
RAC1	1	VIL2
RAC1	1	SRC
RAC1	1	RALGDS
RAC1	1	RAF1
RAC1	1	PI3K
RAC1	1	PAK1
RAC1	1	p67phox
RAC1	1	p38
RAC1	1	p110Ia
RAC1	1	NFkB
RAC1	1	NAP
RAC1	1	MLK1
RAC1	1	MEKK
RAC1	1	GDP
RAC1	1	ERK2
RAC1	1	ERK1
RAC1	1	bRAF
RAC1	1	APAF1

RAC1	1	A-Raf
RAC1	0	SOS1
RABPHILIN	1	RAB3
RABGDI	-1	RAB3
RAB5	0	RNTRE
Pygo	1	Lgs
Puma	1	BAX
Puma	-1	MCL1
Puma	-1	BCL2L1
Puma	-1	BCL2
Puma	-1	Bcl-w
PrP	-1	BAX
PYGB	1	Glycogen
PXN	1	TALIN
PXN	1	P130Cas
PXN	1	ILK
PXN	1	FYN
PXN	1	CRK
PXN	0	VCL
PXN	0	TALIN
PXN	0	SPTAN1
PXN	0	AR
PTPN12	1	PLSCR1
PTPA	1	SRC
PTPA	1	LCK
PTPA	1	FYN
PTP1B	-1	INSR
PTP-SL	1	HCK
PTP-SL	1	HBO1
PTP-SL	1	HAT1
PTP	-1	TYK2
PTP	-1	STAT1
PTP	-1	JAK1
PTMA	0	CYTOCHROMEC
PTMA	-1	PP32
PTK	1	TSC1
PTK	1	SRC
PTK	1	SHC
PTK	1	RAC1
PTK	1	p38
PTK	1	MEK3
PTK	1	MEF2
PTK	1	JNK

PTK	1	GRB2
PTK	1	FKHRL1
PTK	1	EGFR
PTK	0	SYK
PTK	0	SRC
PTK	0	PXN
PTK	-1	KV12
PTEN	1	PIB5PA
PTEN	1	INPP5A
PTEN	-1	PIP3
PTEN	-1	PI3K
PTEN	-1	FAK
PTCH2	1	KIT
PTCH2	1	DOK
PTC	1	CDK1
PTC	0	SHH
PSMD4	0	TCF3
PSMC4	0	SEC61P
PSMC4	0	S60
PSMC4	0	mRNA
PSMC4	0	EIF3S1
PSMC4	0	EIF1A
PSEN1	1	Ub
PSEN1	0	TAU
PSEN1	0	SORCIN
PSEN1	-1	CTNNB1
PSD95	1	MAP1A
PSD95	0	YES
PSD95	0	TAMALIN
PSD95	0	SYNGAP
PSD95	0	STARGAZIN
PSD95	0	SPAL
PSD95	0	PLSCR1
PSD95	0	FZD7
PSD95	0	FZD4
PSD95	0	FZD2
PROKR1	-1	EIF2A
PROFILIN	1	PIP2
PROFILIN	1	ACTIN
PROFILIN	0	VASP
PROFILIN	0	SYNAPSIN
PRMT1	1	SAM68
PRMT1	0	AR

PRKCG	1	RKIP
PRKCG	1	DVL2
PRKCG	1	DAB2
PRKCD	1	ITGB4
PRKCD	1	ITGA6
PRKCD	1	DAB2
PRF1	1	CDK2
PRAK	1	HSP27
PR-A	-1	Era
PQCaCh	1	Ca++
PQCaCh	0	SYNTAXIN
PQCaCh	0	SYNAPTOTAGMIN
PQCaCh	0	SNAP25
PPP3R2	1	NOS1
PPP3R2	1	NFAT
PPP3R2	1	CABIN1
PPP3R2	0	NFAT
PPP3R2	0	CAM
PPP2R5C	1	LTYPECA
PPP2R5C	1	GSK3A
PPP2R5C	1	COFILIN1
PPP2R5C	1	BAD
PPP2R5C	-1	P130Cas
PPP2R5C	-1	NMDAR
PPP2R5C	-1	NFH
PPP2R5C	-1	CREB
PPP2R5C	-1	CAMKIV
PPP2R5C	-1	CAMK1
PPP2R5C	-1	BCL2
PPP2R5C	-1	AKT
PPP2CA	1	CAMK2A
PPP2CA	1	ARNIP
PPP1CC	1	COFILIN1
PPP1CC	1	BCL2
PPP1CC	0	YOTIAO
PPP1CC	-1	NMDAR
PPP1CC	-1	CREB
PPP1CC	-1	CAMK2A
PPARG	0	TRAF2
PPARG	0	TIF2
PPARG	0	RXR
PPARA	1	SMRT
PPARA	1	MAPK9

PPARA	1	Hsp90
PPARA	-1	SRC
PPARA	-1	IKBA
PP5	-1	TAU
PP32	1	CASP3
PP32	1	APAF1
PP2C	1	BAD
PP2C	-1	AXIN
PP2B	1	DARPP-32
PP2A	1	TAU
PP2A	1	PTEN
PP2A	1	MKK7
PP2A	1	JNKK1
PP2A	1	EBP
PP2A	1	DARPP-32
PP2A	1	CHREBP
PP2A	1	BETAARRESTIN
PP2A	1	BAD
PP2A	1	AXIN
PP2A	1	AKT
PP2A	0	APC
PP2A	-1	AXIN
PP1	1	BAD
POR1	1	APAF1
POLR2A	0	RAP74
POFUT1	1	SMAD3
POFUT1	1	PPP2CA
POFUT1	1	PAX2
POFUT1	1	NICD
POFUT1	1	JAG1
POFUT1	1	GRB10
POFUT1	1	DLL1
POFUT1	1	DISHEVELED
POFUT1	1	CAV2
POFUT1	0	WNT2
PMCA	-1	Ca++
PLSCR1	0	WNT3
PLSCR1	0	WNT2
PLSCR1	0	PTPN12
PLSCR1	0	PSMD4
PLK3	1	p53
PLK3	1	CDC25C
PLK1	-1	MYT1

PLG	1	PLASMIN
PLD	0	TCF4
PLD	0	STXBP1
PLD	0	GAB2
PLCy	1	PKC
PLCy	1	PIP2
PLCy	1	PIB5PA
PLCy	1	PI3K
PLCy	1	IP3
PLCy	1	EIF4A
PLCy	1	DAG
PLCy	1	CALM3
PLCy	1	Ca++
PLCy	0	SYNAPSIN
PLCy	0	SAM68
PLCy	-1	PIP2
PLCg-2	1	DAG
PLCb	1	PIB5PA
PLCb	1	IP3
PLCb	1	DAG
PLCb	1	CALM3
PLCb	0	TUBBY
PLCb	-1	PIP2
PLC	1	PIP2
PLC	1	DAG
PLC	-1	PIP2
PLASMIN	1	Fibrinogen
PLA2P	1	AA
PKR	1	TLR4
PKR	1	EIF2A
PKR	0	TIRAP
PKR	-1	EIF2A
PKN	1	CPI17
PKI	-1	PKA
PKG	1	PDE5A
PKG	1	NOS1
PKG	1	MGLUR7
PKG	1	CREB
PKG	1	ADPRIBSYCYCLASE
PKD	1	P1433
PKD	1	GAB2
PKCz	1	RKIP
PKCz	1	p70s6K

PKCE	0	ACTIN
PKCA	1	RASGRP3
PKCA	1	RASGRP1
PKCA	1	RAF1
PKCA	1	PTEN
PKCA	1	PLA2P
PKCA	1	NTYPECA
PKCA	1	MGLUR7
PKCA	1	MGLUR1
PKCA	1	MBP
PKCA	1	MAP2
PKCA	1	KAR
PKCA	1	GAP43
PKCA	1	EIF4E
PKCA	1	CPI17
PKCA	1	AC5
PKCA	1	AC2
PKCA	1	ABL1
PKCA	0	TIAM1
PKCA	0	RACK
PKCA	0	PICK1
PKCA	0	AKAP
PKCA	-1	SNAP25
PKCA	-1	PMCA
PKCA	-1	PLCb
PKCA	-1	NOS1
PKCA	-1	NMDAR
PKCA	-1	MUNC18
PKCA	-1	MARCKS
PKCA	-1	LTYPECA
PKCA	-1	IRS1
PKCA	-1	GSK3A
PKCA	-1	GLYR
PKCA	-1	GABAAR
PKCA	-1	FASCIN
PKCA	-1	DYNAMIN
PKCA	-1	DGK
PKCA	-1	BCL2
PKCA	-1	bARK
PKCA	-1	AMPAR
PKCA	-1	ADDUCIN
PKC	1	SPHK1
PKC	1	SLC9A1

PKC	1	S1P
PKC	1	RasGRP
PKC	1	RAF1
PKC	1	RAD9
PKC	1	RAC1
PKC	1	PTK
PKC	1	PSMC4
PKC	1	PPP3R2
PKC	1	PLD
PKC	1	PKD
PKC	1	p70s6K
PKC	1	NOS1
PKC	1	NFkB
PKC	1	MARCKS
PKC	1	ITGA1
PKC	1	GDP
PKC	1	ELK1
PKC	1	EIF4F
PKC	1	DAG
PKC	1	CPI
PKC	1	CABIN1
PKC	1	Ca++
PKC	1	BCL2
PKC	-1	GSK3A
PKC	-1	EIF4F
PKC	-1	EIF4E
PKC	-1	EGFR
PKAc	1	PP2A
PKAc	1	P21
PKAc	1	DARPP-32
PKAc	1	CREM
PKAc	1	CREB
PKAc	1	CHREBP
PKAc	1	BAD
PKAc	0	SAC
PKAc	0	RIIb
PKAc	0	RIIa
PKAc	-1	SMO
PKAc	-1	NFAT
PKAc	-1	Calpain2
PKA	1	TAU
PKA	1	SRC
PKA	1	SPINOPHILIN

PKA	1	SNAPIN
PKA	1	RIM
PKA	1	RAP1A
PKA	1	RAF1
PKA	1	RABPHILIN
PKA	1	PHKA2
PKA	1	PDE5A
PKA	1	PDE4D
PKA	1	PDE3B
PKA	1	PDE3A
PKA	1	PDE
PKA	1	p38
PKA	1	NMDAR
PKA	1	NFkB
PKA	1	MST3
PKA	1	MGLUR7
PKA	1	MAP2
PKA	1	KAR
PKA	1	GLYR
PKA	1	FILAMIN
PKA	1	DARPP-32
PKA	1	CREB
PKA	1	CBP
PKA	1	bRAF
PKA	1	ATF1
PKA	1	AMPAR
PKA	0	YOTIAO
PKA	-1	SYNAPSIN
PKA	-1	STEP
PKA	-1	RAP1
PKA	-1	RAF1
PKA	-1	PQCaCh
PKA	-1	PMCA
PKA	-1	PLCy
PKA	-1	PLCb
PKA	-1	NOS1
PKA	-1	NFAT
PKA	-1	LTYPECA
PKA	-1	KV42
PKA	-1	KIR23
PKA	-1	IP3R
PKA	-1	I1
PKA	-1	GABAAR

PKA	-1	D1R
PKA	-1	CAMKK
PKA	-1	BAD
PKA	-1	B2AR
PKA	-1	ADDUCIN
PITPN	0	TCF4
PIR	0	RAC1
PIP5K	1	PIP2
PIP3	1	VAV2
PIP3	1	SH3KBP1
PIP3	1	PKCA
PIP3	1	PDPK1
PIP3	1	ILK
PIP3	1	ARNO
PIP3	1	AKT
PIP2	1	WAVE1
PIP2	1	RYR
PIP2	1	RABPHILIN
PIP2	1	PMCA
PIP2	1	GIRK
PIP2	1	GELSOLIN
PIP2	1	DYNAMIN
PIP2	1	DAG
PIP2	1	ARNO
PIP2	1	AKT
PIP2	-1	CASP9
PIP2	-1	CASP8
PIP2	-1	CASP3
PIP2	-1	bARK
PIN1	1	CTNNB1
PIN1	1	c-JUN
PIN1	0	EPS8
PIM2	-1	BAD
PICK1	-1	MGLUR7
PIB5PA	1	SKP2
PIB5PA	1	PKC
PIB5PA	1	PDK1
PIB5PA	1	NFkB
PIB5PA	1	INPP5A
PIB5PA	1	GELSOLIN
PIB5PA	1	ERK1
PIB5PA	1	DG
PIB5PA	1	DAG

PIB5PA	1	CCR5
PIB5PA	1	CALM3
PIB5PA	1	AHR
PIB5PA	0	VCL
PIB5PA	0	TUBBY
PIB5PA	0	TALIN
PIB5PA	0	PLCy
PIB5PA	0	PLCD1
PIB5PA	0	PLCb
PIB5PA	-1	INPP5A
PIB5PA	-1	GHR
PIAS4	1	TCF4
PIAS4	1	LEF
PIAS	1	SMAD4
PIAS	-1	STAT1
PI3Ky	1	PXN
PI3Ky	1	PTK
PI3K	1	VAV
PI3K	1	RAC1
PI3K	1	PTK
PI3K	1	PIP3
PI3K	1	PIB5PA
PI3K	1	p38
PI3K	1	NFkB
PI3K	1	MEK1
PI3K	1	ILK
PI3K	1	GLUT4
PI3K	1	FBI1
PI3K	1	ERK5
PI3K	1	CDC42
PI3K	1	CCT4
PI3K	1	Akt
PI3K	0	TUBULIN
PI3K	0	PLCy
PI3K	-1	PIP2
PI3K	-1	AKT
PI-4-P5K	1	PIB5PA
PI-4-P5K	1	GELSOLIN
PHKA2	1	PYGB
PGC1A	1	SRA1
PGC1A	1	Era
PGC1A	0	SRC
PGC1A	0	PPARG

PFN1	1	ACTG
PERK	1	EIF2A
PERK	-1	EIF2A
PER	1	CLOCK
PER	1	BMAL1
PER	-1	CYCS
PER	-1	Clk
PER	-1	BMAL1
PELP1	1	SRC
PELP1	1	Estrogen
PELP1	1	ERK1
PELP1	1	CXCR4
PELP1	1	CCR5
PDZGEF	1	RAP2
PDZGEF	1	RAP1
PDPK1	1	RSK
PDPK1	1	PKCz
PDPK1	1	PKCA
PDPK1	1	PKC
PDPK1	1	p90RSK
PDPK1	1	p70s6K
PDPK1	1	AKT
PDK2	1	PKC
PDK2	1	PDK1
PDK2	1	AKT
PDK2	-1	AKT
PDK1	1	AKT
PDI	1	NASCENTCHAIN
PDGFRA	1	STAT3
PDGFRA	1	STAT1
PDGFRA	1	S1P
PDGFRA	1	PI3K
PDGFRA	1	CALM3
PDGFRA	0	SHC
PDGFRA	0	PLCy
PDGFRA	0	PI3K
PDGFR	1	SHP2
PDGFR	1	PLCy
PDGFR	1	APS
PDGFB	0	PDGFRA
PDGF	1	PDGFR
PDE6G	1	PDE6A
PDE6B	0	PDE6G

PDE6A	0	PDE6G
PDE6A	0	PDE6B
PDE6A	-1	CGMP
PDE5A	-1	CGMP
PDE4D	-1	CAMP
PDE4C	-1	CAMP
PDE4B	-1	CAMP
PDE4A	-1	CAMP
PDE3B	-1	CAMP
PDE3A	-1	CAMP
PDE2	-1	CAMP
PDE1C	-1	CGMP
PDE1B	-1	CGMP
PDE1A	-1	CGMP
PDE	-1	CAMP
PCNA	1	CDK4
PCLy	1	PIB5PA
PCAF	1	AR
PCAF	0	NOTCH3
PCAF	0	NICD
PBP	0	TRAF2
PBP	0	PPARG
PAX2	0	POFUT1
PAX2	0	PAX5
PATZ	0	RNF4
PARP	1	NFkB
PARP	1	AIF
PAR4	1	Gy
PAR4	1	GNA13
PAR4	1	GNA12
PAR4	1	Gb
PAR2	1	GBETAGAMMA
PAR2	1	GAQ
PAR1	1	DSH
PAR1	0	PAR4
PAK6	1	AR
PAK2	1	MEKK
PAK1	1	RAF1
PAK1	1	P50
PAK1	1	MRLC
PAK1	1	MEKK
PAK1	1	MEK1
PAK1	1	LIMK1

PAK1	1	JNKK1
PAK1	1	ARP3
PAK1	1	ARP2
PAK1	1	ABL1
PAK1	0	VAV
PAK1	0	PXN
PAK1	0	NCK
PAK1	-1	SYNAPSIN
PAK1	-1	RHOA
PAK1	-1	MLCK
PAK1	-1	BAD
PAFR	1	GBETAGAMMA
PAFR	1	GAQ
PAFR	1	GALPHAO
PAFR	1	GALPHAI
PAF	1	PAFR
PACAP	0	CASP9
PAC1	-1	ERK2
PAC1	-1	ERK1
PABP	1	EIF4F
PABP	0	mRNA
PABP	0	EIF4G
PA	1	SH3KBP1
PA	1	PLCb
PA	-1	PPP1CC
P73	1	Puma
P73	-1	G1
P50	1	RELA
P50	1	p65
P50	1	p300
P50	1	IKBA
P50	1	CDC42
P50	1	ARHGAP1
P50	1	AKT
P50	0	RELA
P50	0	p65
P50	0	GIT1
P21	1	PKLR
P21	1	CHREBP
P21	1	CDK2
P21	1	ARP3
P21	1	ARP2
P21	-1	PCNA

P21	-1	G1
P21	-1	CDK4
P21	-1	CDK2
P21	-1	CDK1
P21	-1	CCNE1
P21	-1	CCNA2
P2	1	GSK3A
P2	1	GAQ
P2	-1	GSK3A
P2	-1	GAQ
P15	-1	CDK6
P1433	1	TP53BP1
P1433	1	bRAF
P1433	-1	RIN1
P1433	-1	RAF1
P1433	-1	NFAT
P1433	-1	IRS1
P1433	-1	FORKHEAD
P1433	-1	COFILIN1
P1433	-1	BAD
P13K	1	SOS1
P13K	1	PI3K
P13K	1	p110Ia
P13K	0	PI3Ky
P130Cas	1	CRK
P130Cas	1	C3G
P130Cas	0	PTK
Omi	-1	XIAP
Omi	-1	HAX1
Omi	-1	APOLLON
O2	1	H2O2
Nucling	1	APAF1
Nrdp1	-1	APOLLON
Noxa	1	BAX
Noxa	1	BAK
Noxa	-1	MCL1
Noxa	-1	BCL2L1
Noxa	-1	BCL2
NUR77	1	BAX
NUR77	-1	BCL2
NUMB	1	NICD
NTYPECA	1	Ca++
NTRK1	1	SHC

NTRK1	1	PLCy
NTRK1	1	PI3K
NTRK1	1	IRS1
NTRK1	1	GRIT
NTRK1	1	GRB2
NTRK1	1	ERK5
NTRK1	1	CRK
NTRK1	0	SHC
NTH1	0	p6
NTF5	1	TRKB
NTF5	0	NTRK1
NRG	1	ERBB2
NOX1	1	O2
NOTCH4	0	PSEN2
NOTCH3	0	SNW1
NOTCH3	0	PSEN2
NOTCH2	0	RNTRE
NOTCH2	0	PSEN2
NOS3	1	PDE2
NOS3	0	CAVEOLIN
NOS3	-1	PDE2
NOS1	1	RHOA
NOS1	1	NO
NOS1	1	CTBP1
NOS1	0	PSD95
NOS1	0	PSD93
NOPR	1	GBETAGAMMA
NOPR	1	GALPHAO
NOPR	1	GALPHAI
NO	1	pGC
NMT	1	BID
NME1	0	SET
NMDAR	1	SAP102
NMDAR	1	PLCy
NMDAR	1	Ca++
NMDAR	0	YOTIAO
NMDAR	0	TUBULIN
NMDAR	0	SSCAM
NMDAR	0	SPECTRIN
NMDAR	0	SAP97
NMDAR	0	RACK
NMDAR	0	PTP1D
NMDAR	0	PSD95

NMDAR	0	PSD93
NLK	-1	TCF
NLK	-1	LEF
NIK	1	NCK
NIK	1	MEKK
NIK	1	IKKA
NIK	1	IKK2
NIK	0	WAVE1
NICD	0	YY1
NICD	0	WDR12
NICD	0	SNW1
NICD	0	RALB
NICD	0	PSEN2
NICD	0	PRKCD
NICD	0	p85beta
NICD	0	NUMBL
NICD	0	NUMB
NICD	0	NOV
NICD	0	MAML3
NICD	0	MAML2
NICD	0	MAML1
NICD	0	JAG2
NICD	0	JAG1
NICD	0	Idax
NICD	0	DTX1
NICD	0	DLL4
NICD	0	DLL1
NICD	0	cPLA2
NICD	0	CNTN1
NICD	0	AIP4
NHERF	0	RACK
NHERF	0	PDGFR
NGFR	1	RAC1
NGFR	0	SHC
NGFR	0	PLCy
NGFR	0	PI3K
NGF	1	NTRK1
NGF	0	NTRK1
NGF	0	NGFR
NFkB	1	IL6
NFkB	1	IL4
NFkB	1	IL2
NFkB	1	IKKy

NFkB	1	IKK2
NFkB	1	IKBA
NFkB	1	IFNy
NFkB	1	FASLG
NFkB	1	ERK1
NFkB	1	CBP
NFkB	-1	IAP
NFkB	-1	FAN
NFYB	0	NFYC
NFYA	0	NFYC
NFYA	0	NFYB
NFAT	1	MEF2
NFAT	1	FASLG
NEUROLIGIN	0	SSCAM
NEUROLIGIN	0	PSD95
NEUROFIBROMIN	-1	RAS
NEUREXIN	0	NEUROLIGIN
NEDD8	1	p27
NE	1	B2AR
NE	1	ALPHA2AR
NE	1	ALPHA1AR
NCOR	0	HERP1
NCOR	-1	PR-A
NCOR	-1	Era
NCK2	0	SNX6
NCK2	0	PKN2
NCK	1	PAK1
NCK	1	N-WASP
NCK	1	ABL1
NCK	0	SNX6
NCK	0	SLP-76
NCK	0	PKN2
NCK	0	GAB2
NCK	0	CAV2
NBS1	0	RAD50
NASCENTCHAIN	0	SEC61P
NAP	0	WAVE3
NAP	0	WAVE2
NAP	0	WAVE1
NAP	0	RAC1
NAP	0	PIR
NAKED	0	DVL3
NAKED	-1	FRIZZLED

NAKED	-1	DSH
NAIP	-1	CASP9
NAIP	-1	CASP7
NAIP	-1	CASP3
N41	1	AMPAR
N41	0	SPECTRIN
N41	0	SAP97
N-WASP	1	ACTIN
N-WASP	0	SNX6
N-WASP	0	SH3GL3
N-WASP	0	RAP74
N-WASP	0	DVL2
Mos	1	MEK2
Mos	1	MEK1
Miz1	1	SMAD4
Miz1	0	REPS2
Miz1	0	RAP74
Miz1	0	PTPN12
Miz1	0	PRKAR1A
Miz1	0	PIK3C2B
Mfn2	0	BAX
Mfn2	0	BAK
MYT1	1	CCNB1
MYT1	-1	CDK1
MYOSINV	1	CAMK2A
MYOD	0	PSMD4
MYOD	0	ID4
MYOD	0	ID3
MYOD	0	ID2
MYOD	0	ID1
MYD88	1	TNF
MYD88	1	NIK
MYD88	1	MEKK
MYD88	1	IRAK4
MYD88	1	IRAK
MYD88	0	TRAF6
MYD88	0	TOLLIP
MYD88	0	TLR9
MYD88	0	TLR8
MYD88	0	TLR7
MYD88	0	TLR6
MYD88	0	TLR5
MYD88	0	TLR4

MYD88	0	TLR3
MYD88	0	TLR2
MYD88	0	TLR10
MYD88	0	TLR1
MYD88	0	TIRAP
MUNC18	0	SYNTAXIN
MUNC13	1	SYNTAXIN
MTOR	1	p70s6K
MTOR	1	EBP
MTOR	-1	PP2A
MTOR	-1	EIF-4EBP
MST1R	0	SFN
MSK2	1	EIF-4EBP
MSK2	1	CREB
MSK2	1	ATF1
MSK1	1	EIF-4EBP
MSK1	1	CREB
MSK1	1	ATF1
MRLC	1	myosin
MRE11	0	RAD50
MP1	1	MAP4K1
MP1	1	ERK2
MOPR	1	GBETAGAMMA
MOPR	1	GALPHAO
MOPR	1	GALPHAI
MNK2	1	EIF4E
MNK1	1	EIF4E
MNK1	0	MNK2
MNK1	0	EIF4G
MMP7	1	ITGB4
MLK3	1	MKK7
MLK3	1	JNKK1
MLK1	1	MKK7
MLK1	1	MEK6
MLK1	1	JNKK1
MLK	1	PSD95
MLK	1	MKK7
MLK	1	JNKK1
MLCP	1	MRLC
MLCP	-1	RLC
MLCP	-1	MYOSIN
MLCK	1	RLC
MLCK	1	myosin

MLCK	1	MRLC
MLCK	1	MLC
MLC	1	PXN
MKP5	-1	p38gamma
MKP5	-1	p38
MKP5	-1	MAPK9
MKP5	-1	MAPK13
MKP5	-1	MAPK11
MKP5	-1	JNK3
MKP5	-1	JNK
MKP4	-1	ERK2
MKP4	-1	ERK1
MKP3	-1	ERK2
MKP3	-1	ERK1
MKP2	-1	MAPK9
MKP2	-1	JNK3
MKP2	-1	JNK
MKP2	-1	ERK2
MKP2	-1	ERK1
MKP1	1	p38
MKP1	1	Hsp90
MKP1	-1	p38gamma
MKP1	-1	p38
MKP1	-1	MAPK13
MKP1	-1	MAPK11
MKP1	-1	JNK
MKP1	-1	ERK2
MKK7	1	MAPK9
MKK7	1	JNK3
MKK7	1	JNK
MITR	1	MEF2
MINT	0	MUNC18
MGLUR7	1	GBETAGAMMA
MGLUR7	1	GALPHAO
MGLUR7	1	GALPHAI
MGLUR7	0	SYNTENIN
MGLUR1	1	TAMALIN
MGLUR1	1	PLCb
MGLUR1	1	Gia
MGLUR1	1	GBETAGAMMA
MGLUR1	1	GAQ
MGLUR1	0	HOMER
MGLUR1	0	DYNAMIN

MFNG	1	NOTCH2
MFNG	1	JAG1
MFNG	1	DLL1
METS	1	E2F4
METS	1	DP103
METS	0	SMRT
MET	1	STAT3
MET	0	SMRT
MET	0	PTK
MET	-1	FAS
MERLIN	0	SYNTENIN
MEKK4	1	MKK7
MEKK4	1	MAP4K6
MEKK4	1	MAP4K3
MEKK4	1	JNKK1
MEKK4	0	N-WASP
MEKK4	0	CTNND1
MEKK4	0	AML3
MEKK3	1	MKK7
MEKK3	1	MEK5
MEKK3	1	MAP4K3
MEKK3	1	JNKK1
MEKK3	0	MEK5
MEKK2	1	JNKK1
MEKK	1	TAK1
MEKK	1	SMRT
MEKK	1	SLC9A1
MEKK	1	SEK
MEKK	1	p38
MEKK	1	MKK7
MEKK	1	MEK3
MEKK	1	MEK1
MEKK	1	JNKK1
MEKK	1	JNK
MEKK	1	IKKA
MEKK	0	NIK
MEK6	1	p38gamma
MEK6	1	p38
MEK6	1	NFkB
MEK6	1	MAPK13
MEK6	1	MAPK11
MEK6	1	JNK
MEK5	1	ERK5

MEK5	0	PRKCI
MEK5	0	PKCz
MEK5	0	GATA1
MEK3	1	p38
MEK3	1	MAPK13
MEK3	1	MAPK11
MEK3	1	JNK
MEK3	0	MEK6
MEK2	1	MEK1
MEK2	1	HSP27
MEK2	1	ERK2
MEK2	1	ERK1
MEK2	1	CREB
MEK1	1	SMRT
MEK1	1	RSK2
MEK1	1	p90RSK
MEK1	1	p70s6K
MEK1	1	MSK2
MEK1	1	MSK1
MEK1	1	MLCK
MEK1	1	MCL1
MEK1	1	MAPK9
MEK1	1	JNKK1
MEK1	1	FOS
MEK1	1	ERK2
MEK1	1	ERK1
MEK1	1	ELK1
MEK1	1	CREB
MEK1	1	Calpain1
MEK1	1	BCL2
MEK1	1	ACTIN
MEK1	0	MEK2
MEK1	-1	SOS1
MEK1	-1	FRS2
MEK1	-1	BIM
MEF2D	1	PPP3R2
MEF2C	0	NFAT
MEF2C	0	MLC
MEF2	1	MITR
MEF2	1	HDAC5
MEF2	1	c-JUN
MEF2	0	MYOD
MEF2	0	MITR

MDM2	1	p53
MDM2	1	BETAARRESTIN
MDM2	0	p53
MDM2	-1	TP53BP1
MDM2	-1	p300
MD-2	0	TLR4
MCL1	-1	CYTOCHROMEC
MCL1	-1	Bok
MCL1	-1	BIM
MCL1	-1	BAX
MCL1	-1	BAK
MBD3	-1	SNAI1
MARCKS	0	ACTIN
MARCKS	-1	PPP3R2
MAPKAP2	1	MSK1
MAPKAP2	1	HSP27
MAPKAP2	1	HMGN1
MAPKAP-K3	1	HSP27
MAPK9	1	SHC
MAPK9	1	RNPK
MAPK9	1	PTK
MAPK9	1	p90RSK
MAPK9	1	p53
MAPK9	1	NFAT
MAPK9	1	MIP1B
MAPK9	1	IL4
MAPK9	1	IL2
MAPK9	1	GDP
MAPK9	1	G1
MAPK9	1	FOS
MAPK9	1	ERK1
MAPK9	1	c-JUN
MAPK9	1	ATF2
MAPK9	-1	JAK2
MAPK9	-1	FOS
MAPK13	1	EEF2K
MAPK13	1	CEBPA
MAPK11	1	MSK2
MAPK11	1	MSK1
MAPK11	1	MNK2
MAPK11	1	MNK1
MAPK11	1	MEK2
MAPK11	1	MEF2

MAPK11	1	MAPKAP-K3
MAPK11	1	ELK1
MAPK11	1	CHOP10
MAP4K6	1	p38
MAP4K5	1	MEKK
MAP4K3	1	p38
MAP4K1	1	JNK
MAP4K1	1	ERK2
MAP4K1	0	KSR
MAP3K2	1	PRK2
MAP3K2	1	MKK7
MAP3K2	1	MEK5
MAP3K2	1	JNKK1
MAP3K2	1	ERK2
MAP3K2	0	WNT2
MAP2	0	TUBULIN
MAP1B	0	TUBULIN
MAP1A	0	TUBULIN
MAP-1	0	BCL2
MAP-1	0	BAX
MAP-1	-1	BCL2L1
MAML3	0	NOTCH4
MAML3	0	NOTCH3
MAML3	0	NOTCH2
MAML2	0	NOTCH4
MAML2	0	NOTCH3
MAML2	0	NOTCH2
MAML1	0	NOTCH4
MAML1	0	NOTCH3
MAML1	0	NOTCH2
MALS	0	PSD95
MALS	0	NMDAR
MAL	0	SRF
MAGI3	0	VANGL2
MAGEA1	0	SNW1
MADD	1	JNKK1
MADD	0	TRADD
MADD	0	TNFR1
MADD	0	SODD
M4R	1	GBETAGAMMA
M4R	1	GALPHAI
M4R	1	EEF1A2
M3/6	-1	MAPK9

M3/6	-1	JNK3
M3/6	-1	JNK
M2R	1	GBETAGAMMA
M2R	1	GALPHAO
M2R	1	GALPHAI
M2R	0	RGS2
M1R	1	Gia
M1R	1	GBETAGAMMA
M1R	1	GAQ
M1R	0	RGS2
Livin	-1	CASP9
Livin	-1	CASP7
Livin	-1	CASP3
Lgs	1	CTNNB1
Lgs	0	EPS8
LZK	1	MKK7
LZK	1	JNKK1
LYN	1	VAV
LYN	1	TUBULIN
LYN	1	SHC
LYN	1	RAC1
LYN	1	PLCy
LYN	1	P13K
LYN	1	CD19
LYN	1	BTK
LYN	1	BCR
LYN	0	SYK
LYN	0	PSD95
LYN	0	FCER1A
LYN	0	CD19
LXR	1	FXR
LXR	0	RXR
LTYPECA	1	Ca++
LTYPECA	0	SORCIN
LTYPECA	0	PSD95
LTYPECA	0	MAP2
LRP6	0	DKK2
LRP	1	WNT
LRP	1	FRIZZLED
LRP	0	PLSCR1
LRP	-1	AXIN
LPS	0	TLR4
LMNB1	0	LMNB2

LMNA	0	LMNB2
LMNA	0	LMNB1
LIMK1	1	APAF1
LIMK1	-1	COFILIN2
LIMK1	-1	COFILIN1
LFNG	1	NOTCH2
LFNG	1	NICD
LEPR	1	AMPK
LEP	1	LEPR
LEF1	1	MITF
LEF	0	SUMO2
LEF	0	SF1
LEF	0	PIAS4
LEF	0	NICD
LEF	0	EPS8
LEF	0	CDX1
LCK	1	ZAP70
LCK	1	VAV2
LCK	1	RAF1
LCK	1	PLCy
LCK	1	IBP
LCK	1	DAP10
LCK	1	CD3
LCK	0	ZAP70
LCK	0	SOS1
LAT	1	PLCy
LAT	1	GRB2
LAT	0	PLCy
LAT	0	Gads
LARG	1	ARHGAP1
LAMR1	0	ITGA6
LAMB1	1	Ub
LAMB1	0	LAMC1
LAMA1	0	LAMB1
L1	1	VIL2
Ku70	-1	BAX
KV42	1	POTASSIUM
KV41	1	POTASSIUM
KV41	0	PSD95
KV14	1	POTASSIUM
KV12	1	POTASSIUM
KV12	0	PSD95
KV11	1	POTASSIUM

KSR	1	RAF1
KSR	0	P1433
KRT18	0	TCF4
KRT18	0	STAT5B
KRT18	0	PIK3CD
KRT18	0	Miz1
KOPR	1	GBETAGAMMA
KOPR	1	GALPHAO
KOPR	1	GALPHAI
KIT	1	VAV
KIT	1	STAT5B
KIT	1	STAT5
KIT	1	STAT1
KIT	1	SPRED2
KIT	1	SPRED1
KIT	0	TCF4
KIT	0	STAP1
KIT	0	SPRED1
KIT	0	SOCS6
KIT	0	SOCS5
KIT	0	SOCS1
KIT	0	PTPRO
KIT	0	PTCH2
KIT	0	PLSCR1
KIT	0	PLCE1
KIT	0	MUPP1
KIT	0	Miz1
KIR41	1	POTASSIUM
KIR23	1	POTASSIUM
KIR23	0	PSD95
KIR21	1	POTASSIUM
KIR21	0	PSD95
KB	0	NFkB
KAR	0	SYNTENIN
KAR	0	PSD95
KAR	0	PICK1
KAR	0	NCADHERIN
K+	1	AKT
JUNB	1	GATA3
JUNB	0	NFAT
JNKK1	1	p38
JNKK1	1	MAPK9
JNKK1	1	MAPK11

JNKK1	1	JNK3
JNKK1	1	JNK
JNKK1	-1	IRS2
JNK3	1	SHC
JNK3	1	RNPK
JNK3	1	p53
JNK3	1	NFAT
JNK3	1	c-JUN
JNK3	1	ATF2
JNK	1	TCF
JNK	1	TAU
JNK	1	SHC
JNK	1	RNPK
JNK	1	p53
JNK	1	NFAT
JNK	1	MAPK9
JNK	1	FOS
JNK	1	FASLG
JNK	1	ELK1
JNK	1	CASP1
JNK	1	c-JUN
JNK	1	Bmf
JNK	1	BIM
JNK	1	BID
JNK	1	BCL2
JNK	1	BAD
JNK	1	ATF2
JNK	-1	MEK1
JNK	-1	MCL1
JNK	-1	BCL2
JIP	1	TIAM1
JIP	1	RASGRF
JIP	1	p38
JIP	1	MLK3
JIP	1	MKK7
JIP	1	JNKK1
JIP	1	DLK
JIP	0	POFUT1
JIP	0	PAX2
JIP	0	KINESIN
JIP	-1	JNK
JAK3	1	JAK1
JAK2	1	TYK2

JAK2	1	STAT5
JAK2	1	STAT3
JAK2	1	STAT1
JAK2	1	p65
JAK2	1	JAK1
JAK2	0	TID1
JAK1	1	STAT6
JAK1	1	STAT3
JAK1	1	STAT2
JAK1	1	STAT1
JAK1	1	STAT
JAK1	1	SHC
JAK1	1	JAK3
JAK1	1	JAK2
JAK1	0	PI3K
JAK1	0	PDGFRA
JAG2	0	PPP2CA
JAG2	0	NOTCH3
JAG2	0	NOTCH2
JAG1	0	PPP2CA
JAG1	0	NOTCH3
JAG1	0	NOTCH2
Itk	1	PLCy
Itk	0	SLP-76
Insulin	1	INSR
Ins	1	INSR
Importin	1	RAN
Importin	0	RAN
Idax	0	NOTCH4
Idax	0	HEY1
Idax	-1	DSH
ITGB4	0	VIM
ITGB4	0	STAT5B
ITGB4	0	RNTRE
ITGB4	0	PIK3CD
ITGB4	0	ITGB4BP
ITGB3	0	VIL2
ITGB3	0	TALIN
ITGB3	0	PXN
ITGB1	1	SHC
ITGB1	1	PTK
ITGB1	1	JAK2
ITGB1	1	ILK

ITGB1	1	FKHRL1
ITGB1	1	FAK
ITGB1	1	ERK1
ITGB1	0	TALIN
ITGB1	0	SPTAN1
ITGB1	0	SHC
ITGB1	0	RACK
ITGB1	0	PXN
ITGB1	0	PTK
ITGB1	0	ALPHAACTININ
ITGB1	-1	SHC
ITGA6	0	ITGB4
ITGA1	1	XIAP
ITGA1	1	PI3K
ITGA1	1	BIRC5
ITGA1	0	VIL2
ITGA1	0	TALIN
ITGA1	0	SPTAN1
ITGA1	0	PXN
ITGA1	0	PTK
ITGA1	0	ITGB3
ITGA1	0	ITGB1
ITGA1	-1	REQ
ITGA1	-1	FOS
ITGA1	-1	CASP3
ISRE	1	TBK1
ISRE	1	IKKe
ISRE	0	TLR4
ISRE	0	TLR3
ISRE	0	STAT3
IRS2	1	PI3K
IRS2	1	p85beta
IRS2	1	P13K
IRS1	1	PI3K
IRS1	1	NCK
IRS1	1	GRB2
IRS1	1	ERK1
IRS1	0	RET
IRS1	0	PI3K
IRAK4	1	TRAF6
IRAK-M	-1	IRAK4
IRAK-M	-1	IRAK
IRAK	1	TRAF6

IRAK	1	TLR4
IRAK	1	TLR2
IRAK	1	TAB2
IRAK	1	TAB1
IRAK	0	TRAF6
IRAK	0	TOLLIP
IRAK	0	MYD88
IQGAP	-1	RAS
IP3R	1	CRAC
IP3R	1	Ca++
IP3R	0	HOMER
IP3R	0	FKBP
IP3R	0	CYTOCHROMEC
IP3R	0	ANKYRIN
IP3	1	IP3R
INSR	1	STAT5
INSR	1	SHC
INSR	1	MEK6
INSR	1	IRS2
INSR	1	IRS1
INSR	1	GRB10
INSR	1	APS
INSR	0	Insulin
INPP5A	1	PIB5PA
INPP5A	1	PDK1
INPP5A	1	GEF
INPP5A	1	AKT
INHIBITOR2	-1	PPP1CC
ILPIP	1	XIAP
ILK	1	CPI17
ILK	1	AKT
ILK	-1	GSK3A
IL9	0	p16
IL6R	1	JAK1
IL6	1	IL6R
IL4R	1	JAK1
IL4R	1	IL2RG
IL4R	1	IL13RA1
IL4	1	IL4R
IL3	0	SCF
IL3	0	IL9
IL3	0	IL6
IL2RG	1	JAK3

IL1R	1	TRAF6
IL1R	1	ECSIT
IL1R	0	TRAF6
IL1R	0	TOLLIP
IL1B	1	MEK3
IL1B	1	IL1R
IL1B	0	IL1R
IL1A	1	IL8
IL1A	0	TNF
IL1A	0	IL3
IL1A	0	IL1R
IL1A	0	IL1B
IL1A	0	IL12
IL13RA2	-1	IL4R
IL13RA1	1	TYK2
IL13RA1	1	JAK2
IL13RA1	1	IL4R
IL12RB2	1	JAK2
IL11	0	IL6
IL-13	1	IL13RA2
IL-13	1	IL13RA1
IKKy	1	NFkB
IKKy	1	IKBA
IKKe	1	IRF3
IKKA	1	NFkB
IKKA	1	IKBA
IKKA	0	NIK
IKKA	0	MEKK
IKKA	0	IKKy
IKK2	1	NFkB
IKK2	1	IKBA
IKK2	0	NIK
IKK2	0	MEKK
IKK2	0	IKKy
IKK2	0	IKKA
IKBB	1	Hypoxia
IKBB	-1	NFkB
IKBA	1	RELA
IKBA	1	PKC
IKBA	1	NFkB
IKBA	1	IL4
IKBA	1	IL2
IKBA	0	Ub

IKBA	0	RELA
IKBA	0	p65
IKBA	0	P50
IKBA	0	NFkB
IKBA	-1	NFkB
IKBA	-1	IL4
IKBA	-1	IL2
IHH	0	PTCH2
IHH	0	PTCH
IGF1R	1	VAV2
IGF1R	1	SHC
IGF1R	1	PPP3R2
IGF1R	1	PKCA
IGF1R	1	PI3K
IGF1R	1	NFAT
IGF1R	1	IRS1
IGF1R	1	GRB10
IGF1R	1	ERK2
IGF1R	1	ERK1
IGF1R	1	CAMK1
IGF1R	1	BETAARRESTIN
IGF1R	1	AKT
IGF1R	0	LARG
IGF1	1	IGF1R
IGF1	0	IGF1R
IFNy	1	JAK2
IFNy	1	IFN-yR
IFNy	0	IFN-yR
IFNAR	1	TYK2
IFNAR	1	JAK1
IFNA	1	IFNAR
IFN-yR	1	TIP1
IFN-yR	1	TID1
IFN-yR	1	JAK2
IFN-yR	1	JAK1
IFN-yR	1	hsp70
IFN-yR	0	TID1
IFN-yR	0	JAK2
ID3	0	TCF12
ID3	0	PAX5
ID3	0	MYOG
ID3	0	MYF6
ID3	0	MYF5

ID2	0	TCF3
ID2	0	TCF12
ID2	0	PAX8
ID2	0	PAX5
ID2	0	PAX2
ID2	0	MYOG
ID2	0	MYF6
ID2	0	MYF5
ID2	0	ID3
ID1	0	TCF3
ID1	0	TCF12
ID1	0	PSMD4
ID1	0	MYOG
ID1	0	MYF6
ID1	0	MYF5
ICAT	0	EPS8
ICAT	-1	CTNNB1
ICAD	1	CAD
ICAD	0	TRADD
ICAD	0	TNFR1
ICAD	0	RIP
ICAD	-1	DFFB
ICAD	-1	CAD
ICA512	0	SPECTRIN
IC261	-1	CK1d
IBR	1	SHP1
IBP	1	SYK
IAP	-1	CASP9
I1	-1	PPP1CC
I-mf	-1	TCF
I-FLICE	1	FADD
I-FLICE	-1	FADD
I-FLICE	-1	CASP8
Hypoxia	1	p53
Hypoxia	1	HUR
Hypoxia	-1	AKT
Hsp90	1	PTEN
Hsp90	1	BIRC5
Hsp90	1	AHR
Hsp90	0	XAP2
Hsp90	0	PPARA
Hsp90	0	NOS3
Hsp90	-1	APAF1

Hsp60	0	CASP6
Hsp60	0	CASP3
Hsp40	1	Ub
Hsp40	0	LAMR1
Hsp40	0	hsp70
Hsp40	0	BIP
Hsp40	-1	MDM2
Hrk	0	MCL1
Hrk	-1	BCL2L1
Hrk	-1	BCL2
Hrk	-1	Bcl-w
HUS1	1	ATR
HUS1	0	RAD9
HUS1	0	RAD1
HSPG	1	FGFR
HSPG	1	FGF
HSPC	1	PIR
HSPC	0	WAVE3
HSPC	0	WAVE2
HSPC	0	WAVE1
HSP72	-1	AIF
HSP27	0	PDZK1
HSP27	-1	DAXX
HSP27	-1	CYTOCHROMEC
HSP27	-1	CASP3
HRI	-1	EIF2A
HOP	0	SRF
HOMER	1	RYR
HOMER	0	SHANK
HMG2	0	SET
HMG2	0	PP32
HMG1	0	TopoII
HMG1	0	HMG2
HIPPOCALCIN	1	NAIP
HIPK2	1	SF1
HIPK2	0	SF1
HIPK2	0	MYB
HIPK2	0	Miz1
HIPK2	0	ITGB4
HINT1	1	RAF1
HIF1A	1	P50
HIF1A	1	Noxa
HIF1A	1	BNIP3L

HIF1A	1	BNIP3
HIF1A	0	MDM2
HIF1A	-1	BID
HHIP	0	IHH
HGF	0	MET
HES6	0	RAP30
HES5	0	PLSCR1
HES1	0	RAP30
HES1	0	PLSCR1
HES1	0	ID4
HES1	0	ID3
HES1	0	ID2
HES1	0	ID1
HES1	0	HES6
HERP1	0	HES1
HDAC5	0	MEF2
HDAC3	1	p300
HDAC3	0	SMRT
HDAC3	0	NCOR
HDAC2	0	SMRT
HDAC2	0	Sin3
HDAC2	0	MEF2D
HDAC2	0	CIR
HDAC1	0	MEF2D
HDAC1	0	HDAC2
HDAC1	-1	SNAI1
HDAC	1	LEF
HDAC	1	GROUCHO
HDAC	0	RB
HD	0	Miz1
HD	0	HIP1
HCK	1	PTP-SL
HCK	1	PLCy
HCK	1	GJA1
HCK	1	ELK4
HCK	0	PTP-SL
HCK	0	MEK5
HCK	0	KIT
HCK	0	GJA1
HBX	1	PTK
HBX	1	Ca++
HBX	-1	CASP9
HBX	-1	Ca++

HBO1	1	TIEG2
HBO1	1	TGIF
HBO1	1	TCF3
HBO1	1	STEP
HBO1	1	STAT3
HBO1	1	SMAD3
HBO1	1	SMAD2
HBO1	1	SH2D3C
HBO1	1	ROC1
HBO1	1	RNTRE
HBO1	1	PTP-SL
HBO1	1	PPP2CA
HBO1	1	GRB10
HBO1	1	CIR
HBO1	1	AR
HBO1	0	UXT
HBO1	0	UBE3A
HBO1	0	TNIP1
HBO1	0	SNX1
HBO1	0	RKIP
HBEGF	1	EGFR
HAT1	1	TGIF
HAT1	1	TCF3
HAT1	1	STEP
HAT1	1	STAT3
HAT1	1	SHP2
HAT1	1	PTP-SL
HAT1	1	PPP2CA
HAT1	1	HIST3H3
HAT1	1	GRB10
HAT1	0	UXT
HAT1	0	UBE3A
HAT1	0	SNX1
HAT1	0	RANBP9
HAT1	0	HBO1
HAND1	0	HAND2
H2O2	1	HO
H2O2	1	BAX
H1	0	HMG2
Gy	1	SHC
Gy	1	S1P
Gy	1	RAC1
Gy	1	PLCy

Gy	1	MEKK
Gy	1	ITGA1
Gy	1	Gb
Gy	1	GA
Gy	1	ERK2
Gy	1	ERK1
Gy	0	RAC1
Gy	0	PLCb
Gy	-1	ERK1
Grb7	0	SENP1
Grb7	0	KIT
Graf	-1	Rho
Gia	1	PLCb
Gia	1	MEK3
Gia	1	Gbg
Gia	1	GAQ
Gia	1	CCNB1
Gia	1	AC
Gia	0	MPR
Gia	-1	MYT1
Gbg	1	RAC1
Gbg	1	PLC
Gbg	1	PI3K
Gbg	1	HBEGF
Gbg	1	EGFR
Gbg	1	bARK
Gbg	1	AC
Gbg	-1	AC
Gb	1	PI3K
Gb	1	Gy
Gb	1	GEF
Gb	1	Gai
Gb	0	TUBBY
Gb	0	PAR4
Gb	0	Gy
Gb	0	GNA13
Gb	0	GNA12
Gb	0	Gia
Gat2	1	PDE6G
Gas	1	AC
Gai	1	SRC
Gai	1	RAP1GAP
Gai	1	RAC1

Gai	1	PI3Ky
Gai	1	PI3K
Gai	1	Gbg
Gai	1	bRAF
Gai	0	Gy
Gai	0	GTP
Gai	0	GDP
Gai	0	Gb
Gai	-1	Gb
Gai	-1	AC
Gads	0	SLP-76
GZMB	1	RAB3
GZMB	1	CASP9
GZMB	1	BID
GZMB	0	IP6K2
GZMB	-1	CASP9
GTP	1	WAVE3
GTP	1	WAVE2
GTP	1	WAVE1
GTP	1	ROCK1
GTP	1	RHOA
GTP	1	RAN
GTP	1	RAF1
GTP	1	RAC1
GTP	1	POR1
GTP	1	PLD1
GTP	1	PLCE
GTP	1	PAK1
GTP	1	p70s6K
GTP	1	P35
GTP	1	MEKK
GTP	1	MEK1
GTP	1	GDP
GTP	1	DIA
GTP	0	SAC
GTP	0	RAN
GTP	0	RAC1
GTP	0	AC1
GTP	-1	RAC1
GSK3B	1	TAU
GSK3B	1	GSK3A
GSK3B	1	CTNNB1
GSK3B	1	AXIN

GSK3B	1	AKT
GSK3B	-1	SMO
GSK3B	-1	EIF2A
GSK3B	-1	CCND1
GSK3A	1	MITF
GSK3A	1	INHIBITOR2
GSK3A	1	CREB
GSK3A	1	AXIN
GSK3A	1	ANAPC1
GSK3A	0	FRAT2
GSK3A	-1	TAU
GSK3A	-1	PSEN1
GSK3A	-1	NFAT
GSK3A	-1	eIF2B
GSK3A	-1	CTNNB1
GSK3A	-1	c-JUN
GRP94	0	NASCENTCHAIN
GRP1	1	p14ARF
GROUCHO	1	TCF
GROUCHO	1	LEF
GROUCHO	0	TCF
GROUCHO	0	SMAD4
GRIT	-1	CDC42
GRIT	-1	ARHGAP1
GRIT	-1	AKT
GRIP1	0	STAMBPL1
GRIP	0	MGLUR7
GRIP	0	KAR
GREB1	0	PDZK1
GRE	0	SRF
GRB2	1	WAVE1
GRB2	1	VAV2
GRB2	1	SPRY
GRB2	1	SOS1
GRB2	1	SHC
GRB2	1	SAM68
GRB2	1	RAC1
GRB2	1	PLCy
GRB2	1	GAB1
GRB2	1	GAB
GRB2	1	ERK1
GRB2	1	DYNAMIN
GRB2	1	CBL

GRB2	0	TUBULIN
GRB2	0	SPRY
GRB2	0	SOS1
GRB2	0	SLP-76
GRB2	0	SHP2
GRB2	0	SHC
GRB2	0	SH3KBP1
GRB2	0	REPS1
GRB2	0	RASGRF
GRB2	0	PTK
GRB2	0	PDGFRA
GRB2	0	NTRK1
GRB2	0	MET
GRB2	0	LAT
GRB2	0	DAP10
GRB2	0	APS
GRB2	-1	PTPA
GRB14	0	SENP1
GRB10	1	PI3K
GRB10	0	SENP1
GRB10	0	REPS1
GRB10	0	POFUT1
GRB10	0	Miz1
GRB10	0	KIT
GRB10	0	ID3
GRB10	0	ID2
GRB10	0	ID1
GRB10	0	HBO1
GRB10	0	HAT1
GRB10	0	GAB2
GRB10	0	CITED1
GRB10	0	AR
GRAP	0	KIT
GR	1	PI3K
GR	1	MKP1
GR	0	GRE
GPCR	1	ANF
GPCR	0	TGM2
GNA16	1	PLCb
GNA16	1	DAG
GNA13	1	RHOGEFs
GNA13	1	RADIXIN
GNA13	1	PTK

GNA13	1	CDC42
GNA13	1	ASK1
GNA13	1	ARHGEF1
GNA13	0	Gy
GNA13	0	GPCR
GNA12	1	TEC
GNA12	1	SRC
GNA12	1	RHOGEFs
GNA12	1	RHOA
GNA12	1	RASGAP
GNA12	1	PLD
GNA12	1	MEK5
GNA12	1	ARHGEF1
GNA12	1	AKAP13
GNA12	0	Gy
GNA12	0	GPCR
GNA12	0	EDG2
GMCSF	0	SCF
GMCSF	0	IL3
GLYT1	-1	GLYCINE
GLYCINE	1	NMDAR
GLYCINE	1	GLYR
GLUTAMATE	1	NMDAR
GLUTAMATE	1	MGLUR7
GLUTAMATE	1	MGLUR1
GLUTAMATE	1	KAR
GLUTAMATE	1	AMPAR
GKAP	1	NOS1
GKAP	0	SHANK
GIT1	0	TCF4
GIRK	1	POTASSIUM
GHR	1	PI3K
GHR	1	IGF1
GHR	0	JAK2
GHR	0	IRS1
GH	1	GHR
GH	0	GHR
GFR	1	PKAc
GFR	0	PI3K
GFR	0	GRB2
GFAP	1	Ub
GEPHYRIN	0	TUBULIN
GEPHYRIN	0	GLYR

GELSOLIN	0	PROFILIN
GELSOLIN	-1	CASP9
GELSOLIN	-1	ACTIN
GEF	1	Rho
GEF	1	RAC1
GEF	1	ARHGAP1
GEF	1	ARF1
GEF	1	AKT
GEF	0	TUBULIN
GDP	1	RHOA
GDP	1	RAN
GDP	1	RAF1
GDP	1	RAC1
GDP	1	PAK2
GDP	1	P13K
GDP	1	Importin
GDP	1	GTP
GDP	1	EIF2A
GDP	0	RAN
GDP	0	RAC1
GDNF	1	RET
GCSF	0	SCF
GCSF	0	IL11
GCN2	-1	EIF2A
GCK	1	MEKK
GCAP	1	GC2
GC1	1	GTP
GBETAGAMMA	1	SYNTAXIN
GBETAGAMMA	1	RASGAP
GBETAGAMMA	1	PQCaCh
GBETAGAMMA	1	PLCb
GBETAGAMMA	1	PI3K
GBETAGAMMA	1	KV11
GBETAGAMMA	1	IRS1
GBETAGAMMA	1	GIRK
GBETAGAMMA	1	bARK
GBETAGAMMA	1	AC2
GBETAGAMMA	-1	NTYPECA
GBETAGAMMA	-1	AC1
GATA4	1	SRF
GATA4	1	HOP
GATA4	0	SRF
GATA4	0	NFAT

GATA4	0	MEF2C
GATA1	0	HEY1
GAT1	0	SYNTAXIN
GAT1	-1	GABA
GAS1	0	POLR2A
GAQ	1	PLCb
GAQ	1	PLC
GAQ	1	MEK1
GAQ	1	GTP
GAQ	0	PLCy
GAQ	0	PLCb
GAQ	0	Gy
GAQ	0	GTP
GAQ	0	GPCR
GAQ	0	GDP
GAQ	0	Gb
GAP	1	RAC1
GAP	1	GTP
GAP	0	PDGFRA
GALPHAZ	-1	AC5
GALPHAZ	-1	AC1
GALPHAS	1	AC5
GALPHAS	1	AC2
GALPHAS	1	AC1
GALPHAI	-1	AC5
GALPHAI	-1	AC2
GAIP	-1	GAQ
GADD45	1	MEKK4
GADD45	-1	CDK1
GABARAP	0	TUBULIN
GABARAP	0	GEPHYRIN
GABABR	1	GBETAGAMMA
GABABR	1	GALPHAO
GABAAR	1	CHLORIDE
GABAAR	0	GABARAP
GABA	-1	GABABR
GAB2	1	VAV2
GAB2	1	TCF4
GAB2	1	STAT5B
GAB2	1	STAT3
GAB2	1	STAT1
GAB2	1	SHP1
GAB2	1	SHC

GAB2	1	RGS16
GAB2	1	RASGAP
GAB2	1	PLD
GAB2	1	Miz1
GAB2	1	KRT18
GAB2	1	GRB10
GAB2	1	EPS8
GAB2	1	EPS15
GAB2	1	CEACAM1
GAB2	0	ZNF259
GAB2	0	VAV3
GAB2	0	UBE3A
GAB2	0	SOCS3
GAB2	0	SOCS1
GAB2	0	SNRPD2
GAB2	0	SHIP2
GAB2	0	SH3BGRL
GAB2	0	SENP1
GAB2	0	PTK6
GAB2	0	PRKAR1A
GAB2	0	PLSCR1
GAB2	0	PITPN
GAB2	0	PIK3C2B
GAB2	0	NCK2
GAB2	0	Miz1
GAB2	0	MIG6
GAB2	0	KRT8
GAB2	0	KRT7
GAB2	0	KRT17
GAB2	0	HIST3H3
GAB2	0	HD
GAB2	0	HBO1
GAB1	1	PI3K
GAB1	1	P13K
GAB1	1	MAP4K1
GAB1	1	CRK
GAB1	0	SHP2
GAB1	0	PXN
GAB1	0	PTK
GAB1	0	MET
GAB1	0	GRB2
GAB1	-1	PI3K
GAB1	-1	MEKK3

GAB	1	p85
GAA	0	GTP
GAA	0	GDP
GAA	0	Gb
GA	1	PI3Ky
GA	1	Gy
GA	1	GAQ
GA	1	ERK1
GA	1	DSH
GA	0	PLCy
GA	0	Gy
GA	0	GPCR
GA	0	Gb
GA	0	AC1
G3PD	0	HSP27
G2	0	Gb
G1	0	Gy
G1	0	Gb
G1	0	GA
Forskolin	1	AC
Fibrinogen	1	ITGB1
Fe	1	H2O2
FZD9	0	WNT2
FZD8	0	PSMD4
FZD8	0	LRP5
FZD7	0	MAGI3
FZD6	0	WNT4
FZD5	0	WNT7A
FZD5	0	ROR2
FZD5	0	PSMD4
FZD4	0	MAGI3
FZD2	1	SRF
FZD2	1	EPS8
FZD2	1	DVL2
FZD2	1	DISHEVELED
FZD2	0	UBE3A
FZD2	0	TFDP2
FZD2	0	STAMBPL1
FZD2	0	SNIP1
FZD2	0	SHIP2
FZD2	0	ROR2
FZD2	0	JAG1
FYN	1	TUBULIN

FYN	1	STAT2
FYN	1	SHC
FYN	1	PLCy
FYN	1	NMDAR
FYN	1	LTYPECA
FYN	1	DAB1
FYN	1	CTNNB1
FYN	1	AMPAR
FYN	0	SHC
FYN	0	SAM68
FYN	0	PSD95
FYN	0	PSD93
FYN	0	JAK2
FYN	0	ITGB1
FXR	0	RXR
FURIN	1	NICD
FRS2	1	SHP2
FRS2	1	GRB2
FRP	0	WNT4
FRP	-1	WNT
FRP	-1	FRIZZLED
FRIZZLED	1	PLASMIN
FRIZZLED	1	GA
FRIZZLED	1	DSH
FRIZZLED	1	BETAARRESTIN
FRIZZLED	0	WNT
FRIZZLED	0	LRP6
FRIZZLED	-1	PLASMIN
FRAT2	0	RNTRE
FRAT1	-1	GSK3B
FRAT1	-1	GSK3A
FOXG1B	0	FOXH1
FOUREBP1	1	EIF4E
FOSB	0	JUND
FOS	1	c-JUN
FOS	0	JUND
FOS	0	JNK
FLT3	0	SCF
FLASH	1	CASP8
FKHRL1	1	FASLG
FKHRL1	1	BIM
FKBP	1	CALCINEURIN
FKBP	0	RYR

FIVEHT4R	1	GBETAGAMMA
FIVEHT4R	1	GALPHAS
FIVEHT2AR	1	GBETAGAMMA
FIVEHT2AR	1	GAQ
FIVEHT1AR	1	GBETAGAMMA
FIVEHT1AR	1	GALPHAZ
FIVEHT1AR	1	GALPHAS
FIVEHT1AR	1	GALPHAI
FILAMIN	0	AR
FIBRONECTIN	1	ITGB1
FHL2	0	PSMD4
FHL2	0	LRP5
FHL1	0	RING1
FHL1	0	HIVEP3
FGFR	1	STAT
FGFR	1	SHC
FGFR	1	PLCy
FGFR	1	FRS2
FGF	1	HSPG
FGF	1	FGFR
FER	1	CTNNB1
FBXO6	0	SKP2
FBXO6	0	SKP1
FBW7	0	SKP1
FBW7	0	FBXO6
FASLG	1	FAS
FASLG	-1	FAS
FAS	1	SHP1
FAS	1	RIP
FAS	1	NFkB
FAS	1	IL1A
FAS	1	FADD
FAS	1	EGFR
FAS	1	DAXX
FAS	0	VIL2
FAS	0	LFG
FAS	0	ICAD
FAS	0	FASLG
FAS	0	FAP-1
FAS	0	FAF
FAS	0	Alg-2
FAS	-1	FASLG
FAP-1	1	FADD

FAP-1	1	CASP8
FAP	-1	FANCF
FAP	-1	CASP8
FANCF	0	FAS
FANCF	0	FANCG
FANCE	1	FANCD2
FANCE	0	FANCG
FANCE	0	FANCF
FANCD2	0	Ub
FANCD2	0	FANCE
FANCC	0	FANCG
FANCC	0	FANCE
FANCA	0	FANCG
FANCA	0	FANCC
FAN	1	SMPD2
FAN	0	TNFR1
FAK	1	SRC
FAK	1	SHC
FAK	1	RPTK
FAK	1	PXN
FAK	1	PLCy
FAK	1	PI3K
FAK	1	P130Cas
FAK	1	IL6
FAK	1	Grb7
FAK	1	GRB2
FAK	1	Graf
FAK	1	FYN
FAK	1	ERK2
FAK	1	ASAP1
FAK	0	TALIN
FAK	0	SRC
FAK	0	PXN
FAK	0	PTK
FAK	0	P130Cas
FAK	0	ITGB1
FAK	0	ITGA1
FAK	0	IRS1
FAK	-1	ALPHAACTININ
FADD	1	SMPD1
FADD	1	NFkB
FADD	1	DEDD1
FADD	1	CASP8

FADD	1	CASP10
FADD	0	TRAF6
FADD	0	TRADD
FADD	0	TNFR1
FADD	0	RIP
FADD	0	ICAD
FADD	0	I-FLICE
FADD	0	FLASH
FADD	0	FAS
FADD	0	FANCF
FADD	-1	MYD88
F7	1	F10
F7	0	F9
F3	0	F9
F3	0	F7
F3	0	F5
F2	1	PAR1
F2	1	Fibrinogen
F13	1	F2
F11	1	F9
F10	0	F7
Estrogen	1	MBD3
Estrogen	1	ER
ErbB3	1	P13K
Era	0	SRC
Era	0	PELP1
ETS2	1	ETS1
ETS2	0	FOS
ETS1	0	ETS2
ERb	-1	Era
ERT	1	GAB2
ERT	1	EF1A
ERT	1	DISHEVELED
ERK5	1	p90RSK
ERK5	1	MEF2
ERK2	1	TCF
ERK2	1	TAU
ERK2	1	STAT2
ERK2	1	SAM68
ERK2	1	RSK2
ERK2	1	RSK
ERK2	1	PLA2P
ERK2	1	PLA2

ERK2	1	PIB5PA
ERK2	1	PDK1
ERK2	1	p90RSK
ERK2	1	MSK2
ERK2	1	MSK1
ERK2	1	MNK2
ERK2	1	MNK1
ERK2	1	MLCK
ERK2	1	MEF2B
ERK2	1	MARCKS
ERK2	1	MAPKAP2
ERK2	1	KV42
ERK2	1	FOS
ERK2	1	ERK1
ERK2	1	ELK1
ERK2	1	EGR1
ERK2	1	EGFR
ERK2	1	CREB
ERK2	1	cPLA2
ERK2	1	CBP
ERK2	1	CARD14
ERK2	1	c-JUN
ERK2	0	KSR
ERK2	-1	SYNAPSIN
ERK2	-1	NFM
ERK2	-1	NFH
ERK2	-1	JNK
ERK2	-1	CASP9
ERK1	1	p90RSK
ERK1	1	MSK2
ERK1	1	MSK1
ERK1	1	MNK2
ERK1	1	MNK1
ERK1	1	MLCK
ERK1	1	ELK1
ERK1	1	cPLA2
ERE	0	Estrogen
ERBIN	1	SMAD3
ERBIN	0	PSD95
ERBIN	0	ITGB4
ERBIN	0	Idax
ERBIN	0	EPS8
ERBIN	0	CD44

ERBIN	0	BPAG1
ERBB2	1	SYNTROPHIN
ERBB2	1	SRC
ERBB2	1	SHC
ERBB2	1	RASGAP
ERBB2	1	PLCy
ERBB2	1	PI3K
ERBB2	1	Grb7
ERBB2	1	GRB2
ERBB2	0	SAP102
ERBB2	0	PSD95
ERBB2	0	PSD93
ERBB2	0	ERBIN
ER	0	Estrogen
ER	0	ERE
EPS8	0	TCF4
EPS8	0	TCF3
EPS8	0	SOX1
EPS8	0	SF1
EPS8	0	SALL1
EPS8	0	RUVBL1
EPS8	0	RNTRE
EPS8	0	GAB2
EPS8	0	FHL2
EPS15	0	REPS2
EPS15	0	GAB2
EPPK1	0	TCF4
EPPK1	0	GAB2
EPOR	1	SHC
EPOR	0	SHP1
EPOR	0	PLCy
EPOR	0	JAK2
EPO	0	IL3
EPO	0	IGF1
EPO	0	EPOR
EPHRIN	1	EPHB2
EPHB2	1	RASGAP
EPHB2	1	DOK
EPAC1	1	RAP1A
EPAC1	1	RAP1
EPAC1	1	GDP
ENKEPHALIN	1	NOPR
ENKEPHALIN	1	MOPR

ENKEPHALIN	1	KOPR
ENKEPHALIN	1	DOPR
ENDOPHILIN	1	RKIP
ENDOPHILIN	1	KIT
ENDOPHILIN	1	DVL2
ENDOPHILIN	1	DAB2
ENDOPHILIN	0	SH3KBP1
ELK4	0	ID3
ELK4	0	ID2
ELK4	0	ID1
ELK3	0	KIT
ELK3	0	ID2
ELK1	1	SRE
ELK1	1	FOS
ELK1	1	c-JUN
ELK1	0	TCF7
ELK1	0	SRF
EIF5	1	EIF2A
EIF4G	0	mRNA
EIF4G	0	CARD14
EIF4F	0	MNK1
EIF4E	0	mRNA
EIF4E	0	EIF4G
EIF4E	0	EIF4F
EIF4B	1	RPS6
EIF4B	1	EIF4A
EIF4B	0	mRNA
EIF4B	0	EIF4E
EIF4A	1	RPS6
EIF4A	0	EIF4G
EIF4A	0	EIF4F
EIF4A	0	EIF4B
EIF4	0	mRNA
EIF4	0	EIF4G
EIF3S1	1	EIF4G
EIF3S1	0	mRNA
EIF3	1	EIF4A
EIF3	0	EIF4G
EIF3	0	EIF4F
EIF3	0	EIF4E
EIF3	0	EIF4A
EIF2G	0	GTP
EIF2G	0	GDP

EIF2B	1	IKKA
EIF2B	1	EIF2A
EIF2B	0	GTP
EIF2B	0	GDP
EIF2B	0	EIF2G
EIF2B	-1	EIF2A
EIF2A	0	tRNA
EIF2A	0	GTP
EIF2A	0	GDP
EIF2A	0	EIF2G
EIF2A	0	EIF2B
EIF-4EBP	1	EIF4E
EIF-4EBP	1	EIF4A
EIF-4EBP	-1	EIF4E
EGR2	1	BNIP3L
EGR2	1	BNIP3
EGR2	1	BAD
EGR2	0	EGR3
EGFR	1	WAVE1
EGFR	1	VAV
EGFR	1	STAT3
EGFR	1	STAT1
EGFR	1	STAT
EGFR	1	SRC
EGFR	1	SHC
EGFR	1	Rho
EGFR	1	RASGAP
EGFR	1	RAC1
EGFR	1	PTK
EGFR	1	PLCy
EGFR	1	p38
EGFR	1	NFAT
EGFR	1	NCK
EGFR	1	N41
EGFR	1	MEKK
EGFR	1	MEK1
EGFR	1	GRB2
EGFR	1	ErbB3
EGFR	1	ERBB2
EGFR	1	CBL
EGFR	1	CASP10
EGFR	0	SRC
EGFR	0	SOS1

EGFR	0	SHC
EGFR	0	PLCy
EGFR	0	PI3K
EGFR	0	JAK1
EGFR	0	GRB2
EGFR	0	GAP
EGFR	0	ERBB2
EGF	1	EGFR
EGF	0	EGFR
EFA6	1	ARF
EF1A	0	ZNF259
EEF2K	-1	EEF2
EDNRA	1	NBS1
EDNRA	0	EDN1
EDG1	0	S1P
EDG1	0	Gy
EDG1	0	Gb
EDG1	0	GA
EDG1	0	G2
ECSIT	1	MEKK
EBS	0	SRF
EBS	0	ELK1
EBP	1	EIF4F
EBP	-1	EIF4E
E3B1	0	EPS8
E3	1	SUMO1
E3	0	G3PD
E2F5	0	JAG1
E2F4	0	p107
E2F1	1	SMAD3
E2F1	1	RB
E2F1	1	Puma
E2F1	1	p14ARF
E2F1	1	Noxa
E2F1	1	Hrk
E2F1	1	DP1
E2F1	1	BIM
E2F1	1	BID
E2F1	0	Ub
E2F1	0	SKP2
E2F1	0	RB
E2F1	-1	RB
E2	0	Ub

E2	0	p53
E2	0	MDM2
E2	0	HIF1A
E2	0	E3
E1A	1	p14ARF
E1A	0	E2
E12	0	NFAT
E12	0	MEF2C
E12	0	HAND2
Dynein	1	RAC1
Dynein	0	NTRK1
Dynein	-1	BIM
Drp-1	0	BAX
Dopamine	1	D3R
Dopamine	1	D2R
Dopamine	1	D2
Dopamine	1	D1R
Dopamine	1	D1
Diversin	0	CSNK1E
DcR3	-1	FASLG
DYRK	1	UBE2D1
DYRK	-1	TAU
DYRK	-1	EIF2A
DYNEIN	0	TUBULIN
DYNAMIN	0	TCF4
DYNAMIN	0	SH3GL3
DYNAMIN	0	PROFILIN
DYNAMIN	0	HOMER
DYNAMIN	0	ENDOPHILIN
DVL3	0	SOCS3
DVL3	0	PPP2CA
DVL3	0	EPS8
DVL2	0	WNT7A
DVL2	0	PRKCG
DVL2	0	FZD4
DSH	0	PP2C
DSH	-1	GSK3B
DSH	-1	GSK3A
DREAM	1	FOS
DREAM	1	CREB
DREAM	-1	KV42
DREAM	-1	KV41
DREAM	-1	Hrk

DREAM	-1	DRE
DREAM	-1	CREB
DR3	1	CASP8
DR3	0	TRADD
DP103	0	METS
DP103	0	MET
DP1	1	CCNE1
DP1	0	E2F1
DOPR	1	GBETAGAMMA
DOPR	1	GALPHAO
DOPR	1	GALPHAI
DOK	1	RASGAP
DOK	0	YES
DOK	0	TCF4
DOK	0	SHIP
DOK	0	RASGAP
DOK	0	PTCH2
DOK	0	p85beta
DOK	0	NCK
DOK	0	KIT
DOK	0	HCK
DOK	0	FGR
DOK	0	FES
DOK	0	ELK3
DOK	0	AIP4
DOCK180	1	AKT
DOC2	1	MUNC13
DNAPK	1	p53
DNAJ	0	NASCENTCHAIN
DLL4	0	NOTCH4
DLL1	0	NOTCH3
DLL1	0	NOTCH2
DLK	1	MKK7
DLK	1	JNKK1
DLC2	-1	Bmf
DKK	0	LRP6
DKK	0	krm
DKK	-1	WNT
DKK	-1	LRP
DJ2	-1	BAX
DJ1	-1	BAX
DISHEVELED	1	PAR1
DISHEVELED	0	VANGL2

DISHEVELED	0	TGIF
DISHEVELED	0	plectin
DISHEVELED	0	N-WASP
DISHEVELED	0	JAG1
DISHEVELED	0	Idax
DISHEVELED	0	HBO1
DISHEVELED	0	HAT1
DISHEVELED	0	DVL3
DISHEVELED	0	CSNK1E
DISHEVELED	0	BP75
DISHEVELED	0	AR
DISHEVELED	-1	AXIN
DIA	1	PFN1
DHPG	1	MGLUR1
DHH	0	PTCH2
DHH	0	PTCH
DHH	0	HHIP
DGK	-1	DAG
DG	1	PKC
DENSIN	0	NMDAR
DELTA	1	SNCA
DELTA	1	p27
DELTA	1	MDM2
DELTA	1	E2F1
DELTA	1	CTNNB1
DELTA	1	CCNE1
DEFCAP	1	CASP9
DEFCAP	1	APAF1
DD	0	ZNF259
DD	0	ID3
DD	0	ID2
DD	0	HIP1
DCP1A	1	WNT
DCP1	0	EIF4G
DCP1	-1	mRNA
DAXX	1	JNKK1
DAXX	1	ASK1
DAXX	0	TNFR1
DAXX	0	RIP
DAXX	0	ICAD
DAXX	0	FAS
DAXX	0	FADD
DAX-1	1	WNT

DAX-1	0	RIP140
DAX-1	0	REA
DAX-1	-1	Era
DARPP-32	1	P35
DARPP-32	-1	PPP1CC
DARPP-32	-1	PP1
DARPP-32	-1	CDK5
DAPK	-1	SYNTAXIN
DAP10	1	VAV
DAP10	1	SLP-76
DAP10	1	PLCy
DAP10	1	PI3K
DAP	0	PSD95
DAG	1	RASGRP1
DAG	1	PKCA
DAG	1	PKC
DAG	1	DG
DAG	0	PLCy
DAG	0	PLCb
DAG	0	PIB5PA
DAB2	1	ID3
DAB2	1	ID2
DAB2	0	ID3
DAB2	0	ID2
DAB2	0	DVL3
DAB1	0	VLDLR
DAAM1	1	AR
DAAM1	0	WNT7A
DAAM1	0	PTCH
DAAM1	0	DVL2
D3R	1	GRB2
D3R	1	GBETAGAMMA
D3R	1	GALPHAO
D3R	1	GALPHAI
D2R	1	GBETAGAMMA
D2R	1	GALPHAZ
D2R	1	GALPHAO
D2R	1	GALPHAI
D2	1	PP2B
D1R	1	GBETAGAMMA
D1R	1	GALPHAS
D1R	1	GALPHAI
D1	1	CAMP

CypA	1	AIF
CryAB	1	CASP3
CryAB	-1	BCL2L1
CryAB	-1	BAX
Cn	1	NFAT
Cn	1	BAD
Clk	1	PER
Cer	-1	WNT
Cd++	1	IP3R
Cd++	1	CREB
Cby	-1	CTNNB1
Cathepsin	-1	BAX
Catalase	1	ROS
Calpastatin	-1	VIL2
Calpastatin	-1	Calpain1
Calpain2	1	TALIN
Calpain2	1	PXN
Calpain2	1	FAK
Calpain2	1	CABIN1
Calpain1	1	VIL2
Calpain1	1	TALIN
Calpain1	1	RHOA
Calpain1	1	RAC1
Calpain1	1	PXN
Calpain1	1	FAK
Calpain1	1	BID
Calpain1	1	BAX
Calpain1	-1	TAU
Calpain1	-1	SPECTRIN
Calpain1	-1	NMDAR
Calpain1	-1	MAP2
Calpain1	-1	CASP9
Calpain1	-1	CABIN1
Calpain1	-1	AMPAR
Ca++	1	VILIP
Ca++	1	SYNAPTOTAGMIN
Ca++	1	SYNAPSIN
Ca++	1	SORCIN
Ca++	1	RASGRP1
Ca++	1	RABPHILIN
Ca++	1	PTK
Ca++	1	PLA2
Ca++	1	PKCA

Ca++	1	PKC
Ca++	1	NOS3
Ca++	1	NFkB
Ca++	1	NFAT
Ca++	1	NCS1
Ca++	1	HIPPOCALCIN
Ca++	1	GCAP
Ca++	1	DYNAMIN
Ca++	1	DREAM
Ca++	1	DOC2
Ca++	1	Cn
Ca++	1	CK1d
Ca++	1	CK1a
Ca++	1	Cd++
Ca++	1	CAPRI
Ca++	1	CAMK2
Ca++	1	CAM
Ca++	1	CALRETICULIN
Ca++	1	Calpain2
Ca++	1	CALNEXIN
Ca++	1	CALMODULIN
Ca++	-1	SV2A
Ca++	-1	PP2A
Ca++	-1	ALPHAACTININ
Ca++	-1	AC5
CYTOCHROMEC	1	APAF1
CYTOCHROMEC	0	SHIP2
CYTOCHROMEC	0	DAB2
CYTOCHROMEC	0	AR
CYCS	1	PER
CYCS	1	cIAP
CYCS	1	CASP1
CYCS	1	APAF1
CXCR4	0	SDF1
CUL1	0	UBE2M
CUL1	0	SKP1
CUL1	0	ROC1
CUL1	0	NEDD8
CTSD	0	E3
CTNND1	0	RAP74
CTNNB1	1	Ub
CTNNB1	1	TCF7
CTNNB1	1	LEF

CTNNB1	1	CBP
CTNNB1	0	Ub
CTNNB1	0	Tsh
CTNNB1	0	TCF
CTNNB1	0	PSEN1
CTNNB1	0	IQGAP
CTNNB1	0	ALPHACATENIN
CTNNB1	-1	TCF
CTNNB1	-1	LEF
CTNNB1	-1	HDAC
CTLA-4	-1	CD3
CTIP	1	BRCA1
CTCF	1	DAAM1
CTBP2	0	TCF4
CTBP1	1	TCF
CTBP1	0	ZEB1
CTBP1	0	TCF
CTBP1	0	SUMO1
CTBP1	0	NOS1
CTBP1	0	GROUCHO
CTBP1	0	CTNNB1
CTBP1	-1	RIP140
CSX	0	SRF
CSX	0	NFAT
CSX	0	HAND2
CSX	0	HAND1
CSX	0	GATA4
CSX	0	E12
CSP	1	GBETAGAMMA
CSP	0	PQCaCh
CSP	-1	NTYPECA
CSNK1E	1	DISHEVELED
CSNK1E	0	DVL3
CSNK1D	1	EPS8
CSL	0	SNW1
CSL	0	SHARP
CSL	0	NOTCH4
CSL	0	NOTCH3
CSL	0	NOTCH2
CSL	0	NICD
CSL	0	NCOR
CSL	0	MAML2
CSL	0	FHL1

CSL	0	CIR
CSK	1	PXN
CSK	1	FAK
CSK	0	PKAc
CSK	0	PAG
CSK	-1	SRC
CSK	-1	LYN
CSK	-1	LCK
CSF2RB	0	KIT
CSF1R	1	p90RSK
CSF1R	1	ETS1
CSF1R	0	M-CSF
CRY	-1	BCL2L1
CRY	-1	BAX
CRMP2	1	TUBULIN
CRM1	0	SMURF1
CRKL	1	DOCK180
CRKL	1	C3G
CRKL	0	P130Cas
CRK	1	MAP4K1
CRK	1	GRIT
CRK	1	DOCK180
CRK	1	C3G
CRK	1	ABL1
CRK	0	SHC
CRK	0	REPS1
CRK	0	PXN
CRK	0	PTK
CRK	0	P130Cas
CRK	0	EGFR
CRIPT	0	TUBULIN
CRIPT	0	PSD95
CREM	0	KIF17B
CREM	0	DREAM
CREM	-1	DREAM
CREM	-1	CREB
CREB	1	TFIID
CREB	1	TFIIB
CREB	1	IRS2
CREB	1	CRE
CREB	1	CBP
CREB	1	ATF1
CREB	0	FOS

CRAC	1	Ca++
CPI17	-1	MLCP
CORTACTIN	0	SHANK
CORTACTIN	0	DYNAMIN
COPS5	1	SMAD4
COMPLEXIN	0	SYNTAXIN
COMPLEXIN	0	SNAP25
COLLAGEN	1	ITGB1
COFILIN2	1	APAF1
COFILIN1	-1	ACTIN
CNTN1	0	NOTCH2
CLTC	1	WNT
CLTC	1	SHP2
CLTC	1	SHIP2
CLTC	1	GRIP1
CLTC	0	STAMBPL1
CLTC	0	KIT
CLOCK	1	BMAL1
CLIP170	0	IQGAP
CLCA2	0	PTCH
CLCA2	0	ITGB4
CLCA2	0	DAAM1
CLCA1	0	ITGB4
CLCA1	0	DAB2
CL	1	BID
CKS1A	0	SKP2
CKS1A	0	SKP1
CKS1A	0	p27
CKS1A	0	FBXO6
CKI	1	DSH
CKI	0	Diversin
CKI	0	AXIN
CKI	-1	CTNNB1
CK2A2	1	EPS8
CK2A2	1	DISHEVELED
CK2	1	WNT
CK2	1	SRF
CK2	1	MAPK9
CK2	1	DSH
CK2	1	BIK
CK2	0	APC
CK2	-1	MAPK9
CK2	-1	BID

CK1e	1	CRY
CK1e	-1	PER
CK1d	1	DARPP-32
CK1a	1	p53
CK1a	1	p25
CK1a	1	NFAT
CK19	-1	CASP9
CITED1	1	GAB2
CITED1	0	TCF4
CITED1	0	Miz1
CITED1	0	KIT
CITED1	0	HSPA8
CITED1	0	FZD2
CITED1	0	ELK3
CIR	0	SAP30
CIR	0	HBO1
CIR	0	HAT1
CIPP	0	NMDAR
CIPP	0	NEUROLIGIN
CIPP	0	NEUREXIN
CIPP	0	KIR41
CHORDIN	0	NOGGIN
CHORDIN	-1	BMPR2
CHOLESTEROL	1	SYNAPTOPHYSIN
CHN1	1	RAC1
CHK2	1	p53
CHK2	1	BRCA1
CHK2	-1	G2
CHK1	1	p53
CHK1	1	CDC25
CHK1	-1	G2
CHK1	-1	CDC25
CHK	0	KIT
CHAPSYN110	0	PSD95
CHAPSYN110	0	NMDAR
CHAPSYN110	0	KIR21
CGMP	1	PKG
CGMP	1	PDE2
CGMP	-1	PDE3B
CGMP	-1	PDE3A
CERAMIDE	1	PPP2R5C
CERAMIDE	1	PPP1CC
CERAMIDE	1	PKCA

CERAMIDE	1	CEK1
CEK1	1	RAF1
CEBPB	0	EF1A
CEBPA	-1	c-JUN
CDK9	1	H3
CDK9	0	GRIP1
CDK7	1	CDK2
CDK7	0	CCNH
CDK6	1	RB
CDK6	-1	BCL2
CDK5	1	TAU
CDK5	1	NFH
CDK5	1	MEK1
CDK5	1	DARPP-32
CDK5	1	APAF1
CDK5	0	SYNTAXIN
CDK5	0	SYNAPSIN
CDK5	-1	PAK1
CDK5	-1	MUNC18
CDK4	0	PCNA
CDK4	0	CDK6
CDK2	1	RB
CDK2	1	p27
CDK2	1	E2F1
CDK2	0	PCNA
CDK1	1	PLK1
CDK1	1	CCNB1
CDK1	1	BAD
CDH1	0	SMAD3
CDC42	1	WAVE1
CDC42	1	THC
CDC42	1	PAK1
CDC42	1	MLK3
CDC42	1	MLK1
CDC42	1	MLK
CDC42	1	MEKK4
CDC42	1	MEKK
CDC42	1	GCK
CDC42	1	ACK
CDC42	0	RAC1
CDC42	0	IQGAP
CDC34	1	E2F1
CDC34	1	CCNE1

CDC34	0	Ub
CDC34	0	SKP1
CDC34	0	CUL1
CDC25C	1	CDK1
CDC25A	1	CDK6
CDC25A	1	CDK2
CDC25	1	CDK1
CDC25	-1	CDK1
CD47	1	BNIP3
CD45	1	LCK
CD44	1	SMAD4
CD44	1	SMAD3
CD44	1	EPS8
CD40	0	TRAF6
CD40	0	TRAF3
CD40	0	CD40L
CD4	1	ZAP70
CD4	0	TCR
CD4	0	LCK
CD4	0	CXCR4
CD36	1	p38
CD36	0	TSP1
CD36	0	FYN
CD3	0	TCR
CD28	1	CD3
CD19	1	VAV
CD19	1	P13K
CD151	0	KIT
CD151	0	ITGA6
CD151	0	FOXH1
CD14	1	TLR4
CD14	1	TLR2
CCR3	0	Gy
CCR3	0	Gb
CCR3	0	GA
CCR3	0	G2
CCR3	0	G1
CCR3	0	CCL11
CCNE1	1	RB
CCNE1	0	Ub
CCNE1	0	PCNA
CCNE1	0	FBW7
CCNE1	0	CDK2

CCND3	1	RB
CCND3	0	CDK6
CCND2	1	RB
CCND2	0	CDK4
CCND1	1	RB
CCND1	1	E2F1
CCND1	0	PPARB
CCND1	0	PCNA
CCND1	0	CDK4
CCND1	0	c-Myc
CCND1	-1	RB
CCNB1	1	CDK1
CCNB1	0	CDK1
CCNA2	1	E2F1
CCNA2	1	CDK2
CCNA2	1	CDK1
CCNA2	0	CDK2
CCNA2	0	CDK1
CCNA2	-1	CDK6
CCNA2	-1	CDK4
CCL5	0	CCR3
CBP	1	SRA1
CBP	1	PCAF
CBP	1	P50
CBP	1	LCK
CBP	1	GROUCHO
CBP	1	Era
CBP	1	ELK1
CBP	1	CTNNB1
CBP	1	c-JUN
CBP	0	TP53BP1
CBP	0	TCF
CBP	0	STAT5
CBP	0	STAT1
CBP	0	SRF
CBP	0	SRC
CBP	0	SMAD4
CBP	0	RELA
CBP	0	PCAF
CBP	0	P50
CBP	0	p300
CBP	0	GROUCHO
CBP	0	EIF4G

CBP	0	CTNNB1
CBP	0	CTBP1
CBP	0	CSK
CBP	-1	TCF7
CBLB	1	GAB2
CBLB	0	Miz1
CBLB	0	KIT
CBLB	-1	VAV2
CBLB	-1	SRC
CBLB	-1	SHC
CBLB	-1	PI3K
CBLB	-1	LCK
CBLB	-1	GRB2
CBLB	-1	FYN
CBLB	-1	EGFR
CBLB	-1	CRK
CBL	0	PLCy
CBL	-1	ZAP70
CBL	-1	FRS2
CBL	-1	FGFR
CBL	-1	EGFR
CBL	-1	CD3
CB2R	1	GBETAGAMMA
CB2R	1	GALPHAO
CB2R	1	GALPHAI
CB1R	1	GBETAGAMMA
CB1R	1	GALPHAO
CB1R	1	GALPHAI
CAVEOLIN	1	FYN
CAVEOLIN	0	SYNTAXIN
CAVEOLIN	0	SNAP25
CAVEOLIN	0	HBO1
CAVEOLIN	0	HAT1
CAVEOLIN	0	Grb7
CAVEOLIN	0	GAB2
CAVEOLIN	0	DYNAMIN
CAVEOLIN	0	AR
CASP9	1	CASP7
CASP9	1	CASP6
CASP9	1	CASP3
CASP9	-1	CASP6
CASP9	-1	CASP3
CASP8	1	MIG1

CASP8	1	IAP
CASP8	1	DEDD1
CASP8	1	CASP3
CASP8	1	BID
CASP8	1	ASK1
CASP8	0	FADD
CASP8	0	DR5
CASP8	0	DR4
CASP8	-1	RIP
CASP8	-1	PROKR1
CASP8	-1	CASP10
CASP8	-1	ASK1
CASP7	1	ICAD
CASP7	1	FAK
CASP7	1	CASP9
CASP7	1	CAD
CASP7	-1	PROKR1
CASP7	-1	PARP
CASP7	-1	MEF2B
CASP7	-1	CAD
CASP6	1	PAK1
CASP6	1	LMNB2
CASP6	1	LMNB1
CASP6	1	LMNA
CASP6	1	ICAD
CASP6	1	CASP3
CASP6	0	CASP7
CASP6	-1	LMNA
CASP3	1	ROCK1
CASP3	1	PAK2
CASP3	1	MEKK
CASP3	1	ICAD
CASP3	1	GELSOLIN
CASP3	1	GAS2
CASP3	1	FODRIN
CASP3	1	CASP8
CASP3	1	CASP7
CASP3	1	CASP6
CASP3	1	CALCINEURIN
CASP3	1	CAD
CASP3	1	BIM
CASP3	1	BID
CASP3	1	BAX

CASP3	1	BAD
CASP3	1	APAF1
CASP3	1	Acinus
CASP3	0	plectin
CASP3	0	CASP7
CASP3	-1	XIAP
CASP3	-1	RB
CASP3	-1	PROKR1
CASP3	-1	PPP2R5C
CASP3	-1	PIP5K
CASP3	-1	PARP
CASP3	-1	NDUFS1
CASP3	-1	MEF2B
CASP3	-1	MCL1
CASP3	-1	ICAD
CASP3	-1	GELSOLIN
CASP3	-1	FODRIN
CASP3	-1	FAK
CASP3	-1	EIF4G
CASP3	-1	DNAPK
CASP3	-1	BCL2L1
CASP3	-1	BCL2
CASP3	-1	APAF1
CASP2	1	LMNA
CASP2	1	BID
CASP10	1	p53
CASP10	1	IAP
CASP10	1	FANCF
CASP10	1	CASP3
CASP10	1	APAF1
CASP10	0	FADD
CASP10	0	DR5
CASP10	0	DR4
CASP10	0	CASP8
CASK	1	TBR1
CASK	1	TAU
CASK	1	SYNTAXIN
CASK	1	PMCA
CASK	1	INHIBITOR2
CASK	1	DISHEVELED
CASK	0	MINT
CASK	0	CPI17
CASK	0	CASKIN

CASK	-1	NFAT
CASK	-1	ANKYRIN
CARM1	1	p300
CARM1	1	CTNNB1
CARM1	1	CBP
CARM1	0	RXR
CARM1	0	RAR
CARD14	1	EIF4E
CAPRI	-1	RAS
CAP	1	MYT1
CAP	-1	MYT1
CAMP	1	RIIa
CAMP	1	PKAc
CAMP	1	PKA
CAMP	1	EPAC1
CAMP	1	CNG
CAMP	1	cAMPGEFII
CAMP	-1	PKAc
CAMKPASE	-1	CAMKIV
CAMKPASE	-1	CAMK2A
CAMKPASE	-1	CAMK1
CAMKK	1	CAMKIV
CAMKK	1	CAMK1
CAMKK	1	AKT
CAMKIV	1	SYNAPSIN
CAMKIV	1	CREM
CAMKIV	1	CREB
CAMKIV	-1	NOS1
CAMK2A	1	TIAM1
CAMK2A	1	TAK1
CAMK2A	1	SYNAPTOTAGMIN
CAMK2A	1	SAP97
CAMK2A	1	RYR
CAMK2A	1	RASGRF
CAMK2A	1	RABPHILIN
CAMK2A	1	NTYPECA
CAMK2A	1	MAP2
CAMK2A	1	KV42
CAMK2A	1	GAB2
CAMK2A	1	CREB
CAMK2A	1	AMPAR
CAMK2A	0	VAMP
CAMK2A	0	RNTRE

CAMK2A	0	PSMD4
CAMK2A	0	PPP2R5B
CAMK2A	0	PPP2CA
CAMK2A	0	NMDAR
CAMK2A	0	MEKK4
CAMK2A	0	LRP5
CAMK2A	0	EPS8
CAMK2A	0	DVL3
CAMK2A	0	DVL2
CAMK2A	0	DISHEVELED
CAMK2A	0	DENSIN
CAMK2A	0	DAB2
CAMK2A	0	CSNK1E
CAMK2A	0	CD44
CAMK2A	0	AXAM2
CAMK2A	0	ARNIP
CAMK2A	0	AML3
CAMK2A	-1	SYNTAXIN
CAMK2A	-1	SYNGAP
CAMK2A	-1	SYNAPSIN
CAMK2A	-1	SPINOPHILIN
CAMK2A	-1	RICS
CAMK2A	-1	PPP2R5C
CAMK2A	-1	NOS1
CAMK2	1	TAU
CAMK2	1	CREB
CAMK2	0	IL1A
CAMK1	1	RAC1
CAMK1	1	MITR
CAMK1	1	MEF2C
CAMK1	1	HDAC5
CAMK1	1	CREB
CAMK1	0	CAMKIV
CAMK1	-1	SYNAPSIN
CAM	1	PTK
CAM	1	PPP3R2
CAM	1	NFAT
CAM	1	CAMK1
CAM	0	NOS3
CAM	0	NFAT
CAM	0	CAMK2
CAM	-1	CABIN1
CALRETICULIN	0	NASCENTCHAIN

CALRETICULIN	0	AR
CALNEXIN	0	NASCENTCHAIN
CALMODULIN	1	RYR
CALMODULIN	1	RIN1
CALMODULIN	1	RIN
CALMODULIN	1	RIC
CALMODULIN	1	RASGRF
CALMODULIN	1	PMCA
CALMODULIN	1	PLCb
CALMODULIN	1	PDE1C
CALMODULIN	1	PDE1B
CALMODULIN	1	PDE1A
CALMODULIN	1	P35611
CALMODULIN	1	NOS1
CALMODULIN	1	MUNC13
CALMODULIN	1	MLCK
CALMODULIN	1	IQGAP
CALMODULIN	1	EEF2K
CALMODULIN	1	DAPK
CALMODULIN	1	CAMKK
CALMODULIN	1	CAMKIV
CALMODULIN	1	CAMK2A
CALMODULIN	1	CALCINEURIN
CALMODULIN	1	ADDUCIN
CALMODULIN	1	AC1
CALMODULIN	0	MYOSINV
CALMODULIN	0	CLATHRIN
CALMODULIN	-1	VAMP
CALMODULIN	-1	SPECTRIN
CALMODULIN	-1	RAS
CALMODULIN	-1	RAB3
CALMODULIN	-1	NMDAR
CALMODULIN	-1	MGLUR7
CALMODULIN	-1	MARCKS
CALMODULIN	-1	IP3R
CALMODULIN	-1	D2R
CALMODULIN	-1	bARK
CALMODULIN	-1	AKAP
CALM3	1	PKC
CALM3	1	IP3R
CALM3	1	Ca++
CALM3	1	AKT
CALCINEURIN	1	STEP

CALCINEURIN	1	NOS1
CALCINEURIN	1	NFAT
CALCINEURIN	1	IP3R
CALCINEURIN	1	GABAAR
CALCINEURIN	1	BAD
CALCINEURIN	-1	TUBULIN
CALCINEURIN	-1	TAU
CALCINEURIN	-1	SYNAPSIN
CALCINEURIN	-1	NMDAR
CALCINEURIN	-1	MAP2
CALCINEURIN	-1	LTYPECA
CALCINEURIN	-1	I1
CALCINEURIN	-1	DARPP-32
CALCINEURIN	-1	CREB
CALCINEURIN	-1	CAMKIV
CALBRAIN	-1	CAMK2A
CAD	1	ICAD
CAD	0	TopoII
CAD	0	H1
CABIN1	0	PPP3R2
CABIN1	0	MEF2D
CABIN1	0	CAM
CABIN1	-1	HDAC5
CABIN1	-1	CALCINEURIN
C3G	1	RAS
C3G	1	RAP1A
C3G	1	RAP1
Bok	1	CYTOCHROMEC
Bmf	-1	MCL1
Bmf	-1	BCL2L1
Bmf	-1	BCL2
Bmf	-1	Bcl-w
Bcl-w	-1	BIM
Bcl-w	-1	BIK
Bcl-w	-1	BID
Bcl-w	-1	BAX
Bcl-w	-1	BAK
Bcl-w	-1	BAD
Bcl-rambo	1	CYTOCHROMEC
Bcl-b	-1	BAX
BTRC	1	CTNNB1
BTRC	0	CTNNB1
BTRC	-1	CTNNB1

BTK	1	PLCy
BTK	1	PLCg-2
BTK	-1	FAS
BRCA2	0	RAD51
BRCA1	0	CTIP
BRADYKININ	1	BR1R
BR1R	1	Gia
BR1R	1	GBETAGAMMA
BR1R	1	GAQ
BR1R	1	GALPHAI
BPAG2	0	ITGB4
BPAG2	0	ITGA6
BPAG2	0	DSP
BPAG1	1	WNT2
BPAG1	1	RANBP9
BPAG1	0	ITGB4
BPAG1	0	ID3
BPAG1	0	BPAG2
BNIP3L	-1	BCL2L1
BNIP3L	-1	BCL2
BNIP3	-1	BCL2L1
BNIP3	-1	BCL2
BMPR2	1	ATF2
BMPR2	0	BMP-4
BMPR2	0	BMP-2
BMP-7	0	BMPR1
BMP-5	0	BMPR1
BMP-10	0	BMPR1
BMAL1	0	CLOCK
BLNK	1	VAV
BLNK	1	PLCg-2
BLNK	1	GRB2
BLNK	0	VAV
BLNK	0	SYK
BLNK	0	PLCy
BLNK	0	LYN
BLNK	0	BTK
BIRC5	1	XIAP
BIRC5	1	TUBB4Q
BIRC5	-1	cIAP
BIP	1	SEC61P
BIP	0	NASCENTCHAIN
BIM	1	BAX

BIM	1	BAK
BIM	-1	MCL1
BIM	-1	BCL2L1
BIM	-1	BCL2
BIM	-1	Bcl-w
BIK	0	Bcl-w
BIK	-1	BCL2L1
BIK	-1	BCL2
BID	1	CYTOCHROMEC
BID	1	BAX
BID	1	BAK
BID	0	CKI
BID	-1	VDAC2
BI-1	-1	BAX
BFAR	-1	CASP8
BETAARRESTIN	1	SRC
BETAARRESTIN	1	RHOA
BETAARRESTIN	1	RAF1
BETAARRESTIN	1	PI3K
BETAARRESTIN	1	PDE4D
BETAARRESTIN	1	PDE
BETAARRESTIN	1	p38
BETAARRESTIN	1	MEK1
BETAARRESTIN	1	JNK3
BETAARRESTIN	1	IKBA
BETAARRESTIN	1	FRIZZLED
BETAARRESTIN	1	DSH
BETAARRESTIN	1	ASK1
BETAARRESTIN	1	AGTR1
BETAARRESTIN	0	DVL2
BETAARRESTIN	0	DISHEVELED
BETAARRESTIN	0	CLATHRIN
BETAARRESTIN	-1	PAR2
BETAARRESTIN	-1	MOR
BETAARRESTIN	-1	JNK3
BETAARRESTIN	-1	IRS1
BETAARRESTIN	-1	GA
BETAARRESTIN	-1	B2AR
BETAARRESTIN	-1	AR
BDNF	1	TRKB
BCR	1	SYK
BCL2L1	1	BIK
BCL2L1	1	BCL2

BCL2L1	0	BIK
BCL2L1	0	APAF1
BCL2L1	-1	p53
BCL2L1	-1	Noxa
BCL2L1	-1	CYTOCHROMEC
BCL2L1	-1	CYCS
BCL2L1	-1	Bok
BCL2L1	-1	BIM
BCL2L1	-1	BIK
BCL2L1	-1	BID
BCL2L1	-1	BCLG
BCL2L1	-1	BAX
BCL2L1	-1	BAK
BCL2L1	-1	BAD
BCL2	0	BIK
BCL2	0	BCL2L1
BCL2	-1	SMPD1
BCL2	-1	Noxa
BCL2	-1	ITM2B
BCL2	-1	CYTOCHROMEC
BCL2	-1	BIM
BCL2	-1	BIK
BCL2	-1	BID
BCL2	-1	BCL2L1
BCL2	-1	BAX
BCL2	-1	BAK
BCL2	-1	BAD
BCL2	-1	APAF1
BAX	1	CYTOCHROMEC
BAX	1	cIAP
BAX	1	BCL2
BAX	1	BAK
BAX	0	BCL2L1
BAX	0	BCL2
BAX	-1	BCL2
BAX	-1	BAD
BAP31	1	BCL2L1
BAP31	1	BCL2
BAP31	-1	CASP8
BAK	1	CYTOCHROMEC
BAK	1	cIAP
BAK	1	BCL2L1
BAK	0	BAX

BAK	-1	BAD
BAG1	1	RAF1
BAG1	1	BCL2
BAD	1	BCL2L1
BAD	1	BCL2
BAD	1	BAX
BAD	1	BAK
BAD	0	BCL2L1
BAD	0	BCL2
BAD	0	BAX
BAD	-1	CASP9
BAD	-1	BCL2L1
BAD	-1	BCL2
BAD	-1	Bcl-w
B2AR	1	GBETAGAMMA
B2AR	1	GALPHAS
B2AR	0	NHERF
B2AR	0	GA
Aven	-1	APAF1
Akt	1	XIAP
Akt	1	PDE3B
Akt	-1	TSC1
Akt	-1	GSK3A
Akt	-1	CASP9
Akt	-1	BAX
Ab-R	1	TAU
Ab-R	1	GSK3B
Ab-R	1	D4-GDI
AXIN2	0	RNTRE
AXIN2	0	EPS8
AXIN2	0	CSNK1E
AXIN	1	MEKK4
AXIN	1	MEKK
AXIN	1	GSK3A
AXIN	0	DSH
AXIN	0	Diversin
AXIN	0	CTNNB1
AXIN	0	ANAPC1
AXIN	0	ALPHACATENIN
AXIN	-1	CTNNB1
AXAM2	1	TCF4
AVP	1	Ca++
ATR	1	RAD17

ATR	1	PLK3
ATR	1	p53
ATR	1	FANCD2
ATR	1	CHK2
ATR	1	CHK1
ATR	1	BRCA1
ATR	1	ATM
ATR	0	ATRIP
ATP	1	CASP9
ATP	1	CAMP
ATP	-1	CAMP
ATM	1	p53
ATM	1	NFkB
ATM	1	NBS1
ATM	1	FANCD2
ATM	1	CHK2
ATM	1	CHK1
ATM	1	BRCA2
ATM	1	BRCA1
ATM	1	ABL1
ATM	0	ATR
ATM	-1	p53
ATF3	0	ID2
ATF2	1	TRE
ATF2	1	IL4
ATF2	1	IL2
ATF2	1	IFNy
ATF2	1	CRE
ATF2	1	c-JUN
ATF2	0	TREH
ATF2	0	GATA4
ATF2	0	CSX
ATF2	0	c-JUN
ASK1	1	MKK7
ASK1	1	MEK6
ASK1	1	MEK3
ASK1	1	JNKK1
ASK1	1	JNK
ASK1	0	TRAF2
ASK1	0	DAXX
ASC	1	BAX
ASAP1	1	AR
ASAP1	0	REPS2

ASAP1	0	EF1A
ASAP1	-1	p14ARF
ARTS	-1	XIAP
ARRB2	0	FZD4
ARRB2	0	DVL2
ARP3	0	p34
ARP3	0	P21
ARP23	1	ACTIN
ARP2	1	ACTIN
ARP2	0	IL9
ARP2	0	ARP3
ARNT	1	AHR
ARNT	0	HIF1A
ARNO	1	ARF
ARNIP	1	AR
ARNIP	0	EPS8
ARNIP	0	AXIN2
ARHGEF6	1	PAK1
ARHGEF6	1	CDC42
ARHGEF6	1	AKT
ARHGEF5	1	RHOA
ARHGEF11	1	RHOA
ARHGEF1	1	RHOA
ARHGEF1	1	ARHGAP1
ARHGEF1	0	JIP
ARHGAP6	1	RHOA
ARHGAP5	1	RHOA
ARHGAP5	-1	Rho
ARHGAP4	1	RHOA
ARHGAP1	1	ROCK2
ARHGAP1	1	RHOA
ARHGAP1	1	PIP5K
ARHGAP1	1	CITRON
ARHGAP1	0	PKCA
ARFGEF	1	ARF
ARFGAP	-1	ARF
ARF4	0	GAB2
ARF1	1	PLCD1
ARF1	1	PKCE
ARF	0	PICK1
ARAI	0	IBP
ARAD	0	DAP10
AR	1	UBC9

AR	1	STAT1
AR	1	MST1R
AR	1	Gia
AR	1	Gai
AR	1	FORKHEAD
AR	1	ASAP1
AR	0	WNT2
AR	0	UXT
AR	0	UBE3A
AR	0	TR4
AR	0	TNIP1
AR	0	TGIF
AR	0	TFIIH
AR	0	TBPIP
AR	0	SVIL
AR	0	STUB1
AR	0	SRY
AR	0	SPDEF
AR	0	SLC25A4
AR	0	SHP
AR	0	SF1
AR	0	RNF4
AR	0	RIP140
AR	0	RAP74
AR	0	RAP30
AR	0	RANBP9
AR	0	Rad54l2
AR	0	RAC3
AR	0	PLAGL1
AR	0	PIAS4
AR	0	PIAS3
AR	0	PIAS
AR	0	PATZ
AR	0	PAK6
AR	0	NSD1
AR	0	Miz1
AR	0	KPNB1
AR	0	HBO1
AR	0	GRIP1
AR	0	GP130
AR	0	GAB2
AR	0	FZD2
AR	0	FHL2

AR	0	ERM
AR	0	EPS8
AR	0	EPPK1
AR	0	EBP1
AR	0	DD
AR	0	CLTC
AR	0	CDK9
AR	0	CDC37
AR	0	BPAG2
AR	0	BAG1
AR	0	ARNIP
AR	0	ARA70
AR	0	ARA55
AR	0	ARA54
AR	0	ARA160
AR	0	APPL
AR	0	AP2A1
AR	0	AML3
AR	0	AML1
AR	0	AES
AR	0	37530
AR	0	37165
APS	1	VAV2
APS	1	CBL
APS	0	KIT
APPL2	0	RBBP7
APPL2	0	RAB5
APPL2	0	MTA2
APPL	0	RBBP7
APPL	0	RAB5
APPL	0	MTA2
APP	0	NUMBL
APP	0	NUMB
APOLLON	-1	Omi
APOLLON	-1	cIAP
APOLLON	-1	CASP9
APOER2	0	DAB1
APO3L	0	DR3
APO2L	0	DR5
APO2L	0	DR4
APLP1	1	Ub
APIP	-1	APAF1
APE1	0	PP32

APE1	0	NME1
APC	1	AXIN
APC	0	SMAD4
APC	0	SMAD3
APC	0	GSK3A
APC	0	GPCR
APC	0	CTNNB1
APC	0	CTBP1
APC	0	CDH1
APC	0	AXIN
APC	0	ALPHACATENIN
APC	-1	CTNNB1
APAF1	1	MAL
APAF1	1	CYCS
APAF1	1	CASP9
APAF1	0	WAVE3
APAF1	0	WAVE2
APAF1	0	WAVE1
APAF1	0	TALIN
APAF1	0	PXN
APAF1	0	CYCS
APAF1	0	CASP9
APAF1	0	BCL2L1
APAF1	0	BCL2
APAF1	0	ATP
AP2A1	0	EPS15
AP2	0	SYNAPTOTAGMIN
AP2	0	L1
AP2	0	EPS8
AP2	0	CLATHRIN
AP2	0	BETAARRESTIN
AP2	0	ARNIP
ANKYRIN	0	SPECTRIN
ANKYRIN	0	NRCAM
ANKYRIN	0	L1
ANKYRIN	0	FASCIN
ANF	1	pGC
ANAPC2	0	FZR1
ANAPC1	-1	CTNNB1
ANANDAMIDE	1	CB2R
ANANDAMIDE	1	CB1R
AMPK	1	P21
AMPK	1	ACC

AMPHIPHYSIN	0	DYNAMIN
AMPAR	1	SODIUM
AMPAR	0	SYNTENIN
AMPAR	0	STARGAZIN
AMPAR	0	SAP97
AMPAR	0	PICK1
AMPAR	0	HOMER
AMPAR	0	GRIP
AMPAR	0	AP2
AMP	1	AMPK
AML3	0	DVL2
AMISYN	-1	SYNTAXIN
AMISYN	-1	SNAP25
ALPHAACTININ	0	RABPHILIN
ALPHAACTININ	0	PSD95
ALPHAACTININ	0	PLD
ALPHAACTININ	0	NMDAR
ALPHAACTININ	0	DENSIN
ALPHAACTININ	0	CAMK2A
ALPHA7NACHR	1	SODIUM
ALPHA2AR	1	GBETAGAMMA
ALPHA2AR	1	GALPHAO
ALPHA2AR	1	GALPHAI
ALPHA1AR	1	Gia
ALPHA1AR	1	GBETAGAMMA
ALPHA1AR	1	GAQ
ALK3	1	ATF2
ALK3	0	BMPR2
ALK3	0	BMPR1
ALK3	0	BMP-7
ALK3	0	BMP-5
ALK3	0	BMP-4
ALK3	0	BMP-2
ALK3	0	BMP-10
ALDOA	0	CTSD
AKT	1	TSC1
AKT	1	TERT
AKT	1	STAT3
AKT	1	RAF1
AKT	1	RAC1
AKT	1	PAK1
AKT	1	NOS3
AKT	1	MTOR

AKT	1	MLK3
AKT	1	MLK1
AKT	1	MLK
AKT	1	MEKK4
AKT	1	MEKK
AKT	1	MDM2
AKT	1	IKKy
AKT	1	IKKA
AKT	1	IKK2
AKT	1	Hsp90
AKT	1	Gy
AKT	1	GSK3B
AKT	1	GR
AKT	1	GCK
AKT	1	FKHRL1
AKT	1	CREB
AKT	1	CASP9
AKT	1	BAD
AKT	1	ACTIN
AKT	0	IQGAP
AKT	0	Hsp90
AKT	-1	TP53BP1
AKT	-1	SOD
AKT	-1	RAF1
AKT	-1	GSK3B
AKT	-1	FORKHEAD
AKT	-1	BAD
AKT	-1	AFX
AKAP15	0	PKA
AKAP15	0	LTYPECA
AKAP13	1	RHOA
AKAP13	0	RIIa
AKAP	0	PSD95
AKAP	0	PKA
AKAP	0	CALCINEURIN
AIP4	1	KIT
AIP4	1	ENDOPHILIN
AIP4	0	SNW1
AIP4	0	NUMB
AHR	0	XAP2
AHR	0	Hsp90
AHR	0	ARNT
AGTR2	1	NBS1

AGTR2	0	SHC
AGTR2	0	Gy
AGTR2	0	Gia
AGTR2	0	Gb
AGTR2	0	G1
AGTR2	0	AGT
AGTR1	1	JAK1
AGTR1	1	Ca++
AGTR1	0	GAQ
AFX	1	FAS
ADPRIBSYCYCLASE	1	cADPR
ADENOSINE	1	A2AR
ADENOSINE	1	A1R
ADDUCIN	0	ID3
ADDUCIN	0	ID2
ADAPTIN	1	DYNAMIN
ADAPTIN	1	CLATHRIN
ADAPTIN	0	SYNAPTOPHYSIN
ADAPTIN	-1	GABAAR
ADAM17	1	NICD
ADAM12	1	EGF
ADAM10	1	DLL1
AChE	1	CYTOCHROMEC
AChE	1	APAF1
ACTR	0	RXR
ACTR	0	p300
ACTR	0	CBP
ACTR	0	CARM1
ACTN1	1	ACTIN
ACTN1	0	Zyxin
ACTN1	0	VCL
ACTN1	0	TALIN
ACTN1	0	SPTAN1
ACTN1	0	APAF1
ACTIN	0	SPINOPHILIN
ACTIN	0	SPECTRIN
ACTIN	0	RADIXIN
ACTIN	0	NEURABIN
ACTIN	0	N41
ACTIN	0	IQGAP
ACTIN	0	FILAMIN
ACTIN	0	FASCIN
ACTIN	0	CORTACTIN

ACTIN	0	ALPHAACTININ
ACTG	1	APAF1
ACTG	0	WAVE3
ACTG	0	WAVE2
ACTG	0	WAVE1
ACTG	0	TALIN
ACTG	0	ACTN1
ACT	0	CREM
ACK	1	MCF2
ACK	0	VCL
ACK	0	TALIN
ACK	0	Miz1
ACK	0	CTNND1
ACHRE	1	CALM3
ACH	1	M4R
ACH	1	M2R
ACH	1	M1R
ACH	1	ALPHA7NACHR
AC5	1	CAMP
AC2	1	CAMP
AC1	1	CAMP
AC	1	GAS
AC	1	CAMP
AC	1	ATP
AC	0	SPHK1
AC	0	SMPD1
AC	0	RAC1
AC	0	GPCR
AC	0	CAP
ABP1	0	PICCOLO
ABP1	0	DYNAMIN
ABL1	1	VAV2
ABL1	1	TP53BP1
ABL1	1	RAD9
ABL1	1	P73
ABL1	1	p14ARF
ABL1	1	JNK
ABL1	1	DOK
ABL1	1	CDK5
ABL1	0	SHC
ABL1	0	RB
ABL1	0	PXN
ABL1	0	GRB2

ABL1	-1	RAD51
ABL1	-1	CRK
ABI2	0	WAVE3
ABI2	0	WAVE2
ABI2	0	WAVE1
ABI2	0	RAC1
ABI2	0	PIR
ABI2	0	NAP
AA	1	PKCA
A2AR	1	GBETAGAMMA
A2AR	1	GALPHAS
A20	0	TNF
A20	0	CAMK2
A20	-1	TRAF6
A20	-1	TRAF2
A20	-1	MKP1
A1R	1	GBETAGAMMA
A1R	1	GALPHAI
A-Raf	1	UBE3A
A-Raf	1	MEK1
A-Raf	0	MEK2
7TMR	1	BETAARRESTIN
4-1BB	0	TRAF2
4-1BB	0	4-1BBL
14-3-3	1	FKHRL1
14-3-3	1	BCL2
14-3-3	1	BAD
14-3-3	0	MITR
14-3-3	0	HDAC5
14-3-3	0	FKHRL1
14-3-3	0	CDC25C
14-3-3	0	CDC25
14-3-3	0	BAD
14-3-3	-1	FORKHEAD
14-3-3	-1	BAX
14-3-3	-1	BAD

Table S4. Information of drug-targets and side-effects for genes in HSN.

GeneName	Drug-target (1:True,0:False)	Side-effect (1:True,0:False)
37165	0	0
37530	0	0
14-3-3	0	0
4-1BB	0	0
4-1BBL	0	0
7TMR	0	0
A1R	1	0
A20	0	0
a2-Antiplasmin	0	0
A2AR	1	0
AA	0	0
ABI2	0	0
ABL1	1	1
ABP1	1	0
Ab-R	0	0
AC	0	0
AC1	1	0
AC2	1	0
AC5	1	0
ACC	1	0
ACH	1	0
AChE	1	1
ACHRE	1	0
Acinus	0	0
ACK	1	0
ACT	0	0
ACTG	0	0
ACTIN	0	0
ACTN1	0	0
ACTR	0	0
ACVRL1	1	0
ADAM10	1	0
ADAM12	0	0
ADAM17	1	0
ADAPTIN	0	0
ADDUCIN	0	0
ADENOSINE	0	0
ADPRIBSYCYCLASE	0	0

AES	0	0
AFX	0	0
AGT	1	0
AGTR1	1	1
AGTR2	1	0
AHR	1	0
AIF	1	0
AIP4	0	0
AKAP	0	0
AKAP13	0	0
AKAP15	0	0
Akkt	1	0
AKT	1	0
ALDOA	1	0
Alg-2	0	0
ALK3	0	0
ALPHA1AR	1	0
ALPHA2AR	1	0
ALPHA7NACHR	1	0
ALPHAACTININ	0	0
ALPHACATENIN	0	0
AMISYN	0	0
AML1	0	0
AML3	0	0
AMP	1	0
AMPAR	1	0
AMPHIPHYSIN	0	0
AMPK	1	0
ANANDAMIDE	0	0
ANAPC1	0	0
ANAPC2	0	0
ANF	0	0
ANKYRIN	0	0
AP2	0	0
AP2A1	0	0
AP2B1	0	0
APAF1	1	0
APC	1	0
APE1	1	0
APIP	0	0
APLP1	0	0

APO2L	0	0
APO3L	0	0
APOER2	0	0
APOLLON	0	0
APP	1	0
APPL	0	0
APPL2	0	0
APS	0	0
AR	1	0
ARA160	0	0
ARA54	0	0
ARA55	0	0
ARA70	0	0
ARAD	0	0
A-Raf	1	0
ARAI	0	0
ARF	0	0
ARF1	1	0
ARF4	1	0
ARFGAP	0	0
ARFGEF	0	0
ARHGAP1	0	0
ARHGAP4	0	0
ARHGAP5	0	0
ARHGAP6	0	0
ARHGEF1	0	0
ARHGEF11	0	0
ARHGEF5	0	0
ARHGEF6	0	0
ARIP2	0	0
ARNIP	0	0
ARNO	1	0
ARNT	0	0
ARP2	1	0
ARP23	1	0
ARP3	1	0
ARRB2	0	0
ARTS	0	0
ASAP1	0	0
ASC	0	0
ASCL1	0	0

ASK1	0	0
ATF1	1	0
ATF2	1	0
ATF3	1	0
ATM	1	0
ATP	0	0
ATR	0	0
ATRIP	0	0
Aven	0	0
AVP	0	0
AXAM2	0	0
AXIN	1	0
AXIN2	0	0
B2AR	1	0
BAD	1	0
BAG1	1	0
BAK	1	0
BAP31	0	0
bARK	1	0
BAX	0	0
BCL10	0	0
BCL2	1	0
BCL2L1	1	0
Bcl-b	0	0
BCLG	0	0
Bcl-rambo	0	0
Bcl-w	0	0
BCR	1	1
BDNF	1	0
BETAARRESTIN	0	0
BFAR	0	0
BI-1	0	0
BID	0	0
BIK	0	0
BIM	0	0
BIP	1	0
BIRC5	1	0
BLNK	0	0
BMAL1	0	0
Bmf	0	0
BMP-10	0	0

BMP-2	0	0
BMP-4	0	0
BMP-5	0	0
BMP-7	0	0
BMPR1	0	0
BMPR2	0	0
BNIP3	0	0
BNIP3L	0	0
Bok	0	0
BP75	0	0
BPAG1	0	0
BPAG2	0	0
BR1R	0	0
BRADYKININ	0	0
bRAF	1	0
BRCA1	0	0
BRCA2	0	0
BTK	1	0
BTRC	0	0
C3G	0	0
Ca++	0	0
CABIN1	0	0
CAD	1	0
cADPR	0	0
CALBRAIN	0	0
CALCINEURIN	0	0
CALM3	0	0
CALMODULIN	1	0
CALNEXIN	1	0
Calpain1	1	0
Calpain2	0	0
Calpastatin	1	0
CALRETICULIN	1	0
CAM	0	0
CAMK	1	0
CAMK1	0	0
CAMK2	1	0
CAMK2A	1	0
CAMKIV	1	0
CAMKK	0	0
CAMKPASE	0	0

CAMP	1	0
cAMPGEFII	0	0
CAP	0	0
CAPRI	0	0
cAR	1	0
CARD14	0	0
CARM1	0	0
CASK	1	0
CASKIN	0	0
CASP1	1	0
CASP10	0	0
CASP2	0	0
CASP3	1	0
CASP6	0	0
CASP7	1	0
CASP8	0	0
CASP9	0	0
Catalase	1	0
Cathepsin	1	0
CAV2	0	0
CAVEOLIN	0	0
CB1R	1	0
CB2R	1	0
CBL	0	0
CBLB	0	0
CBP	1	0
Cby	0	0
CCL11	1	0
CCL5	1	0
CCNA2	1	0
CCNB1	0	0
CCNB2	0	0
CCND1	1	0
CCND2	0	0
CCND3	0	0
CCNE1	0	0
CCNH	0	0
CCR3	0	0
CCR5	1	0
CCT4	0	0
Cd++	0	0

CD14	0	0
CD151	0	0
CD19	1	0
CD28	0	0
CD3	1	0
CD36	0	0
CD4	1	0
CD40	0	0
CD40L	0	0
CD44	1	0
CD45	0	0
CD47	0	0
CDC16	0	0
CDC25	0	0
CDC25A	0	0
CDC25C	0	0
CDC27	0	0
CDC34	0	0
CDC37	0	0
CDC42	1	0
CDH1	0	0
CDK1	1	0
CDK2	1	0
CDK4	1	0
CDK5	1	0
CDK6	1	0
CDK7	1	0
CDK9	1	0
CDX1	0	0
CEACAM1	1	0
CEBPA	0	0
CEBPB	0	0
CEK1	0	0
CEM15	0	0
Cer	0	0
CERAMIDE	0	0
CGMP	0	0
CHAPSYN110	0	0
CHK	0	0
CHK1	1	0
CHK2	1	0

CHLORIDE	0	0
CHN1	0	0
CHOLESTEROL	0	0
CHOP10	0	0
CHORDIN	0	0
CHREBP	0	0
cIAP	0	0
cIAP1	0	0
cIAP2	0	0
CIPP	0	0
CIR	1	0
CITED1	0	0
CITRON	0	0
c-JUN	1	0
CK19	0	0
CK1a	0	0
CK1d	1	0
CK1e	1	0
CK2	1	0
CK2A2	1	0
CKI	1	0
CKS1A	0	0
CL	0	0
CLATHRIN	0	0
CLCA1	0	0
CLCA2	0	0
CLIP170	0	0
Clk	1	0
CLOCK	0	0
CLTC	0	0
cMAF	0	0
c-Myc	0	0
Cn	0	0
CNG	0	0
CNTN1	0	0
COFILIN1	1	0
COFILIN2	0	0
COLLAGEN	1	0
COMPLEXIN	0	0
COPS5	0	0
CORTACTIN	0	0

CPI	0	0
CPI17	0	0
cPLA2	0	0
CRAC	0	0
CRE	0	0
CREB	1	0
CREM	0	0
CRI2	0	0
CRIPT	0	0
CRK	0	0
CRKL	0	0
CRM1	0	0
CRMP2	0	0
CRY	1	0
CryAB	0	0
CSF1R	1	0
CSF2RB	1	0
CSK	1	0
CSL	0	0
CSNK1D	0	0
CSNK1E	0	0
CSP	0	0
CSX	0	0
CTBP1	1	0
CTBP2	0	0
CTCF	0	0
CTIP	0	0
CTLA-4	1	0
CTNNB1	1	0
CTNND1	0	0
CTSD	1	0
CUL1	0	0
CXCR4	1	0
CYCS	1	0
CYP19A1	1	1
CypA	1	0
CYTOCHROMEC	1	0
D1	0	0
D1R	1	0
D2	0	0
D2R	1	0

D3R	1	1
D4-GDI	0	0
DAAM1	0	0
DAB1	0	0
DAB2	0	0
DAG	0	0
DAP	0	0
DAP10	0	0
DAPK	1	0
DARPP-32	0	0
dATP	0	0
DAX-1	1	0
DAXX	0	0
DCP1	1	1
DCP1A	0	0
DcR3	0	0
DD	1	0
DEDD1	0	0
DEFCAP	0	0
DELTA	1	0
DENSIN	0	0
DFFB	0	0
DG	0	0
DGK	0	0
DHH	0	0
DHPG	0	0
DIA	0	0
DISHEVELED	0	0
Diversin	0	0
DJ1	1	0
DJ2	0	0
DKK	0	0
DKK2	0	0
DLC2	0	0
DLK	0	0
DLL1	0	0
DLL4	0	0
DNAJ	0	0
DNAPK	0	0
DOC2	0	0
DOCK180	0	0

DOK	0	0
Dopamine	0	0
DOPR	1	0
DP1	0	0
DP103	0	0
DR3	0	0
DR4	0	0
DR5	0	0
DRE	0	0
DREAM	0	0
Drp-1	0	0
DSH	0	0
DSP	0	0
dsRNA	0	0
DTX1	0	0
DVL2	0	0
DVL3	0	0
DYNAMIN	0	0
Dynein	0	0
DYNEIN	0	0
DYRK	1	0
E12	0	0
E1A	0	0
E2	1	0
E2F1	0	0
E2F4	0	0
E2F5	0	0
E3	1	0
E3B1	0	0
EBP	1	0
EBP1	0	0
EBS	0	0
ECM	0	0
ECSIT	0	0
EDG1	0	0
EDG2	0	0
EDN1	1	0
EDNRA	1	0
EEF1A2	0	0
EEF2	1	0
EEF2K	0	0

EF1A	0	0
EFA6	0	0
EGF	1	0
EGFR	1	1
EGR1	0	0
EGR2	0	0
EGR3	0	0
EIF1A	0	0
EIF2A	0	0
EIF2B	0	0
eIF2B	0	0
EIF2G	0	0
EIF3	0	0
EIF3S1	0	0
EIF3S2	0	0
EIF4	0	0
EIF4A	0	0
EIF4B	0	0
EIF4E	1	0
EIF-4EBP	0	0
EIF4F	0	0
EIF4G	0	0
EIF5	0	0
ELK1	0	0
ELK3	0	0
ELK4	0	0
ENDOPHILIN	0	0
ENG	0	0
ENKEPHALIN	0	0
EPAC1	0	0
EPHB2	1	0
EPHRIN	0	0
EPO	0	0
EPOR	1	0
EPPK1	0	0
EPS15	0	0
EPS8	0	0
ER	0	0
Era	1	0
ERb	1	0
ERBB2	1	0

ErbB3	0	0
ERBIN	0	0
ERE	0	0
ERK1	1	0
ERK2	1	0
ERK5	1	0
ERM	0	0
ERT	0	0
Estrogen	0	0
ETS1	0	0
ETS2	0	0
F10	1	0
F11	1	0
F13	0	0
F2	1	0
F3	1	0
F5	1	0
F7	1	0
F9	1	1
FADD	0	0
FAF	0	0
FAK	1	0
FAN	0	0
FANCA	0	0
FANCC	0	0
FANCD2	0	0
FANCE	0	0
FANCF	0	0
FANCG	0	0
FAP	0	0
FAP-1	0	0
FAS	0	0
FASCIN	0	0
FASLG	0	0
FBI1	0	0
FBW7	0	0
FBXO6	0	0
FCER1A	1	0
Fe	0	0
FER	0	0
FES	0	0

FGF	1	0
FGFR	1	1
FGR	0	0
FHL1	0	0
FHL2	0	0
Fibrinogen	1	0
FIBRONECTIN	0	0
FILAMIN	0	0
FIVEHT1AR	1	1
FIVEHT1CR	1	1
FIVEHT2AR	1	1
FIVEHT4R	1	0
FKBP	0	0
FKBP12	1	0
FKHRL1	0	0
FLASH	0	0
FLT3	1	1
FNTA	1	0
FODRIN	0	0
FORKHEAD	0	0
Forskolin	0	0
FOS	1	0
FOSB	0	0
FOUREBP1	0	0
FOXG1B	0	0
FOXH1	0	0
FRAT1	0	0
FRAT2	0	0
FRIZZLED	0	0
FRP	1	0
FRS2	0	0
FURIN	1	0
FXR	1	0
FYN	1	0
FZD2	0	0
FZD4	0	0
FZD5	0	0
FZD6	0	0
FZD7	0	0
FZD8	0	0
FZD9	0	0

FZR1	0	0
G1	0	0
G2	0	0
G3PD	1	0
GA	0	0
GAA	1	1
GAB	0	0
GAB1	0	0
GAB2	0	0
GABA	0	0
GABAAR	1	0
GABABR	1	0
GABARAP	0	0
GADD45	0	0
Gads	0	0
Gai	0	0
GAIP	0	0
GALPHAI	0	0
GALPHAO	0	0
GALPHAS	0	0
GALPHAZ	0	0
GAP	1	0
GAP43	0	0
GAQ	0	0
Gas	0	0
GAS	0	0
GAS1	0	0
GAS2	0	0
GAT1	1	0
Gat2	0	0
GATA1	0	0
GATA3	0	0
GATA4	0	0
Gb	1	0
GBETAGAMMA	0	0
Gbg	1	0
GC1	1	0
GC2	1	0
GCAP	1	0
GCK	1	0
GCN2	0	0

GCSF	0	0
GDNF	1	0
GDP	0	0
GEF	0	0
GELSOLIN	1	0
GEPHYRIN	1	0
GFAP	0	0
GFR	0	0
GH	0	0
GHR	1	0
Gia	0	0
GIPC	0	0
GIRK	1	0
GIT1	0	0
GJA1	1	0
GKAP	0	0
GLI1	0	0
GLI2	0	0
GLI3	0	0
glucocorticoid	0	0
GLUT4	0	0
GLUTAMATE	0	0
GLYCINE	0	0
Glycogen	1	0
GLYR	1	0
GLYT1	1	0
GMCSF	0	0
GNA12	0	0
GNA13	0	0
GNA16	0	0
GP130	0	0
GPCR	0	0
GR	1	0
Graf	0	0
GRAP	0	0
GRB10	0	0
GRB14	0	0
GRB2	1	0
Grb7	0	0
GRE	0	0
GREB1	0	0

GRIM19	1	0
GRIP	0	0
GRIP1	0	0
GRIT	0	0
GROUCHO	0	0
GRP1	0	0
GRP94	1	0
GSK3A	0	0
GSK3B	1	0
GTP	0	0
Gy	1	0
GZMB	1	0
H1	0	0
H2O2	0	0
H3	0	0
HAND1	0	0
HAND2	0	0
HAT1	0	0
HAX1	0	0
HBEGF	0	0
HBO1	0	0
HBX	0	0
HCK	1	0
HD	0	0
HDAC	1	0
HDAC1	1	0
HDAC2	1	0
HDAC3	1	0
HDAC5	1	0
HERP1	0	0
HES1	0	0
HES5	0	0
HES6	0	0
HEY1	0	0
HGF	1	0
HGS	1	0
HHIP	0	0
HIF1A	1	0
HINT1	1	0
HIP1	0	0
HIPK2	0	0

HIPPOCALCIN	0	0
HIST3H3	0	0
HIVEP3	0	0
HMG1	0	0
HMG2	0	0
HMGN1	0	0
HO	0	0
HOMER	0	0
HOP	0	0
HOXA9	0	0
HRI	0	0
Hrk	0	0
HSP27	0	0
Hsp40	0	0
Hsp60	0	0
hsp70	0	0
HSP72	1	0
Hsp90	1	0
HSPA8	1	0
HSPC	1	0
HSPG	1	0
HUR	0	0
HUS1	0	0
Hypoxia	0	0
I1	0	0
IAP	0	0
IBP	0	0
IBR	0	0
IC261	0	0
ICA512	0	0
ICAD	0	0
ICAT	0	0
ID1	0	0
ID2	0	0
ID3	0	0
ID4	0	0
Idax	0	0
I-FLICE	0	0
IFNA	0	0
IFNAR	1	0
IFNy	1	0

IFN-yR	1	0
IGF1	1	0
IGF1R	1	0
IHH	0	0
IKBA	0	0
IKBB	0	0
IKK2	1	0
IKKA	0	0
IKKe	0	0
IKKy	0	0
IL11	0	0
IL12	0	0
IL12RB2	0	0
IL-13	0	0
IL13RA1	0	0
IL13RA2	1	0
IL1A	1	0
IL1B	1	0
IL1R	1	0
IL2	1	0
IL2RG	1	0
IL3	1	0
IL4	0	0
IL4R	1	0
IL6	1	1
IL6R	1	0
IL8	1	0
IL9	0	0
ILK	0	0
ILPIP	0	0
I-mf	0	0
Importin	0	0
INHIBITOR2	1	0
INPP5A	0	0
Ins	1	0
INSR	1	1
Insulin	0	0
IP3	0	0
IP3R	0	0
IP6K2	0	0
IQGAP	0	0

IRAK	0	0
IRAK4	1	0
IRAK-M	0	0
IRF3	0	0
IRS1	1	0
IRS2	0	0
IRSP53	0	0
ISGF3G	0	0
ISRE	0	0
ITGA1	0	0
ITGA6	0	0
ITGB1	1	0
ITGB3	1	1
ITGB4	1	0
ITGB4BP	0	0
Itk	1	0
ITM2B	0	0
JAG1	0	0
JAG2	0	0
JAK1	1	0
JAK2	1	1
JAK3	1	0
JIP	1	0
JNK	1	0
JNK3	1	0
JNKK1	0	0
JUNB	0	0
JUND	0	0
K+	0	0
KAR	1	0
KB	0	0
KIF17B	0	0
KINESIN	0	0
KIR21	0	0
KIR23	0	0
KIR41	0	0
KIT	1	0
KOPR	1	0
KPNB1	0	0
krm	0	0
KRT17	0	0

KRT18	0	0
KRT7	1	0
KRT8	1	0
KSR	0	0
Ku70	0	0
KV11	1	0
KV12	1	0
KV14	0	0
KV41	0	0
KV42	1	0
L1	1	0
LAMA1	0	0
LAMB1	0	0
LAMC1	0	0
LAMR1	0	0
LARG	0	0
LAT	1	0
LCK	1	0
LEF	1	0
LEF1	0	0
LEP	0	0
LEPR	1	0
LFG	0	0
LFNG	0	0
Lgs	0	0
LIMK1	1	0
Livin	0	0
LMNA	0	0
LMNB1	0	0
LMNB2	0	0
LPL	1	1
LPS	0	0
LRP	0	0
LRP5	0	0
LRP6	0	0
LTYPECA	0	0
LXR	1	0
LYN	1	0
LZK	0	0
M1R	1	0
M2R	1	0

M3/6	0	0
M4R	1	0
MADD	0	0
MAGEA1	0	0
MAGI3	0	0
MAL	1	0
MALS	0	0
MAML1	0	0
MAML2	0	0
MAML3	0	0
MAP-1	0	0
MAP1A	1	0
MAP1B	0	0
MAP2	1	0
MAP3K2	1	0
MAP4K1	0	0
MAP4K3	0	0
MAP4K5	0	0
MAP4K6	0	0
MAPK11	1	0
MAPK13	1	0
MAPK9	1	0
MAPKAP2	1	0
MAPKAP-K3	1	0
MARCKS	0	0
MBD3	0	0
MBP	0	0
MCF2	0	0
MCL1	0	0
M-CSF	0	0
MD-2	1	0
mDIA	0	0
MDM2	1	0
MEF2	0	0
MEF2B	0	0
MEF2C	0	0
MEF2D	0	0
MEK1	1	0
MEK2	0	0
MEK3	0	0
MEK5	0	0

MEK6	0	0
MEKK	0	0
MEKK2	0	0
MEKK3	0	0
MEKK4	0	0
MERLIN	0	0
MET	1	0
METS	0	0
Mfn2	0	0
MFNG	0	0
MGLUR1	1	0
MGLUR7	1	0
MIG1	0	0
MIG6	0	0
MINT	0	0
MIP1A	0	0
MIP1B	0	0
MITF	0	0
MITR	1	0
Miz1	0	0
MKK7	0	0
MKP1	0	0
MKP2	0	0
MKP3	0	0
MKP4	0	0
MKP5	0	0
MLC	0	0
MLCK	0	0
MLCP	0	0
MLK	0	0
MLK1	1	0
MLK3	0	0
MMP7	1	0
MNK1	0	0
MNK2	0	0
MOPR	1	0
MOR	1	0
Mos	0	0
MP1	0	0
MPR	0	0
MRE11	0	0

MRLC	0	0
mRNA	0	0
MSK1	0	0
MSK2	0	0
MST1R	0	0
MST3	0	0
MTA2	0	0
MTOR	1	0
MUNC13	0	0
MUNC18	0	0
MUPP1	0	0
MURR1	0	0
MUSK	0	0
MYB	0	0
MYD88	0	0
MYF5	0	0
MYF6	0	0
MYOD	0	0
MYOG	0	0
myosin	0	0
MYOSIN	0	0
MYOSINV	0	0
MYT1	0	0
N41	0	0
NAIP	0	0
NAKED	0	0
NAP	0	0
NASCENTCHAIN	0	0
NBS1	0	0
NCADHERIN	0	0
NCK	0	0
NCK2	0	0
NCOR	0	0
NCS1	0	0
NDUFS1	1	0
NE	1	0
NEDD8	0	0
NEURABIN	0	0
NEUREXIN	0	0
NEUROFIBROMIN	0	0
NEUROLIGIN	0	0

NFAT	0	0
NFH	0	0
NFkB	1	0
NFM	0	0
NFYA	0	0
NFYB	0	0
NFYC	0	0
NGF	1	0
NGFR	0	0
NHERF	0	0
NICD	0	0
NIK	0	0
NLK	0	0
NMDAR	1	0
NME1	1	0
NMT	1	0
NO	0	0
NOGGIN	0	0
NOPR	1	0
NOS1	1	0
NOS3	1	0
NOTCH2	0	0
NOTCH3	0	0
NOTCH4	0	0
NOV	1	0
NOX1	0	0
Noxa	0	0
nPKC	1	0
NRCAM	0	0
Nrdp1	0	0
NRG	0	0
NSD1	0	0
NTF5	1	0
NTH1	0	0
NTRK1	1	0
NTYPECA	1	0
Nucling	0	0
NUMB	0	0
NUMBL	0	0
NUP153	0	0
NUP214	0	0

NUR77	0	0
N-WASP	0	0
O2	0	0
Omi	0	0
p107	0	0
p110Ia	0	0
p120	0	0
P130	0	0
P130Cas	0	0
P13K	1	0
P1433	0	0
p14ARF	0	0
P15	0	0
p16	0	0
p190RhoGAP	0	0
P2	1	0
P21	0	0
p25	0	0
p27	0	0
p300	0	0
p32	0	0
p34	0	0
P35	0	0
P35611	0	0
p38	1	0
p38gamma	1	0
P50	0	0
p53	1	0
p55gamma	1	0
p6	1	0
p65	0	0
p67phox	0	0
p68	0	0
p70s6K	0	0
P73	0	0
p85	0	0
p85beta	1	0
p90RSK	0	0
PA	0	0
PABP	0	0
PAC1	0	0

PACAP	0	0
PAF	0	0
PAFR	0	0
PAG	0	0
PAK1	0	0
PAK2	0	0
PAK6	0	0
PAR1	1	0
PAR2	0	0
PAR4	0	0
PARD3	0	0
PARP	1	0
PATZ	0	0
PAX2	0	0
PAX5	0	0
PAX8	0	0
PBP	1	0
PCAF	1	0
PCLy	0	0
PCNA	0	0
PDE	0	0
PDE1A	1	0
PDE1B	1	0
PDE1C	1	0
PDE2	1	0
PDE3A	1	0
PDE3B	1	0
PDE4A	1	0
PDE4B	1	0
PDE4C	1	0
PDE4D	1	0
PDE5A	1	1
PDE6A	1	0
PDE6B	1	0
PDE6G	1	0
PDGF	0	0
PDGFB	0	0
PDGFR	1	0
PDGFRA	1	0
PDI	1	0
PDK1	1	0

PDK2	1	0
PDPK1	1	0
PDZGEF	0	0
PDZK1	0	0
PELP1	0	0
PEN2	0	0
PER	0	0
PERK	0	0
PFN1	1	0
pGC	0	0
PGC1A	0	0
PHKA2	0	0
PI3K	1	0
PI3Ky	1	0
PI3P	0	0
PI-4-P5K	0	0
PIAS	0	0
PIAS3	0	0
PIAS4	0	0
PIB5PA	0	0
PICCOLO	0	0
PICK1	0	0
PIK3C2B	0	0
PIK3CD	1	0
PIM2	0	0
PIN1	1	0
PIP2	0	0
PIP3	0	0
PIP5K	0	0
PIR	0	0
PITPN	1	0
PKA	1	0
PKAc	0	0
PKC	0	0
PKCA	1	0
PKCE	0	0
PKCz	0	0
PKD	0	0
PKG	0	0
PKI	1	0
PKLR	1	0

PKN	0	0
PKN2	0	0
PKR	0	0
PLA2	1	0
PLA2P	0	0
PLAGL1	0	0
PLASMIN	0	0
PLC	1	0
PLCb	0	0
PLCD1	1	0
PLCE	1	0
PLCE1	0	0
PLCg-2	0	0
PLCy	0	0
PLD	1	0
PLD1	1	0
plectin	0	0
PLG	1	0
PLK1	1	0
PLK3	0	0
PLSCR1	0	0
PMCA	0	0
POFUT1	0	0
POLR1B	0	0
POLR2A	0	0
POR1	0	0
POTASSIUM	0	0
PP1	0	0
PP2A	0	0
PP2B	0	0
PP2C	0	0
PP32	0	0
PP5	0	0
PPARA	1	1
PPARB	0	0
PPARG	1	1
PPP1CC	1	0
PPP2CA	1	0
PPP2R2A	1	0
PPP2R5B	0	0
PPP2R5C	1	0

PPP3R2	1	0
PQCaCh	0	0
PR-A	0	0
PRAK	0	0
PRF1	0	0
PRK2	0	0
PRKAR1A	1	0
PRKAR1B	0	0
PRKCD	1	0
PRKCG	1	0
PRKCI	1	0
PRMT1	1	0
PROFILIN	0	0
PROKR1	0	0
PrP	1	0
PSD93	0	0
PSD95	0	0
PSEN1	0	0
PSEN2	0	0
PSMC4	0	0
PSMD4	0	0
PTC	0	0
PTCH	0	0
PTCH2	0	0
PTEN	0	0
PTK	1	0
PTK6	1	0
PTMA	0	0
PTP	0	0
PTP1B	1	0
PTP1D	0	0
PTPA	1	0
PTPB1	1	0
PTPN12	0	0
PTPRO	0	0
PTP-SL	0	0
Puma	0	0
PXN	0	0
PYGB	1	0
Pygo	0	0
RAB3	0	0

RAB5	0	0
RABGDI	0	0
RABPHILIN	0	0
RAC1	1	0
RAC3	0	0
RACK	0	0
RAD1	0	0
RAD17	0	0
RAD50	0	0
RAD51	1	0
Rad54l2	0	0
RAD9	0	0
RADIXIN	0	0
RAF1	1	0
RAIDD	0	0
RAL	0	0
RALB	0	0
RALBP1	0	0
RALGDS	0	0
RAN	1	0
RANBP1	0	0
RANBP2	0	0
RANBP9	0	0
RanGAP	0	0
RAP1	0	0
RAP1A	0	0
RAP1B	0	0
RAP1GAP	0	0
RAP2	1	0
RAP30	0	0
RAP74	0	0
RAR	1	0
RAS	0	0
RASGAP	0	0
RASGRF	0	0
RasGRP	0	0
RASGRP1	0	0
RASGRP3	0	0
RB	1	0
RBBP7	0	0
REA	0	0

RELA	1	0
RELN	0	0
REPS1	0	0
REPS2	0	0
REQ	0	0
RET	1	0
RGS	0	0
RGS16	0	0
RGS2	0	0
RGS4	0	0
RHEB	1	0
Rho	1	1
RHOA	1	0
RhoGDP	0	0
RHOGEFs	0	0
RhoGTP	0	0
RIC	0	0
RICS	0	0
RIIa	0	0
RIIb	0	0
RIM	0	0
RIN	0	0
RIN1	0	0
RING1	0	0
RIP	0	0
RIP140	1	0
RKIP	0	0
RLC	0	0
RNF4	0	0
RNPK	0	0
RNTRE	0	0
ROC1	0	0
ROCK1	1	0
ROCK2	1	0
ROR2	0	0
ROS	0	0
RPS6	0	0
RPTK	1	0
RSK	1	0
RSK2	0	0
RTKN	0	0

RTYPECA	0	0
RUVBL1	0	0
RXR	1	0
Ryk	0	0
RYR	1	1
S1P	0	0
S6	0	0
S60	0	0
SAC	0	0
SAG	0	0
SALL1	0	0
SAM68	0	0
SAP102	0	0
SAP18	0	0
SAP30	0	0
SAP97	0	0
SAPAP	0	0
Sara	0	0
SCF	0	0
SDF1	1	0
SEC61P	0	0
SEC63P	0	0
SEK	0	0
SENP1	0	0
SEROTONIN	0	0
SERPINC1	1	0
Ser-prot	0	0
SET	0	0
SF1	1	0
SFN	0	0
SH2D3C	0	0
SH3BGRL	0	0
SH3GL3	0	0
SH3KBP1	0	0
SHANK	0	0
SHARP	0	0
SHC	1	0
SHH	0	0
SHIP	0	0
SHIP2	0	0
SHOC2	0	0

SHP	1	0
SHP1	0	0
SHP2	1	0
SIAH	0	0
Sin3	0	0
SIVA1	0	0
SKI	0	0
SKIP	0	0
SKP1	1	0
SKP2	0	0
SLC25A4	1	0
SLC9A1	1	0
SLP-76	0	0
SMAD1	0	0
SMAD2	1	0
SMAD3	0	0
SMAD4	0	0
SMAD5	0	0
SMAD6	0	0
SMAD7	0	0
SMARCA4	0	0
SMO	1	0
SMPD1	1	0
SMPD2	0	0
SMRT	0	0
SMURF1	0	0
SMURF2	0	0
SNAI1	0	0
SNAP25	1	0
SNAPIN	0	0
SNCA	0	0
SNIP1	0	0
SnoN	0	0
SNRPD2	0	0
SNW1	0	0
SNX1	0	0
SNX2	0	0
SNX4	0	0
SNX6	0	0
SOCS	0	0
SOCS1	0	0

SOCS3	0	0
SOCS5	0	0
SOCS6	0	0
SOD	1	0
SODD	0	0
SODIUM	0	0
SORCIN	0	0
SOS1	0	0
SOS2	0	0
SOX1	0	0
SP1	0	0
SP3	0	0
SPAL	0	0
SPDEF	0	0
SPECTRIN	0	0
SPHK1	0	0
SPHK2	0	0
SPINOPHILIN	0	0
SPRED1	0	0
SPRED2	0	0
SPRY	0	0
SPTAN1	0	0
SRA1	0	0
SRC	1	0
SRE	0	0
SRF	0	0
SRP54	0	0
SRPR	0	0
SRY	0	0
SS	0	0
SSCAM	0	0
SSTR1	1	0
SSTR2	1	0
STAMBPL1	0	0
STAP1	0	0
STARGAZIN	0	0
STAT	0	0
STAT1	0	0
STAT2	0	0
STAT3	0	0
STAT4	0	0

STAT5	0	0
STAT5B	1	0
STAT6	0	0
STEP	0	0
STK11IP	0	0
STK36	0	0
STRAP	0	0
STUB1	0	0
STXBP1	0	0
SUFU	0	0
SUMO1	0	0
SUMO2	0	0
SV2A	1	0
SVIL	0	0
SYK	1	0
SYNAPSIN	0	0
SYNAPTOBREVIN	1	0
SYNAPTOPHYSIN	0	0
SYNAPTOTAGMIN	0	0
SYNDECAN	0	0
SYNGAP	0	0
SYNTAPHILIN	0	0
SYNTAXIN	0	0
SYNTENIN	0	0
SYNTROPHIN	0	0
T3R	1	0
Ta	1	0
TAB1	0	0
TAB2	0	0
TAK1	0	0
TALIN	0	0
TAMALIN	0	0
TANK	0	0
TAU	0	0
TAX	0	0
Tb	0	0
TBK1	0	0
TBP	0	0
TBPIP	0	0
TBR1	0	0
TBX2	0	0

TCF	1	0
TCF12	0	0
TCF3	0	0
TCF4	0	0
TCF7	0	0
TCR	0	0
TEC	0	0
TEP1	0	0
TERT	1	0
TFDP2	0	0
TFF1	0	0
TFF2	1	0
TFIIB	0	0
TFIID	0	0
TFIIH	0	0
TGFB	1	0
TGFB2	0	0
TGFB3	1	0
TGFBR1	1	0
TGFBR2	1	0
TGFBR3	0	0
TGFBRAP1	0	0
TGIF	0	0
TGM2	1	0
THC	1	0
THRAP2	0	0
TIAM1	0	0
TID1	0	0
TIEG2	0	0
TIF2	1	0
TIP1	0	0
TIRAP	0	0
TLR1	0	0
TLR10	0	0
TLR2	1	0
TLR3	0	0
TLR4	1	0
TLR5	0	0
TLR6	0	0
TLR7	1	1
TLR8	1	0

TLR9	1	0
TNF	1	1
TNFB	1	0
TNFR1	1	0
TNFR2	1	0
TNIP1	0	0
TNKS	0	0
TOB	0	0
TOLLIP	0	0
TopoII	1	0
TOR	0	0
TP53BP1	0	0
Tpl-2	0	0
TR4	0	0
TRADD	0	0
TRAF1	0	0
TRAF2	0	0
TRAF3	0	0
TRAF6	0	0
TRE	0	0
TREH	0	0
TRIO	0	0
TRKB	1	0
tRNA	0	0
TRX	0	0
TRYPSIN	1	0
TSA	1	0
TSAP6	0	0
TSC1	0	0
TSC2	0	0
Tsh	0	0
TSP1	0	0
TUBB4Q	1	0
TUBBY	0	0
TUBULIN	1	0
TWIST	0	0
Ty	0	0
TYK2	1	0
Ub	0	0
UBC9	0	0
UBE1L	0	0

UBE2D1	0	0
UBE2D2	1	0
UBE2D3	0	0
UBE2M	0	0
UBE3A	0	0
UbiL	0	0
UXT	0	0
VAMP	0	0
VANGL2	0	0
VASP	0	0
VAV	0	0
VAV2	0	0
VAV3	0	0
VCL	0	0
VDAC2	1	0
VDR	1	1
VEGF	1	0
VEGFR	0	0
VELI1	0	0
VIL2	0	0
VILIP	0	0
VIM	0	0
VIP	0	0
VITRONECTIN	1	0
VLDLR	0	0
VPAC2R	0	0
VRK1	0	0
WAVE1	0	0
WAVE2	0	0
WAVE3	0	0
WDR12	0	0
WIF1	0	0
WNT	0	0
WNT2	0	0
WNT3	0	0
WNT4	0	0
WNT5A	0	0
WNT6	0	0
WNT7A	0	0
XAF1	0	0
XAP2	0	0

XDH	1	0
XIAP	1	0
YAP1	0	0
YES	1	0
YOTIAO	0	0
YY1	0	0
ZAK	0	0
ZAP70	1	0
ZEB1	0	0
ZFHX1B	0	0
ZFYVE9	0	0
ZNF259	0	0
Zyxin	0	0

Table S4. Drug-targets and side-effects in HSN network. It includes information of genes, drug-targets, and side-effects in HSN network.

GeneName	Drug-target (1:True,0:False)	Side-effect (1:True,0:False)
37165	0	0
37530	0	0
14-3-3	0	0
4-1BB	0	0
4-1BBL	0	0
7TMR	0	0
A1R	1	0
A20	0	0
a2-Antiplasmin	0	0
A2AR	1	0
AA	0	0
ABI2	0	0
ABL1	1	1
ABP1	1	0
Ab-R	0	0
AC	0	0
AC1	1	0
AC2	1	0
AC5	1	0
ACC	1	0
ACH	1	0
AChE	1	1
ACHRE	1	0
Acinus	0	0
ACK	1	0
ACT	0	0
ACTG	0	0
ACTIN	0	0
ACTN1	0	0
ACTR	0	0
ACVRL1	1	0
ADAM10	1	0
ADAM12	0	0
ADAM17	1	0
ADAPTIN	0	0

ADDUCIN	0	0
ADENOSINE	0	0
ADPRIBSYCYCLASE	0	0
AES	0	0
AFX	0	0
AGT	1	0
AGTR1	1	1
AGTR2	1	0
AHR	1	0
AIF	1	0
AIP4	0	0
AKAP	0	0
AKAP13	0	0
AKAP15	0	0
Akkt	1	0
AKT	1	0
ALDOA	1	0
Alg-2	0	0
ALK3	0	0
ALPHA1AR	1	0
ALPHA2AR	1	0
ALPHA7NACHR	1	0
ALPHAACTININ	0	0
ALPHACATENIN	0	0
AMISYN	0	0
AML1	0	0
AML3	0	0
AMP	1	0
AMPAR	1	0
AMPHIPHYSIN	0	0
AMPK	1	0
ANANDAMIDE	0	0
ANAPC1	0	0
ANAPC2	0	0
ANF	0	0
ANKYRIN	0	0
AP2	0	0
AP2A1	0	0
AP2B1	0	0
APAF1	1	0
APC	1	0

APE1	1	0
APIP	0	0
APLP1	0	0
APO2L	0	0
APO3L	0	0
APOER2	0	0
APOLLON	0	0
APP	1	0
APPL	0	0
APPL2	0	0
APS	0	0
AR	1	0
ARA160	0	0
ARA54	0	0
ARA55	0	0
ARA70	0	0
ARAD	0	0
A-Raf	1	0
ARAI	0	0
ARF	0	0
ARF1	1	0
ARF4	1	0
ARFGAP	0	0
ARFGEF	0	0
ARHGAP1	0	0
ARHGAP4	0	0
ARHGAP5	0	0
ARHGAP6	0	0
ARHGEF1	0	0
ARHGEF11	0	0
ARHGEF5	0	0
ARHGEF6	0	0
ARIP2	0	0
ARNIP	0	0
ARNO	1	0
ARNT	0	0
ARP2	1	0
ARP23	1	0
ARP3	1	0
ARRB2	0	0
ARTS	0	0

ASAP1	0	0
ASC	0	0
ASCL1	0	0
ASK1	0	0
ATF1	1	0
ATF2	1	0
ATF3	1	0
ATM	1	0
ATP	0	0
ATR	0	0
ATRIP	0	0
Aven	0	0
AVP	0	0
AXAM2	0	0
AXIN	1	0
AXIN2	0	0
B2AR	1	0
BAD	1	0
BAG1	1	0
BAK	1	0
BAP31	0	0
bARK	1	0
BAX	0	0
BCL10	0	0
BCL2	1	0
BCL2L1	1	0
Bcl-b	0	0
BCLG	0	0
Bcl-rambo	0	0
Bcl-w	0	0
BCR	1	1
BDNF	1	0
BETAARRESTIN	0	0
BFAR	0	0
BI-1	0	0
BID	0	0
BIK	0	0
BIM	0	0
BIP	1	0
BIRC5	1	0
BLNK	0	0

BMAL1	0	0
Bmf	0	0
BMP-10	0	0
BMP-2	0	0
BMP-4	0	0
BMP-5	0	0
BMP-7	0	0
BMPR1	0	0
BMPR2	0	0
BNIP3	0	0
BNIP3L	0	0
Bok	0	0
BP75	0	0
BPAG1	0	0
BPAG2	0	0
BR1R	0	0
BRADYKININ	0	0
bRAF	1	0
BRCA1	0	0
BRCA2	0	0
BTK	1	0
BTRC	0	0
C3G	0	0
Ca++	0	0
CABIN1	0	0
CAD	1	0
cADPR	0	0
CALBRAIN	0	0
CALCINEURIN	0	0
CALM3	0	0
CALMODULIN	1	0
CALNEXIN	1	0
Calpain1	1	0
Calpain2	0	0
Calpastatin	1	0
CALRETICULIN	1	0
CAM	0	0
CAMK	1	0
CAMK1	0	0
CAMK2	1	0
CAMK2A	1	0

CAMKIV	1	0
CAMKK	0	0
CAMKPASE	0	0
CAMP	1	0
cAMPGEFII	0	0
CAP	0	0
CAPRI	0	0
cAR	1	0
CARD14	0	0
CARM1	0	0
CASK	1	0
CASKIN	0	0
CASP1	1	0
CASP10	0	0
CASP2	0	0
CASP3	1	0
CASP6	0	0
CASP7	1	0
CASP8	0	0
CASP9	0	0
Catalase	1	0
Cathepsin	1	0
CAV2	0	0
CAVEOLIN	0	0
CB1R	1	0
CB2R	1	0
CBL	0	0
CBLB	0	0
CBP	1	0
Cby	0	0
CCL11	1	0
CCL5	1	0
CCNA2	1	0
CCNB1	0	0
CCNB2	0	0
CCND1	1	0
CCND2	0	0
CCND3	0	0
CCNE1	0	0
CCNH	0	0
CCR3	0	0

CCR5	1	0
CCT4	0	0
Cd++	0	0
CD14	0	0
CD151	0	0
CD19	1	0
CD28	0	0
CD3	1	0
CD36	0	0
CD4	1	0
CD40	0	0
CD40L	0	0
CD44	1	0
CD45	0	0
CD47	0	0
CDC16	0	0
CDC25	0	0
CDC25A	0	0
CDC25C	0	0
CDC27	0	0
CDC34	0	0
CDC37	0	0
CDC42	1	0
CDH1	0	0
CDK1	1	0
CDK2	1	0
CDK4	1	0
CDK5	1	0
CDK6	1	0
CDK7	1	0
CDK9	1	0
CDX1	0	0
CEACAM1	1	0
CEBPA	0	0
CEBPB	0	0
CEK1	0	0
CEM15	0	0
Cer	0	0
CERAMIDE	0	0
CGMP	0	0
CHAPSYN110	0	0

CHK	0	0
CHK1	1	0
CHK2	1	0
CHLORIDE	0	0
CHN1	0	0
CHOLESTEROL	0	0
CHOP10	0	0
CHORDIN	0	0
CHREBP	0	0
cIAP	0	0
cIAP1	0	0
cIAP2	0	0
CIPP	0	0
CIR	1	0
CITED1	0	0
CITRON	0	0
c-JUN	1	0
CK19	0	0
CK1a	0	0
CK1d	1	0
CK1e	1	0
CK2	1	0
CK2A2	1	0
CKI	1	0
CKS1A	0	0
CL	0	0
CLATHRIN	0	0
CLCA1	0	0
CLCA2	0	0
CLIP170	0	0
Clk	1	0
CLOCK	0	0
CLTC	0	0
cMAF	0	0
c-Myc	0	0
Cn	0	0
CNG	0	0
CNTN1	0	0
COFILIN1	1	0
COFILIN2	0	0
COLLAGEN	1	0

COMPLEXIN	0	0
COPS5	0	0
CORTACTIN	0	0
CPI	0	0
CPI17	0	0
cPLA2	0	0
CRAC	0	0
CRE	0	0
CREB	1	0
CREM	0	0
CRI2	0	0
CRIPT	0	0
CRK	0	0
CRKL	0	0
CRM1	0	0
CRMP2	0	0
CRY	1	0
CryAB	0	0
CSF1R	1	0
CSF2RB	1	0
CSK	1	0
CSL	0	0
CSNK1D	0	0
CSNK1E	0	0
CSP	0	0
CSX	0	0
CTBP1	1	0
CTBP2	0	0
CTCF	0	0
CTIP	0	0
CTLA-4	1	0
CTNNB1	1	0
CTNND1	0	0
CTSD	1	0
CUL1	0	0
CXCR4	1	0
CYCS	1	0
CYP19A1	1	1
CypA	1	0
CYTOCHROMEC	1	0
D1	0	0

D1R	1	0
D2	0	0
D2R	1	0
D3R	1	1
D4-GDI	0	0
DAAM1	0	0
DAB1	0	0
DAB2	0	0
DAG	0	0
DAP	0	0
DAP10	0	0
DAPK	1	0
DARPP-32	0	0
dATP	0	0
DAX-1	1	0
DAXX	0	0
DCP1	1	1
DCP1A	0	0
DcR3	0	0
DD	1	0
DEDD1	0	0
DEFCAP	0	0
DELTA	1	0
DENSIN	0	0
DFFB	0	0
DG	0	0
DGK	0	0
DHH	0	0
DHPG	0	0
DIA	0	0
DISHEVELED	0	0
Diversin	0	0
DJ1	1	0
DJ2	0	0
DKK	0	0
DKK2	0	0
DLC2	0	0
DLK	0	0
DLL1	0	0
DLL4	0	0
DNAJ	0	0

DNAPK	0	0
DOC2	0	0
DOCK180	0	0
DOK	0	0
Dopamine	0	0
DOPR	1	0
DP1	0	0
DP103	0	0
DR3	0	0
DR4	0	0
DR5	0	0
DRE	0	0
DREAM	0	0
Drp-1	0	0
DSH	0	0
DSP	0	0
dsRNA	0	0
DTX1	0	0
DVL2	0	0
DVL3	0	0
DYNAMIN	0	0
Dynein	0	0
DYNEIN	0	0
DYRK	1	0
E12	0	0
E1A	0	0
E2	1	0
E2F1	0	0
E2F4	0	0
E2F5	0	0
E3	1	0
E3B1	0	0
EBP	1	0
EBP1	0	0
EBS	0	0
ECM	0	0
ECSIT	0	0
EDG1	0	0
EDG2	0	0
EDN1	1	0
EDNRA	1	0

EEF1A2	0	0
EEF2	1	0
EEF2K	0	0
EF1A	0	0
EFA6	0	0
EGF	1	0
EGFR	1	1
EGR1	0	0
EGR2	0	0
EGR3	0	0
EIF1A	0	0
EIF2A	0	0
EIF2B	0	0
eIF2B	0	0
EIF2G	0	0
EIF3	0	0
EIF3S1	0	0
EIF3S2	0	0
EIF4	0	0
EIF4A	0	0
EIF4B	0	0
EIF4E	1	0
EIF-4EBP	0	0
EIF4F	0	0
EIF4G	0	0
EIF5	0	0
ELK1	0	0
ELK3	0	0
ELK4	0	0
ENDOPHILIN	0	0
ENG	0	0
ENKEPHALIN	0	0
EPAC1	0	0
EPHB2	1	0
EPHRIN	0	0
EPO	0	0
EPOR	1	0
EPPK1	0	0
EPS15	0	0
EPS8	0	0
ER	0	0

Era	1	0
ERb	1	0
ERBB2	1	0
ErbB3	0	0
ERBIN	0	0
ERE	0	0
ERK1	1	0
ERK2	1	0
ERK5	1	0
ERM	0	0
ERT	0	0
Estrogen	0	0
ETS1	0	0
ETS2	0	0
F10	1	0
F11	1	0
F13	0	0
F2	1	0
F3	1	0
F5	1	0
F7	1	0
F9	1	1
FADD	0	0
FAF	0	0
FAK	1	0
FAN	0	0
FANCA	0	0
FANCC	0	0
FANCD2	0	0
FANCE	0	0
FANCF	0	0
FANCG	0	0
FAP	0	0
FAP-1	0	0
FAS	0	0
FASCIN	0	0
FASLG	0	0
FBI1	0	0
FBW7	0	0
FBXO6	0	0
FCER1A	1	0

Fe	0	0
FER	0	0
FES	0	0
FGF	1	0
FGFR	1	1
FGR	0	0
FHL1	0	0
FHL2	0	0
Fibrinogen	1	0
FIBRONECTIN	0	0
FILAMIN	0	0
FIVEHT1AR	1	1
FIVEHT1CR	1	1
FIVEHT2AR	1	1
FIVEHT4R	1	0
FKBP	0	0
FKBP12	1	0
FKHRL1	0	0
FLASH	0	0
FLT3	1	1
FNTA	1	0
FODRIN	0	0
FORKHEAD	0	0
Forskolin	0	0
FOS	1	0
FOSB	0	0
FOUREBP1	0	0
FOXG1B	0	0
FOXH1	0	0
FRAT1	0	0
FRAT2	0	0
FRIZZLED	0	0
FRP	1	0
FRS2	0	0
FURIN	1	0
FXR	1	0
FYN	1	0
FZD2	0	0
FZD4	0	0
FZD5	0	0
FZD6	0	0

FZD7	0	0
FZD8	0	0
FZD9	0	0
FZR1	0	0
G1	0	0
G2	0	0
G3PD	1	0
GA	0	0
GAA	1	1
GAB	0	0
GAB1	0	0
GAB2	0	0
GABA	0	0
GABAAR	1	0
GABABR	1	0
GABARAP	0	0
GADD45	0	0
Gads	0	0
Gai	0	0
GAIP	0	0
GALPHAI	0	0
GALPHAO	0	0
GALPHAS	0	0
GALPHAZ	0	0
GAP	1	0
GAP43	0	0
GAQ	0	0
Gas	0	0
GAS	0	0
GAS1	0	0
GAS2	0	0
GAT1	1	0
Gat2	0	0
GATA1	0	0
GATA3	0	0
GATA4	0	0
Gb	1	0
GBETAGAMMA	0	0
Gbg	1	0
GC1	1	0
GC2	1	0

GCAP	1	0
GCK	1	0
GCN2	0	0
GCSF	0	0
GDNF	1	0
GDP	0	0
GEF	0	0
GELSOLIN	1	0
GEPHYRIN	1	0
GFAP	0	0
GFR	0	0
GH	0	0
GHR	1	0
Gia	0	0
GIPC	0	0
GIRK	1	0
GIT1	0	0
GJA1	1	0
GKAP	0	0
GLI1	0	0
GLI2	0	0
GLI3	0	0
glucocorticoid	0	0
GLUT4	0	0
GLUTAMATE	0	0
GLYCINE	0	0
Glycogen	1	0
GLYR	1	0
GLYT1	1	0
GMCSF	0	0
GNA12	0	0
GNA13	0	0
GNA16	0	0
GP130	0	0
GPCR	0	0
GR	1	0
Graf	0	0
GRAP	0	0
GRB10	0	0
GRB14	0	0
GRB2	1	0

Grb7	0	0
GRE	0	0
GREB1	0	0
GRIM19	1	0
GRIP	0	0
GRIP1	0	0
GRIT	0	0
GROUCHO	0	0
GRP1	0	0
GRP94	1	0
GSK3A	0	0
GSK3B	1	0
GTP	0	0
Gy	1	0
GZMB	1	0
H1	0	0
H2O2	0	0
H3	0	0
HAND1	0	0
HAND2	0	0
HAT1	0	0
HAX1	0	0
HBEGF	0	0
HBO1	0	0
HBX	0	0
HCK	1	0
HD	0	0
HDAC	1	0
HDAC1	1	0
HDAC2	1	0
HDAC3	1	0
HDAC5	1	0
HERP1	0	0
HES1	0	0
HES5	0	0
HES6	0	0
HEY1	0	0
HGF	1	0
HGS	1	0
HHIP	0	0
HIF1A	1	0

HINT1	1	0
HIP1	0	0
HIPK2	0	0
HIPPOCALCIN	0	0
HIST3H3	0	0
HIVEP3	0	0
HMG1	0	0
HMG2	0	0
HMGN1	0	0
HO	0	0
HOMER	0	0
HOP	0	0
HOXA9	0	0
HRI	0	0
Hrk	0	0
HSP27	0	0
Hsp40	0	0
Hsp60	0	0
hsp70	0	0
HSP72	1	0
Hsp90	1	0
HSPA8	1	0
HSPC	1	0
HSPG	1	0
HUR	0	0
HUS1	0	0
Hypoxia	0	0
I1	0	0
IAP	0	0
IBP	0	0
IBR	0	0
IC261	0	0
ICA512	0	0
ICAD	0	0
ICAT	0	0
ID1	0	0
ID2	0	0
ID3	0	0
ID4	0	0
Idax	0	0
I-FLICE	0	0

IFNA	0	0
IFNAR	1	0
IFNy	1	0
IFN-yR	1	0
IGF1	1	0
IGF1R	1	0
IHH	0	0
IKBA	0	0
IKBB	0	0
IKK2	1	0
IKKA	0	0
IKKe	0	0
IKKy	0	0
IL11	0	0
IL12	0	0
IL12RB2	0	0
IL-13	0	0
IL13RA1	0	0
IL13RA2	1	0
IL1A	1	0
IL1B	1	0
IL1R	1	0
IL2	1	0
IL2RG	1	0
IL3	1	0
IL4	0	0
IL4R	1	0
IL6	1	1
IL6R	1	0
IL8	1	0
IL9	0	0
ILK	0	0
ILPIP	0	0
I-mf	0	0
Importin	0	0
INHIBITOR2	1	0
INPP5A	0	0
Ins	1	0
INSR	1	1
Insulin	0	0
IP3	0	0

IP3R	0	0
IP6K2	0	0
IQGAP	0	0
IRAK	0	0
IRAK4	1	0
IRAK-M	0	0
IRF3	0	0
IRS1	1	0
IRS2	0	0
IRSP53	0	0
ISGF3G	0	0
ISRE	0	0
ITGA1	0	0
ITGA6	0	0
ITGB1	1	0
ITGB3	1	1
ITGB4	1	0
ITGB4BP	0	0
Itk	1	0
ITM2B	0	0
JAG1	0	0
JAG2	0	0
JAK1	1	0
JAK2	1	1
JAK3	1	0
JIP	1	0
JNK	1	0
JNK3	1	0
JNKK1	0	0
JUNB	0	0
JUND	0	0
K+	0	0
KAR	1	0
KB	0	0
KIF17B	0	0
KINESIN	0	0
KIR21	0	0
KIR23	0	0
KIR41	0	0
KIT	1	0
KOPR	1	0

KPNB1	0	0
krm	0	0
KRT17	0	0
KRT18	0	0
KRT7	1	0
KRT8	1	0
KSR	0	0
Ku70	0	0
KV11	1	0
KV12	1	0
KV14	0	0
KV41	0	0
KV42	1	0
L1	1	0
LAMA1	0	0
LAMB1	0	0
LAMC1	0	0
LAMR1	0	0
LARG	0	0
LAT	1	0
LCK	1	0
LEF	1	0
LEF1	0	0
LEP	0	0
LEPR	1	0
LFG	0	0
LFNG	0	0
Lgs	0	0
LIMK1	1	0
Livin	0	0
LMNA	0	0
LMNB1	0	0
LMNB2	0	0
LPL	1	1
LPS	0	0
LRP	0	0
LRP5	0	0
LRP6	0	0
LTYPECA	0	0
LXR	1	0
LYN	1	0

LZK	0	0
M1R	1	0
M2R	1	0
M3/6	0	0
M4R	1	0
MADD	0	0
MAGEA1	0	0
MAGI3	0	0
MAL	1	0
MALS	0	0
MAML1	0	0
MAML2	0	0
MAML3	0	0
MAP-1	0	0
MAP1A	1	0
MAP1B	0	0
MAP2	1	0
MAP3K2	1	0
MAP4K1	0	0
MAP4K3	0	0
MAP4K5	0	0
MAP4K6	0	0
MAPK11	1	0
MAPK13	1	0
MAPK9	1	0
MAPKAP2	1	0
MAPKAP-K3	1	0
MARCKS	0	0
MBD3	0	0
MBP	0	0
MCF2	0	0
MCL1	0	0
M-CSF	0	0
MD-2	1	0
mDIA	0	0
MDM2	1	0
MEF2	0	0
MEF2B	0	0
MEF2C	0	0
MEF2D	0	0
MEK1	1	0

MEK2	0	0
MEK3	0	0
MEK5	0	0
MEK6	0	0
MEKK	0	0
MEKK2	0	0
MEKK3	0	0
MEKK4	0	0
MERLIN	0	0
MET	1	0
METS	0	0
Mfn2	0	0
MFNG	0	0
MGLUR1	1	0
MGLUR7	1	0
MIG1	0	0
MIG6	0	0
MINT	0	0
MIP1A	0	0
MIP1B	0	0
MITF	0	0
MITR	1	0
Miz1	0	0
MKK7	0	0
MKP1	0	0
MKP2	0	0
MKP3	0	0
MKP4	0	0
MKP5	0	0
MLC	0	0
MLCK	0	0
MLCP	0	0
MLK	0	0
MLK1	1	0
MLK3	0	0
MMP7	1	0
MNK1	0	0
MNK2	0	0
MOPR	1	0
MOR	1	0
Mos	0	0

MP1	0	0
MPR	0	0
MRE11	0	0
MRLC	0	0
mRNA	0	0
MSK1	0	0
MSK2	0	0
MST1R	0	0
MST3	0	0
MTA2	0	0
MTOR	1	0
MUNC13	0	0
MUNC18	0	0
MUPP1	0	0
MURR1	0	0
MUSK	0	0
MYB	0	0
MYD88	0	0
MYF5	0	0
MYF6	0	0
MYOD	0	0
MYOG	0	0
myosin	0	0
MYOSIN	0	0
MYOSINV	0	0
MYT1	0	0
N41	0	0
NAIP	0	0
NAKED	0	0
NAP	0	0
NASCENTCHAIN	0	0
NBS1	0	0
NCADHERIN	0	0
NCK	0	0
NCK2	0	0
NCOR	0	0
NCS1	0	0
NDUFS1	1	0
NE	1	0
NEDD8	0	0
NEURABIN	0	0

NEUREXIN	0	0
NEUROFIBROMIN	0	0
NEUROLIGIN	0	0
NFAT	0	0
NFH	0	0
NFkB	1	0
NFM	0	0
NFYA	0	0
NFYB	0	0
NFYC	0	0
NGF	1	0
NGFR	0	0
NHERF	0	0
NICD	0	0
NIK	0	0
NLK	0	0
NMDAR	1	0
NME1	1	0
NMT	1	0
NO	0	0
NOGGIN	0	0
NOPR	1	0
NOS1	1	0
NOS3	1	0
NOTCH2	0	0
NOTCH3	0	0
NOTCH4	0	0
NOV	1	0
NOX1	0	0
Noxa	0	0
nPKC	1	0
NRCAM	0	0
Nrdp1	0	0
NRG	0	0
NSD1	0	0
NTF5	1	0
NTH1	0	0
NTRK1	1	0
NTYPECA	1	0
Nucling	0	0
NUMB	0	0

NUMBL	0	0
NUP153	0	0
NUP214	0	0
NUR77	0	0
N-WASP	0	0
O2	0	0
Omi	0	0
p107	0	0
p110Ia	0	0
p120	0	0
P130	0	0
P130Cas	0	0
P13K	1	0
P1433	0	0
p14ARF	0	0
P15	0	0
p16	0	0
p190RhoGAP	0	0
P2	1	0
P21	0	0
p25	0	0
p27	0	0
p300	0	0
p32	0	0
p34	0	0
P35	0	0
P35611	0	0
p38	1	0
p38gamma	1	0
P50	0	0
p53	1	0
p55gamma	1	0
p6	1	0
p65	0	0
p67phox	0	0
p68	0	0
p70s6K	0	0
P73	0	0
p85	0	0
p85beta	1	0
p90RSK	0	0

PA	0	0
PABP	0	0
PAC1	0	0
PACAP	0	0
PAF	0	0
PAFR	0	0
PAG	0	0
PAK1	0	0
PAK2	0	0
PAK6	0	0
PAR1	1	0
PAR2	0	0
PAR4	0	0
PARD3	0	0
PARP	1	0
PATZ	0	0
PAX2	0	0
PAX5	0	0
PAX8	0	0
PBP	1	0
PCAF	1	0
PCLy	0	0
PCNA	0	0
PDE	0	0
PDE1A	1	0
PDE1B	1	0
PDE1C	1	0
PDE2	1	0
PDE3A	1	0
PDE3B	1	0
PDE4A	1	0
PDE4B	1	0
PDE4C	1	0
PDE4D	1	0
PDE5A	1	1
PDE6A	1	0
PDE6B	1	0
PDE6G	1	0
PDGF	0	0
PDGFB	0	0
PDGFR	1	0

PDGFRA	1	0
PDI	1	0
PDK1	1	0
PDK2	1	0
PDPK1	1	0
PDZGEF	0	0
PDZK1	0	0
PELP1	0	0
PEN2	0	0
PER	0	0
PERK	0	0
PFN1	1	0
pGC	0	0
PGC1A	0	0
PHKA2	0	0
PI3K	1	0
PI3Ky	1	0
PI3P	0	0
PI-4-P5K	0	0
PIAS	0	0
PIAS3	0	0
PIAS4	0	0
PIB5PA	0	0
PICCOLO	0	0
PICK1	0	0
PIK3C2B	0	0
PIK3CD	1	0
PIM2	0	0
PIN1	1	0
PIP2	0	0
PIP3	0	0
PIP5K	0	0
PIR	0	0
PITPN	1	0
PKA	1	0
PKAc	0	0
PKC	0	0
PKCA	1	0
PKCE	0	0
PKCz	0	0
PKD	0	0

PKG	0	0
PKI	1	0
PKLR	1	0
PKN	0	0
PKN2	0	0
PKR	0	0
PLA2	1	0
PLA2P	0	0
PLAGL1	0	0
PLASMIN	0	0
PLC	1	0
PLCb	0	0
PLCD1	1	0
PLCE	1	0
PLCE1	0	0
PLCg-2	0	0
PLCy	0	0
PLD	1	0
PLD1	1	0
plectin	0	0
PLG	1	0
PLK1	1	0
PLK3	0	0
PLSCR1	0	0
PMCA	0	0
POFUT1	0	0
POLR1B	0	0
POLR2A	0	0
POR1	0	0
POTASSIUM	0	0
PP1	0	0
PP2A	0	0
PP2B	0	0
PP2C	0	0
PP32	0	0
PP5	0	0
PPARA	1	1
PPARB	0	0
PPARG	1	1
PPP1CC	1	0
PPP2CA	1	0

PPP2R2A	1	0
PPP2R5B	0	0
PPP2R5C	1	0
PPP3R2	1	0
PQCaCh	0	0
PR-A	0	0
PRAK	0	0
PRF1	0	0
PRK2	0	0
PRKAR1A	1	0
PRKAR1B	0	0
PRKCD	1	0
PRKCG	1	0
PRKCI	1	0
PRMT1	1	0
PROFILIN	0	0
PROKR1	0	0
PrP	1	0
PSD93	0	0
PSD95	0	0
PSEN1	0	0
PSEN2	0	0
PSMC4	0	0
PSMD4	0	0
PTC	0	0
PTCH	0	0
PTCH2	0	0
PTEN	0	0
PTK	1	0
PTK6	1	0
PTMA	0	0
PTP	0	0
PTP1B	1	0
PTP1D	0	0
PTPA	1	0
PTPB1	1	0
PTPN12	0	0
PTPRO	0	0
PTP-SL	0	0
Puma	0	0
PXN	0	0

PYGB	1	0
Pygo	0	0
RAB3	0	0
RAB5	0	0
RABGDI	0	0
RABPHILIN	0	0
RAC1	1	0
RAC3	0	0
RACK	0	0
RAD1	0	0
RAD17	0	0
RAD50	0	0
RAD51	1	0
Rad54l2	0	0
RAD9	0	0
RADIXIN	0	0
RAF1	1	0
RAIDD	0	0
RAL	0	0
RALB	0	0
RALBP1	0	0
RALGDS	0	0
RAN	1	0
RANBP1	0	0
RANBP2	0	0
RANBP9	0	0
RanGAP	0	0
RAP1	0	0
RAP1A	0	0
RAP1B	0	0
RAP1GAP	0	0
RAP2	1	0
RAP30	0	0
RAP74	0	0
RAR	1	0
RAS	0	0
RASGAP	0	0
RASGRF	0	0
RasGRP	0	0
RASGRP1	0	0
RASGRP3	0	0

RB	1	0
RBBP7	0	0
REA	0	0
RELA	1	0
RELN	0	0
REPS1	0	0
REPS2	0	0
REQ	0	0
RET	1	0
RGS	0	0
RGS16	0	0
RGS2	0	0
RGS4	0	0
RHEB	1	0
Rho	1	1
RHOA	1	0
RhoGDP	0	0
RHOGEFs	0	0
RhoGTP	0	0
RIC	0	0
RICS	0	0
RIIa	0	0
RIIb	0	0
RIM	0	0
RIN	0	0
RIN1	0	0
RING1	0	0
RIP	0	0
RIP140	1	0
RKIP	0	0
RLC	0	0
RNF4	0	0
RNPK	0	0
RNTRE	0	0
ROC1	0	0
ROCK1	1	0
ROCK2	1	0
ROR2	0	0
ROS	0	0
RPS6	0	0
RPTK	1	0

RSK	1	0
RSK2	0	0
RTKN	0	0
RTYPECA	0	0
RUVBL1	0	0
RXR	1	0
Ryk	0	0
RYR	1	1
S1P	0	0
S6	0	0
S60	0	0
SAC	0	0
SAG	0	0
SALL1	0	0
SAM68	0	0
SAP102	0	0
SAP18	0	0
SAP30	0	0
SAP97	0	0
SAPAP	0	0
Sara	0	0
SCF	0	0
SDF1	1	0
SEC61P	0	0
SEC63P	0	0
SEK	0	0
SENP1	0	0
SEROTONIN	0	0
SERPINC1	1	0
Ser-prot	0	0
SET	0	0
SF1	1	0
SFN	0	0
SH2D3C	0	0
SH3BGRL	0	0
SH3GL3	0	0
SH3KBP1	0	0
SHANK	0	0
SHARP	0	0
SHC	1	0
SHH	0	0

SHIP	0	0
SHIP2	0	0
SHOC2	0	0
SHP	1	0
SHP1	0	0
SHP2	1	0
SIAH	0	0
Sin3	0	0
SIVA1	0	0
SKI	0	0
SKIP	0	0
SKP1	1	0
SKP2	0	0
SLC25A4	1	0
SLC9A1	1	0
SLP-76	0	0
SMAD1	0	0
SMAD2	1	0
SMAD3	0	0
SMAD4	0	0
SMAD5	0	0
SMAD6	0	0
SMAD7	0	0
SMARCA4	0	0
SMO	1	0
SMPD1	1	0
SMPD2	0	0
SMRT	0	0
SMURF1	0	0
SMURF2	0	0
SNAI1	0	0
SNAP25	1	0
SNAPIN	0	0
SNCA	0	0
SNIP1	0	0
SnoN	0	0
SNRPD2	0	0
SNW1	0	0
SNX1	0	0
SNX2	0	0
SNX4	0	0

SNX6	0	0
SOCS	0	0
SOCS1	0	0
SOCS3	0	0
SOCS5	0	0
SOCS6	0	0
SOD	1	0
SODD	0	0
SODIUM	0	0
SORCIN	0	0
SOS1	0	0
SOS2	0	0
SOX1	0	0
SP1	0	0
SP3	0	0
SPAL	0	0
SPDEF	0	0
SPECTRIN	0	0
SPHK1	0	0
SPHK2	0	0
SPINOPHILIN	0	0
SPRED1	0	0
SPRED2	0	0
SPRY	0	0
SPTAN1	0	0
SRA1	0	0
SRC	1	0
SRE	0	0
SRF	0	0
SRP54	0	0
SRPR	0	0
SRY	0	0
SS	0	0
SSCAM	0	0
SSTR1	1	0
SSTR2	1	0
STAMBPL1	0	0
STAP1	0	0
STARGAZIN	0	0
STAT	0	0
STAT1	0	0

STAT2	0	0
STAT3	0	0
STAT4	0	0
STAT5	0	0
STAT5B	1	0
STAT6	0	0
STEP	0	0
STK11IP	0	0
STK36	0	0
STRAP	0	0
STUB1	0	0
STXBP1	0	0
SUFU	0	0
SUMO1	0	0
SUMO2	0	0
SV2A	1	0
SVIL	0	0
SYK	1	0
SYNAPSIN	0	0
SYNAPTOBREVIN	1	0
SYNAPTOPHYSIN	0	0
SYNAPTOTAGMIN	0	0
SYNDECAN	0	0
SYNGAP	0	0
SYNTAPHILIN	0	0
SYNTAXIN	0	0
SYNTENIN	0	0
SYNTROPHIN	0	0
T3R	1	0
Ta	1	0
TAB1	0	0
TAB2	0	0
TAK1	0	0
TALIN	0	0
TAMALIN	0	0
TANK	0	0
TAU	0	0
TAX	0	0
Tb	0	0
TBK1	0	0
TBP	0	0

TBPIP	0	0
TBR1	0	0
TBX2	0	0
TCF	1	0
TCF12	0	0
TCF3	0	0
TCF4	0	0
TCF7	0	0
TCR	0	0
TEC	0	0
TEP1	0	0
TERT	1	0
TFDP2	0	0
TFF1	0	0
TFF2	1	0
TFIIB	0	0
TFIID	0	0
TFIIH	0	0
TGFB	1	0
TGFB2	0	0
TGFB3	1	0
TGFBR1	1	0
TGFBR2	1	0
TGFBR3	0	0
TGFBRAP1	0	0
TGIF	0	0
TGM2	1	0
THC	1	0
THRAP2	0	0
TIAM1	0	0
TID1	0	0
TIEG2	0	0
TIF2	1	0
TIP1	0	0
TIRAP	0	0
TLR1	0	0
TLR10	0	0
TLR2	1	0
TLR3	0	0
TLR4	1	0
TLR5	0	0

TLR6	0	0
TLR7	1	1
TLR8	1	0
TLR9	1	0
TNF	1	1
TNFB	1	0
TNFR1	1	0
TNFR2	1	0
TNIP1	0	0
TNKS	0	0
TOB	0	0
TOLLIP	0	0
TopoII	1	0
TOR	0	0
TP53BP1	0	0
Tpl-2	0	0
TR4	0	0
TRADD	0	0
TRAF1	0	0
TRAF2	0	0
TRAF3	0	0
TRAF6	0	0
TRE	0	0
TREH	0	0
TRIO	0	0
TRKB	1	0
tRNA	0	0
TRX	0	0
TRYPSIN	1	0
TSA	1	0
TSAP6	0	0
TSC1	0	0
TSC2	0	0
Tsh	0	0
TSP1	0	0
TUBB4Q	1	0
TUBBY	0	0
TUBULIN	1	0
TWIST	0	0
Ty	0	0
TYK2	1	0

Ub	0	0
UBC9	0	0
UBE1L	0	0
UBE2D1	0	0
UBE2D2	1	0
UBE2D3	0	0
UBE2M	0	0
UBE3A	0	0
UbiL	0	0
UXT	0	0
VAMP	0	0
VANGL2	0	0
VASP	0	0
VAV	0	0
VAV2	0	0
VAV3	0	0
VCL	0	0
VDAC2	1	0
VDR	1	1
VEGF	1	0
VEGFR	0	0
VELI1	0	0
VIL2	0	0
VILIP	0	0
VIM	0	0
VIP	0	0
VITRONECTIN	1	0
VLDLR	0	0
VPAC2R	0	0
VRK1	0	0
WAVE1	0	0
WAVE2	0	0
WAVE3	0	0
WDR12	0	0
WIF1	0	0
WNT	0	0
WNT2	0	0
WNT3	0	0
WNT4	0	0
WNT5A	0	0
WNT6	0	0

WNT7A	0	0
XAF1	0	0
XAP2	0	0
XDH	1	0
XIAP	1	0
YAP1	0	0
YES	1	0
YOTIAO	0	0
YY1	0	0
ZAK	0	0
ZAP70	1	0
ZEB1	0	0
ZFHX1B	0	0
ZFYVE9	0	0
ZNF259	0	0
Zyxin	0	0
